# Spatial and temporal distribution of *Ixodes scapularis* and tick-borne pathogens across the northeastern United States

**DOI:** 10.1186/s13071-024-06518-9

**Published:** 2024-11-22

**Authors:** Lucas E. Price, Jonathan M. Winter, Jamie L. Cantoni, Duncan W. Cozens, Megan A. Linske, Scott C. Williams, Griffin M. Dill, Allison M. Gardner, Susan P. Elias, Thomas F. Rounsville, Robert P. Smith, Michael W. Palace, Christina Herrick, Melissa A. Prusinski, Patti Casey, Eliza M. Doncaster, Joseph D. T. Savage, Dorothy I. Wallace, Xun Shi

**Affiliations:** 1 Department of Geography, Dartmouth College, 6017 Fairchild, Hanover, NH 03755 USA; 2 Department of Entomology, The Connecticut Agricultural Experiment Station, 123 Huntington Street, New Haven, CT 06511 USA; 3 Department of Environmental Science and Forestry, The Connecticut Agricultural Experiment Station, 123 Huntington Street, New Haven, CT 06511 USA; 4 Diagnostic and Research Laboratory, University of Maine Cooperative Extension, 17 Godfrey Drive, Orono, ME 04473 USA; 5 School of Biology and Ecology, University of Maine, 5722 Deering Hall, Orono, ME 04469 USA; 6 Vector-borne Disease Laboratory, MaineHealth Institute for Research, 81 Research Drive, Scarborough, ME 04074 USA; 7 Institute for the Study of Earth, Oceans and Space, Department of Earth Sciences, University of New Hampshire, Morse Hall, Durham, NH 03824 USA; 8 Vector Ecology Laboratory, Bureau of Communicable Disease Control, New York State Department of Health, Biggs Laboratory C-456–C-470A, Wadsworth Center, Empire State Plaza, Albany, NY 12237 USA; 9 Environmental Surveillance Program, Vermont Agency of Agriculture Food & Markets, 116 State Street, Montpelier, VT 05620 USA; 10 Graduate Program in Ecology, Evolution, Environment, and Society, Dartmouth College, Hanover, NH 03755 USA; 11 Department of Mathematics, Dartmouth College, 6188 Kemeny Hall, Hanover, NH 03755 USA

**Keywords:** *Ixodes scapularis*, Pathogen prevalence, *Borrelia burgdorferi*, Northeastern United States

## Abstract

**Background:**

The incidence of tick-borne diseases is increasing across the USA, with cases concentrated in the northeastern and midwestern regions of the country. *Ixodes scapularis* is one of the most important tick-borne disease vectors and has spread throughout the northeastern USA over the past four decades, with established populations in all states of the region.

**Methods:**

To better understand the rapid expansion of *I. scapularis* and the pathogens they transmit, we aggregated and analyzed *I. scapularis* abundance and pathogen prevalence data from across the northeastern USA, including the states of Connecticut, Maine, New Hampshire, New York and Vermont, from 1989 to 2021. Maine was the only state to collect data during the entire time period, with the other states collecting data during a subset of this time period starting in 2008 or later. We harmonized *I. scapularis* abundance by county and tick season, where the nymph season is defined as May to September and the adult season is October to December, and calculated *I. scapularis* pathogen infection prevalence as the percentage of ticks that tested positive for *Anaplasma phagocytophilum*, *Babesia microti*, *Borrelia burgdorferi*, and *Borrelia miyamotoi*. We then explored temporal trends in *I. scapularis* abundance and pathogen prevalence data using linear models.

**Results:**

The resulting dataset is one of the most spatially and temporally comprehensive records of tick abundance and pathogen prevalence in the USA. Using linear models, we found small or insignificant changes in the abundance of nymphs and adults over time; however, *A. phagocytophilum*, *B. microti* and *B. burgdorferi* prevalence in both nymphs and adults has increased over time. For the period 2017–2021, the statewide average prevalence of *B. burgdorferi* ranged from 19% to 25% in *I. scapularis* nymphs and from to 49% to 54% in *I. scapularis* adults. The statewide average prevalence of all other pathogens in *I. scapularis* for 2017–2021, including *A. phagocytophilum* (4–6% for nymphs, 4–9% for adults), *B. microti* (4–8% for nymphs, 2–13% for adults) and *B. miyamotoi* (1–2% for nymphs, 1–2% for adults), was considerably less.

**Conclusions:**

Our efforts revealed the complications of creating a comprehensive dataset of tick abundance and pathogen prevalence across time and space due to variations in tick collection and pathogen testing methods. Although tick abundance has not changed along the more southern latitudes in our study over this time period, and only gradually changed in the more northern latitudes of our study, human risk for exposure to tick-borne pathogens has increased due to increased pathogen prevalence in *I. scapularis*. This dataset can be used in future studies of *I. scapularis* and pathogen prevalence across the northeastern USA and to evaluate models of *I. scapularis* ecology and population dynamics.

**Graphical Abstract:**

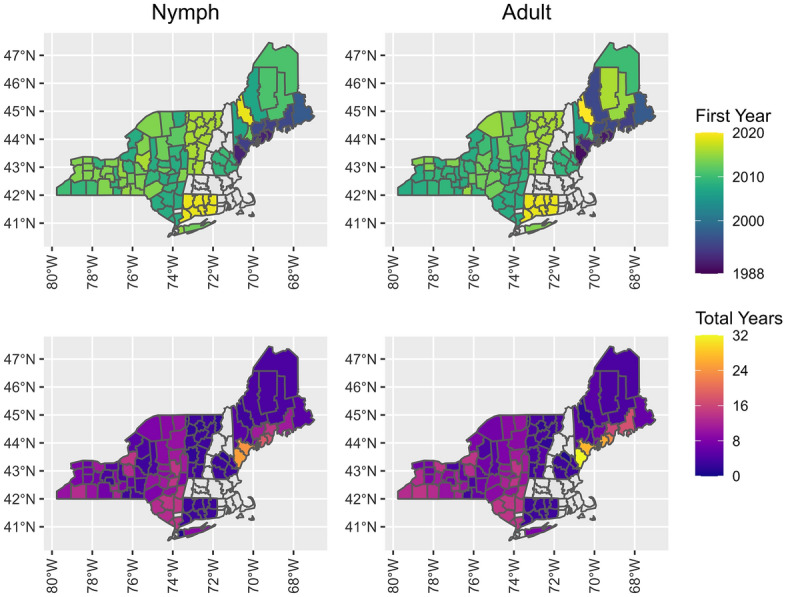

**Supplementary Information:**

The online version contains supplementary material available at 10.1186/s13071-024-06518-9.

## Background

Tick-borne diseases accounted for 77% of all vector-borne disease reports in the USA from 2004 to 2016, and 82% of these reported tick-borne disease cases were Lyme disease [1]. Lyme disease case counts have increased in the USA since it was first described in 1975 in Connecticut, and in 2019 there were 23,453 confirmed and 11,492 probable cases reported nationally to the U.S. Centers for Disease Control and Prevention (CDC) [2]. However, cases are underreported, and Kugeler et al. [3] estimated that as many as 476,000 people are diagnosed and treated for Lyme disease in the USA each year, based on insurance claims data. Lyme disease has high endemicity in the northeastern USA, where the tick *Ixodes scapularis* is the primary vector for spreading the causative spirochete bacterium, *Borrelia burgdorferi* [2]. *Ixodes scapularis* transmits several other tick-borne pathogens identified in the northeastern USA, including the bacterium *Anaplasma phagocytophilum*, which causes anaplasmosis; the parasite *Babesia microti*, which causes babesiosis; and the bacterium *Borrelia miyamotoi*, which causes hard tick relapsing fever [2]. There were 2418 cases of babesiosis and 5655 cases of anaplasmosis reported nationally to the CDC in 2019 [2]. *Borrelia miyamotoi* is an emerging pathogen in the northeastern region of the USA, and states do not currently report human case counts of hard tick relapsing fever to the CDC [2].

Many studies have examined the range and environmental associations of *I. scapularis* due to its medical significance. Dennis et al. [4], Eisen et al. [5] and Eisen and Eisen [6] summarized past surveys and records from active surveillance (field collections of questing or feeding ticks) and passive surveillance (ticks sent by the public to laboratories for identification) across the continental USA, 1907–2015. Data sources used in their research included the CDC questionnaires, published literature, the USA National Tick Collection and other supplemental sources. These works defined *I. scapularis* as “established” in a county if > 1 life stage (larvae, nymph, adult) or at least six ticks were detected within a calendar year, and as “reported” if at least one tick of any life stage was detected within 1 year [4]. Since the publication of Dennis et al. [4] in 1998, all but five counties in the northeastern USA (states of Connecticut, Maine, New Hampshire, New York and Vermont) were classified as having established tick populations by 2015 [5]; by 2022, all but two counties in the northeastern USA were classified as having established tick populations [6]. The research of these authors [4–6] revealed unique insights by combining multiple data sources, but only determined tick status as established, reported or not reported, and therefore did not quantify relative abundance of ticks within counties.

Several active surveillance research studies on *I. scapularis *have taken place over large portions of the USA. Diuk-Wasser et al. [7, 8] and Pepin et al. [9] describe the most extensive field-based study of nymphal *I. scapularis* abundance in the eastern USA to date, based on active surveillance of questing *I. scapularis* nymphs via drag surveys from 2004 to 2006, with 301 sample sites across 37 states. Bunnell et al. [10] surveyed questing adult *I. scapularis* along 320 transects across five states in the Mid-Atlantic region of the USA in 1997 and 1998 and found spatial clustering of adult tick abundance in the region. However, each of these active surveillance studies focused on a single tick life stage and had limited temporal coverage compared to the reviews of Dennis et al. [4] and Eisen et al. [6].

In the present study, we developed a harmonized, county-level tick abundance and pathogen prevalence dataset for most of the northeastern USA (Connecticut, Maine, New Hampshire, New York and Vermont) that draws from active surveillance of questing ticks and covers multiple decades. This dataset includes nymph and adult life stages and infection prevalence of four pathogens: *A. phagocytophilum*, *B. microti*, *B. burgdorferi* and *B. miyamotoi*. We then applied linear models to evaluate temporal trends in abundance and pathogen prevalence throughout the region. This provides a unique dataset compiled throughout the northeastern USA that adds to the body of *I. scapularis* abundance and pathogen prevalence surveillance literature and has multiple research and public health applications. The standardization of these datasets also highlights the difficulty and necessary compromises when diverse sampling protocols are used across jurisdictional borders.

## Methods

In 2019, the U.S. CDC established guidelines for county-level *I. scapularis* surveillance [11]. These include sampling (i) at a minimum of 750 m during the peak tick host-seeking activity period and suitable weather conditions; (ii) at least one site per county required with multiple sites recommended; and (iii) multiple times per site, with ≥ 3 replicates recommended during each life stage peak of host-seeking activity. However, prior surveillance from state programs was not standardized, and the current implementation of CDC guidelines is not identical among states, leading to variation in sampling efforts and techniques. In this section, we describe the tick surveillance protocols used in each state and our process for harmonizing the datasets.

### Connecticut tick surveillance

Sampling of *I. scapularis* nymphs and adults in Connecticut was conducted by The Connecticut Agricultural Experiment Station between 2019 and 2021 (2019–2021), following CDC protocols [11]. Ticks were collected in all eight counties of the state using drag surveys, with five sites per county for a total of 40 regularly visited sites. Sites were surveyed from late March through November, with each survey conducted in 750-m^2^ linear transects along public trails and ecotonal edge habitat using a 1-m^2^ cloth attached to a wooden dowel that was passed over and through vegetation. All ticks collected were identified morphologically using a compound microscope, and all adult *I. scapularis* females and nymphs were temporarily retained in a humidified container and stored in a refrigerator until they were tested. Ticks were identified by professionals trained on the identification of species and life stage of ticks in Connecticut. A total of 2136 adult females and 2915 nymphs were collected in Connecticut in the 2019–2021 period, with sites sampled 6 times in 2019, 7 times in 2020 and 5 times in 2021.

*Ixodes scapularis* adult females and nymphs collected between 2019 and 2021 were tested individually in batches of 96 at The Connecticut Agricultural Experiment Station. Bacterial DNA and viral RNA were extracted from 200 μl of tick homogenate using the MagMAX Viral/Pathogen Nucleic Acid Isolation Kit (Thermo Fisher Scientific, Waltham, MA, USA) and eluted into 50 μl of elution solution. The nucleic acid was then subjected to a multiplex real time reverse-transcription PCR (RT-PCR) for four pathogens associated with* I. scapularis*:* B. burgdorferi* sensu lato (*B. burgdorferi* s.l.),* B. miyamotoi*,* B. microti* and *A. phagocytophilum*. Primer and probe sets had previously been established for all four pathogens [12]. The reaction mixture (total volume 20 μl) for the real time RT-PCR assays contained 5.0 μl 4× TaqPath One-Step Multiplex Mix (Thermo Fisher Scientific), 1.0 μl of our* Ixodes* primer mix (10 μM), 0.4 μl of our* Ixodes* probe mix (10 μM), 11.6 μl of nuclease-free water and 2.0 μl of tick extract. The real time RT-PCR assays were then run at the following conditions: 2 min at 25 °C; 10 min at 53 °C; 2 min at 95 °C; followed by 40 cycles of 15 s at 95 °C and 60 s at 60 °C. Samples that tested positive for *B. burgdorferi* s.l. were then tested against strain-specific primers and probes to differentiate between *B. burgdorferi* sensu stricto (*B. burgdorferi* s.s.) and *B. mayonii* [13]. A total of 1561 adult females and 1787 nymphs were tested in Connecticut between 2019 and 2021.

### Maine tick surveillance

Sampling of *I. scapularis* adults and nymphs in Maine was conducted for all counties throughout the state by MaineHealth Institute for Research (MHIR) from 1989 to 2022 (1989–2022, in surveys conducted by researchers at the University of Maine), with additional surveys conducted in southern and coastal Maine (Androscoggin, Cumberland, Hancock, Knox, Kennebec, Lincoln, Sagadahoc, Waldo and York counties) by researchers at the University of Maine in 2020 and 2021. MHIR used the flagging collection method (1-m^2^ cloth passed over and through vegetation) and recorded time flagged prior to 2019 and both time and area flagged 2019–2022 per CDC protocols [11]. MHIR adult data were summarized for peak adult season in Maine (April/May and October/November) and nymph data were summarized for peak nymph season in Maine (June/July). Sampling efforts covered 790 sites in 194 towns in all 16 counties across 35 years (1224 days). The MHIR identified 45,899 adult ticks and 9190 nymph ticks using keys by Cooley and Kohls [14] and Durden and Keirans [15]. The University of Maine collected ticks during July and August of 2020 and 2021 via dragging surveys with a 1-m^2^ cloth attached to a wooden dowel passed over and through vegetation [16]. Ticks were identified using keys by Durden and Kierans [15] and Kierans and Litwak [17]. In 2020, the University of Maine drag surveys collected a total of 292 adults and 459 nymphs, averaging 4.8 nymphs per property; in 2021, these surveys collected a total of 234 adults and 2107 nymphs, averaging 14.8 nymphs per property in 2021.

*Ixodes scapularis* collected by MHIR between 1990 and 2015 (1990–2015) were tested individually (35,534 peak-season adults and 3,096 peak-season nymphs) at MHIR for *Borrelia spp.* by direct immunofluorescence microscopy (DFA) [18]. DFA did not discriminate between *B. burgdorferi* and *B. miyamotoi* Fukunaga, but we believe this would not affect our inferences because the prevalence of *B. miyamotoi* in Maine is low, approximately 3.6% in female *I. scapularis* [19]. Nymphs collected in 2019 (MHIR) and in 2020–2021 (University of Maine) were tested individually for multiple pathogens at the University of Maine Cooperative Extension Diagnostic and Research Laboratory using a single real-time quantitative PCR (qPCR) multiplex reaction [20]. The *B. burgdorferi* s.l. assay targeted the* 23S* ribosomal RNA (rRNA) gene and amplified any species in the *B. burgdorferi* s.l. complex, as well as *B. miyamotoi* [21]. The target of the *A. phagocytophilum* assay was the major surface protein 2 (*msp2*) gene [21]. The *B. microti* assays differed between 2020 and 2021, but both assays targeted the 18S gene. In 2020, the primer and probe set used was from Wang et al. [22]; for assays performed in 2021, this was changed to the set used by Hojgaard et al. [23]. In addition, a second qPCR probe (unpublished) was added to the Hojgaard et al. [23] assay to increase sensitivity: 5′–Texas Red 615—TCCGAATAATTCACCGGATCACTC–3′.

### New Hampshire tick surveillance

Sampling of *I. scapularis* adults and nymphs in New Hampshire was conducted by the University of New Hampshire between 2009 and 2011 (2009–2011) and did not follow CDC protocols [11]. Ticks were collected at 36 field sites in five of the 10 counties in the state (Strafford, Rockingham, Belknap, Merrimack and Hillsborough), all located in the southeastern part of New Hampshire. The sites were 2–5 acres (0.81–2.02 ha) in size and chosen based on forest cover type, proximity to roads and footpaths and public accessibility. The sites were visited and measured 9–12 times between May and November. All sites were randomly visited but always within 2 weeks of each other throughout the period of study. At each location, two 76.2-m transects separated by 80 feet were dragged using a 1-m^2^ piece of white cloth over leaf litter and low-lying vegetation. Collected ticks were examined using an LW Scientific Z2 Zoom trinocular stereoscope (LW Scientific, Lawrenceville, GA, USA) to determine the species of each tick along with its life stage and sex, and then stored in a 70% ethanol solution until they could be sent for testing. Ticks were identified by professionals who received training from the New Hampshire State Entomologist. Each tick was then assigned a unique identifier and sent to the New Hampshire Public Health Laboratory (PHL) in Concord, NH. A total of 181 nymph and 967 adult *I. scapularis* were collected.

All *I. scapularis* adults and nymphs collected in 2009–2011 were tested by PHL using qPCR to detect *B. burgdorferi* s.l. DNA in individual ticks. The ticks were processed using a Retsch Mixer Mill (Retsch, Haan, Germany), centrifuged, and then an aliquot was sent for DNA extraction. The DNA was extracted using the Corbett X-tractor gene instrument (automated method; Corbett Research, Mortlake, Australia), or the Qiagen DNA mini-kit (manual method; Qiagen, Hilden, Germany). PCR assays were performed using the Roche Lightcycler Faststart kit (Roch Diagnostics, Rotkreuz, Switzerland) and amplified on the ABI 7500 Fast DX platform (Thermo Fisher Scientific). Pathogen prevalence for ticks in New Hampshire only included *B. burgdorferi*.

### New York tick surveillance

Sampling of *I. scapularis* adults and nymphs in New York was conducted by New York State Department of Health between 2008 and 2021 (2008–2021), following CDC protocols [11]. Host-seeking *I. scapularis* were collected using standardized drag surveys conducted on publicly accessible land in 56 of 57 counties outside of New York City. *Ixodes scapularis* nymphs were collected during April–September by dragging a 1-m^2^ piece of white flannel through leaf litter and low-lying vegetation. *Ixodes scapularis* adults were collected during September–December by flagging a 1-m^2^ piece of white canvas over edge ecotone and understory vegetation up to a height of 1 m [24]. Tick collection sites were selected based on ≥ 1 of the following criteria: northern hardwood forest type; suitable habitat for *I. scapularis,* other tick species of interest, white-tailed deer and small mammals, including *Peromyscus* species mice; potential for human exposure to ticks; and epidemiological links to locally acquired cases of rare or emerging tick-borne illness. Most tick surveillance sites were located on state, county or town land, such as wildlife management areas, nature preserves and parks. Sampling occurred at locations both with and without established tick populations, including sites where ticks were absent or at population levels below the limit of detection by drag surveillance, and at locations beyond the reported geographic range of certain tick-borne illnesses that are considered endemic in New York State. A minimum of 1000 m^2^ of suitable tick habitat was sampled during most sampling events, with the number of surveys per county varying. Ticks were stored in 100% ethanol at 4 °C until they were sorted by developmental stage and identified to species by dichotomous keys [15, 25], placed into sterile microcentrifuge tubes containing 100% ethanol and stored at − 20 °C until DNA extraction. A total of 157,947 *I. scapularis* nymphs and adults were collected in New York 2008–2021.

Individual ticks were processed for DNA extraction as described in Prusinski et al. [24] and Piedmonte et al. [26] using the DNeasy Blood and Tissue kit (Qiagen) following the manufacturer’s recommendations for the supplementary protocol: “Purification of Total DNA from Insects.” After individual *I. scapularis* ticks underwent total genomic DNA extraction, they were tested for *A. phagocytophilum* (major surface protein 2 [*msp2*]), *B. microti* (*18S* rDNA), *B. burgdorferi* (*16S* rDNA), and *B. miyamotoi* (*16S* rDNA) using a quadplex qPCR [24, 26]. All fluorogenic probes and primers were synthesized by Integrated DNA Technologies (Coralville, IA, USA) and Applied Biosystems (Thermo Fisher Scientific). Reactions were prepared in a 1:1 mixture of 5× PerfeCta MultiPlex qPCR ToughMix, Low ROX and 2× PerfeCta MultiPlex qPCR SuperMix (Quanta Biosciences, Gaithersburg, MD, USA). Each run included a negative control, with nuclease-free water substituted for template DNA, and a positive control consisting of 7.8 μl purified pathogen-free *I. scapularis* DNA in nuclease-free water with 0.3 μl each of the following: DNA extracted from whole blood of a *B. microti*-infected C3H/HeN mouse with 10% parasitemia (Stony Brook University, Stony Brook, NY, USA), low-passage *B. burgdorferi* B31 lysate (Stony Brook University), previously isolated *A. phagocytophilum* genomic DNA (U.S. CDC, Atlanta, GA, USA) and *Borrelia hermsii* DNA purified from culture. All qPCR data were analyzed using the Applied Biosystems 7500 SDS software version 1.4 (Applied Biosystems, Thermo Fisher Scientific) and interpreted using criteria described in Piedmonte et al. [26]. A total of 48,617 adults and 28,946 nymphs were tested for pathogens in New York 2008–2021. In New York counties where *I. scapularis* was just emerging, all nymphal and adult ticks collected were tested for pathogens and county-level prevalence values were reported, even when surveillance testing targets (*n* = 25 per CDC guidance) were not met. From 2008 to 2021, the total number of nymphs tested per New York county sampled was < 25 during 98/510 (19.2%) of overall county/seasons for nymphs and 70/555 (12.6%) of county/seasons for adult *I. scapularis*.

### Vermont tick surveillance

Sampling of *I. scapularis* adults and nymphs in Vermont was conducted by the Vermont Agency of Agriculture between 2015 and 2019 (2015–2019). Ticks were collected in all 14 counties of Vermont using drag surveys, following CDC protocols [11]. Sites were distributed throughout the state, with a site in most Vermont towns (approx. 250 in total) sampled once over the course of 5 years, which translates to 50 sites per year and 25 sites per season. Spring sampling was conducted from the beginning of May through mid-June, and fall sampling was conducted mid-October through the end of November. At each site, four 100-m transects were surveyed by dragging a 1-m^2^ piece of fabric. The fabric was inspected every 10 m, and any ticks attached to the fabric were picked off using forceps and placed into a pre-labeled vial. Number, life stage and sex of ticks were recorded for each 10-m length during most surveys. A total of 1793 ticks (*I. scapularis* and *Dermacentor variabilis*) were collected in Vermont 2015–2019.

Once sampling concluded for the season, ticks were identified by species, life stage and sex using an Olympus SZX12 microscope (Olympus Corp., Tokyo, Japan) and keys from Keirans and Litwak [17] and Durden and Keirans [15]. Tick samples were stored in individual vials containing 95% ethanol at 4 °C until processing. Homogenization and nucleic acid extraction were performed using a Retsch Mixer Mill and the MagMax Pathogen RNA/DNA Kit (Thermo Fisher Scientific). Purified nucleic acids were recovered from clarified tick lysate (400 μl, centrifuged 1 min at 14,000 *g*) with Dynabeads. We used assay target sequences from Hojgaard et al. [23] and determined bacterial pathogen presence with a quadruplex qPCR assay for *B. burgdorferi* (flagellar filament cap [*fliD*]); *A. phagocytophilum* (major surface protein [*msp2*]); *B. microti* (surface antigen 1 [*sa1*]); and *B. miyamotoi* (glycerophosphodiester phosphodiesterase [*glpQ*]) [13]. A separate assay utilizing a target to *I. scapularis* actin [23] was run as a DNA-extraction positive control. Assays were run on an ABI 7500 FAST thermocycler (Thermo Fisher Scientific). A total of 1675 *I. scapularis* adults and nymphs were tested in Vermont 2015–2019.

### Tick abundance and pathogen prevalence data processing

*Ixodes scapularis* abundance and pathogen prevalence data were collected using a range of spatial identifiers (exact coordinates to county), temporal resolutions (daily to seasonal), spatial coverages (limited samples in a few counties to multiple samples of all counties), temporal coverage (3–33 years), collection methods (area drag, timed drag and timed flagging) and data aggregation (individual tick vs average population). We prioritized making data as comparable as possible when harmonizing across states, aggregating tick abundance by county using the two seasons defined by the New York State Department of Health [26, 27] linked to the timing of nymph and adult life stage questing periods. The nymph *I. scapularis* season is defined as May to September, and the adult season as October to December. For these seasons, we totaled all *I. scapularis* for the given life stage collected through tick dragging or flagging within a county and divided by the total area sampled.

Maine data were collected as ticks per minute sampled, so we converted time to an approximate area. In 2019, researchers in Maine started recording both time and area sampled for some of their surveys. We used these data in a simple linear regression to predict area flagged as a function of time sampled for each survey, and used estimated area sampled in our Maine tick abundance estimates. The formula we calculated from Maine data to convert timed surveys to distance was:

$${\text{Distance }}\left( {{\text{meters}}} \right){\mkern 1mu} = 907.1 + 21.2*{\text{ Time }}\left( {{\text{minutes}}} \right)$$

Area sampled was positively correlated with time sampled (*R*^2^ = 0.34, *P* < 0.01, *t* = 11.90). We also explored using the square root of time to predict distance, but there was only a negligible increase in *R*^2^ value (0.05), and the model using the square root of time predicted negative area for drag surveys that were very short (a few minutes). Therefore, we used the simpler model. All abundance estimates presented here are ticks per hectare.

We harmonized pathogen prevalence data using a similar approach to that of tick abundance estimation. For Maine, New Hampshire and New York, pathogen prevalence was defined as the number of ticks of a given life stage during the tick season (May–September for nymphs and October–December for adults) that tested positive for a pathogen out of the total number tested. For Vermont, pathogen prevalence was provided with nymph and adult data combined for each survey, so we excluded the few surveys with nymphs when calculating pathogen prevalence for adults (October–December), and we were unable to calculate pathogen prevalence for nymphs. Connecticut pathogen prevalence data were provided as an annual value, instead of by season, with pathogen prevalence calculated as the number of ticks of a given life stage testing positive for a pathogen out of the total number of ticks tested for that life stage for the entire year. By including ticks by year instead of tick season in Connecticut, we included ticks from different cohorts into the same mean, which could impact mean prevalence values. This may have artificially reduced differences on short time scales, but allowed us to expand our dataset. Similar to abundance, we aggregated pathogen prevalence data by county.

We prioritized including as much data as reasonably possible, so did not use lower limits to restrict data that were included in tick abundance and pathogen prevalence calculations. Most counties were sampled multiple times per season, with the vast majority of counties sampled over multiple years. While not setting a lower limit could bias results due to small sample size, we attempted to minimize this possibility by reporting results at the county scale (typically aggregating multiple sites per county) and including data summarizations over the length of record (aggregating over multiple years).

Reported means and standard deviations (SD) were calculated by state across county-seasons. We prioritized consistent data across the northeastern USA, which we concluded was best represented by tick abundance and pathogen prevalence at the county level. However, because we first aggregated by county, reported standard deviations neglect within-county variability, which could be substantial and influenced by the size of the county, number of sites in each county, number of samples in each county and differences in tick habitat and hosts across the county. All data processing and analysis were completed in R [28], the package ‘dplyr’ for summaries and regressions [29] and the package ‘ggplot2’ for graphic visualizations [30].

**Fig. 1:**
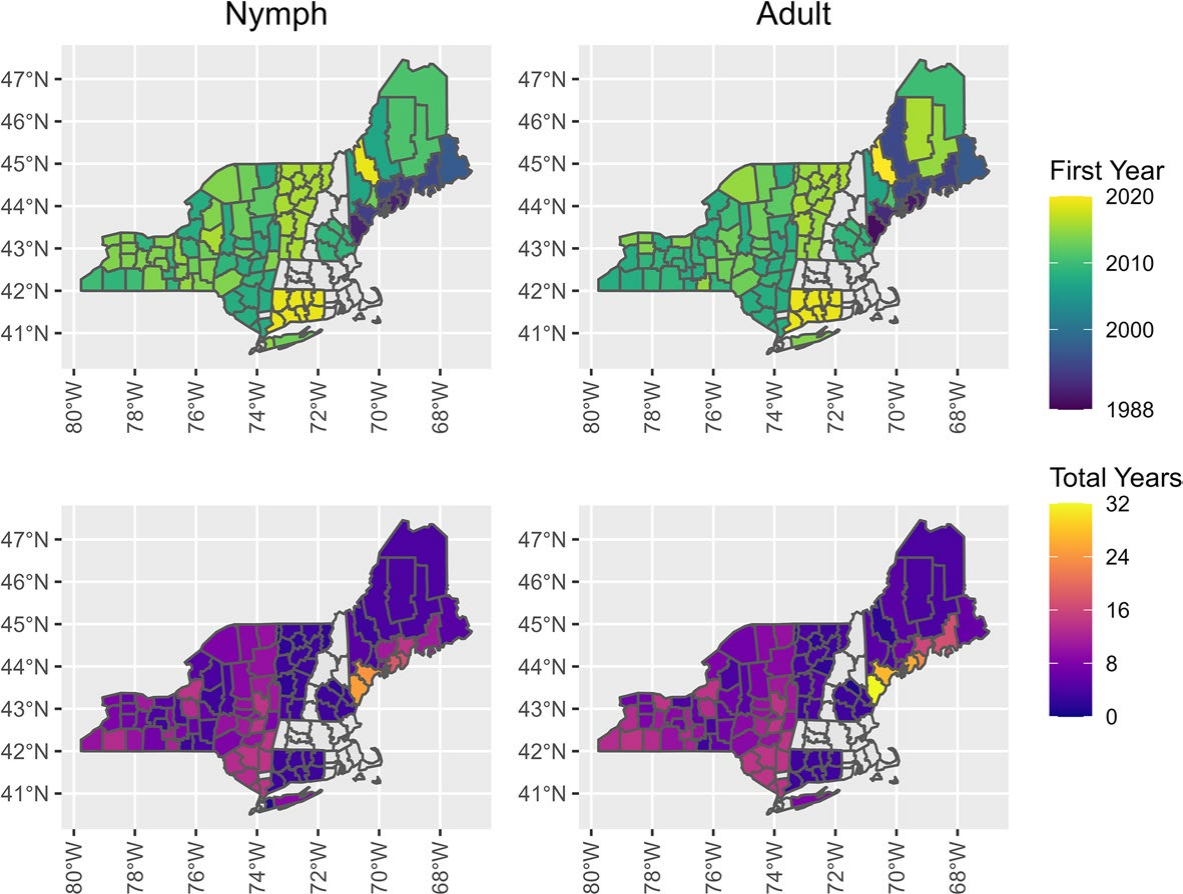
Study area and time period for *Ixodes scapularis* sampling in the northeastern USA by county. Top-left panel shows the first year of sampling for nymphal *I. scapularis*, top-right panel shows the first year of sampling for adult *I. scapularis*, bottom-left panel shows the total number of years sampled for nymphal *I. scapularis* and bottom right panel shows the total number of years sampled for adult *I. scapularis*. Counties without color fill (light gray) were those for which we had no sampling data

We calculated nymph *I. scapularis* abundance (Additional file 1: Table S1) for 731 county-seasons between 1991 and 2022 and adult abundance (Additional file 1: Table S2) for 820 county-seasons between 1989 and 2021 (Table 1; Fig. 1). We calculated nymph *I. scapularis* pathogen prevalence (Additional file 1: Table S3) for 561 county-seasons between 1991 and 2021 and adult pathogen prevalence (Additional file 1: Table S4) for 682 county-seasons between 1990 and 2021. Nymph pathogen prevalence for *B. burgdorferi* was first reported in 1991, *A. phagocytophilum* and *B. microti* were first reported in 2008 and *B. miyamotoi* was first reported in 2015 (Table 1). Adult pathogen prevalence for *B. burgdorferi* was first reported in 1990, while the other pathogens were first reported the same year as for nymphs.

**Table 1:** Data coverage for *Ixodes scapularis* abundance and pathogen prevalence by year for each state included in this study of the northeastern USA

State	Abundance	*Anaplasma phagocytophilum*	*Babesia microti*	*Borrelia burgdorferi*	*Borrelia miyamotoi*
Connecticut	2019–2021	2019–2021	2019–2021	2019–2021	2019–2021
Maine	1989–2022	2020–2021	2020–2021	1990–2021	NA
New Hampshire	2009–2011	NA	NA	2009–2011	NA
New York	2008–2021	2008–2021	2008–2021	2008–2021	2015–2021
Vermont	2015–2019	2015–2019	2015–2019	2015–2019	2016–2019

In Maine, there was no *Borrelia burgdorferi* testing from 2016 to 2018

*NA* Not available

### Temporal trend modeling

Linear models were used to evaluate temporal trends in *I. scapularis* data. To analyze changes in abundance over time with county as the sampling unit, we used year, latitude in decimal degrees at the county centroid (Additional file 1: Table S5) and state as predictor variables, and nymph abundance or adult abundance as the dependent variable. We added one to the abundance data and then log-transformed the data to normalize residuals. We included an interaction term between year and latitude to account for spatial variation in rate of change in tick abundance throughout the region. To analyze changes in pathogen prevalence over time with county as the sampling unit, we used year, latitude and state as predictor variables, and pathogen prevalence of *A. phagocytophilum*, *B. microti*, *B. burgdorferi* or *B. miyamotoi* as the dependent variable, creating separate models for nymph and adult ticks and for each pathogen. As with the abundance models, we included an interaction term between year and latitude. To reduce the influence of the absolute value of year and latitude in the interaction term, we centered both variables in the abundance and pathogen prevalence models. The center year for the abundance models and *B. burgdorferi* was 2005; for *A. phagocytophilum* and *B. microti* models, the center year was 2015; and for *B. miyamotoi* models, the center year was 2018. For latitude, our center was 43.7°. We chose not to scale year or latitude to help the interpretability of the model output, but with scaling the results were qualitatively similar. The state categorical variable was included to account for variation among states not associated with latitude, and this variation could be related to differences in sampling protocols, pathogen detection protocols, or environmental factors. We considered change over time, latitude, state, and interaction term coefficients to be significant with an alpha of 0.05. We considered more sophisticated models but selected linear models for simplicity and ease of interpretation. Analysis was completed in *R* [28], and we used the package ‘emmeans’ for calculating effective slopes [31] which, for this study, was the annual change in the dependent variable taking into account the influence of latitude through the interaction term.

## Results

**Fig. 2:**
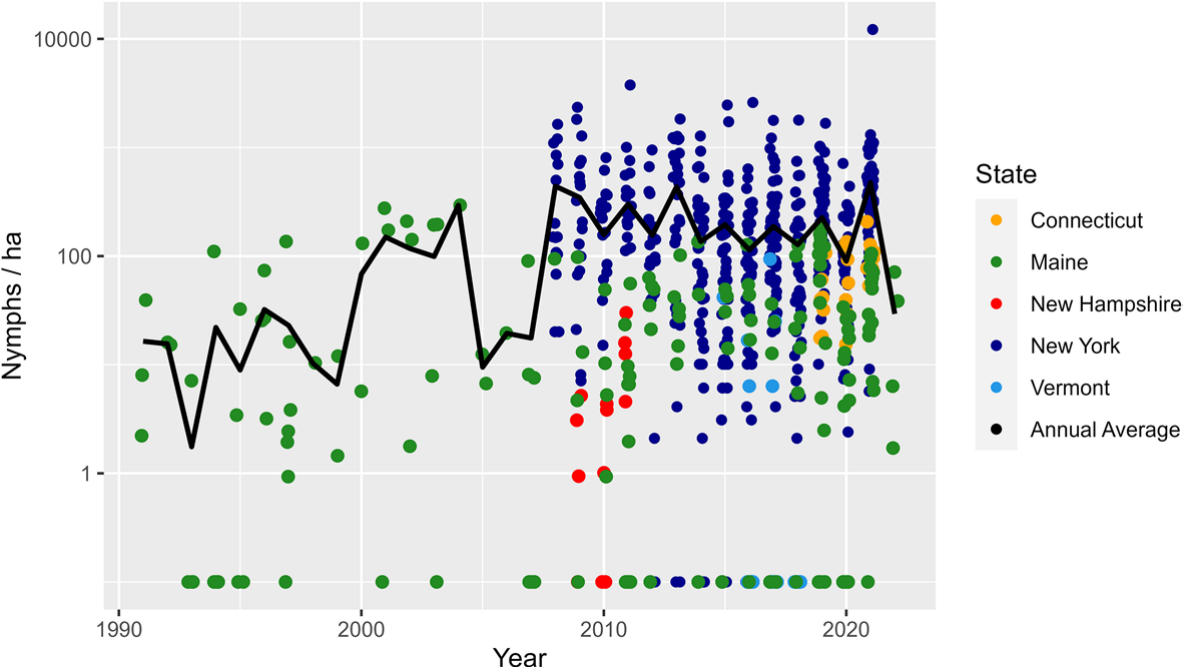
Abundance of nymphal *Ixodes scapularis* per hectare over time in the northeastern USA on a logistic scale, with each point representing a county’s abundance within a season. The nymphal season was from May to September, with the abundance calculated as the total number of nymphs collected divided by the total area sampled during the season within a county

**Fig. 3:**
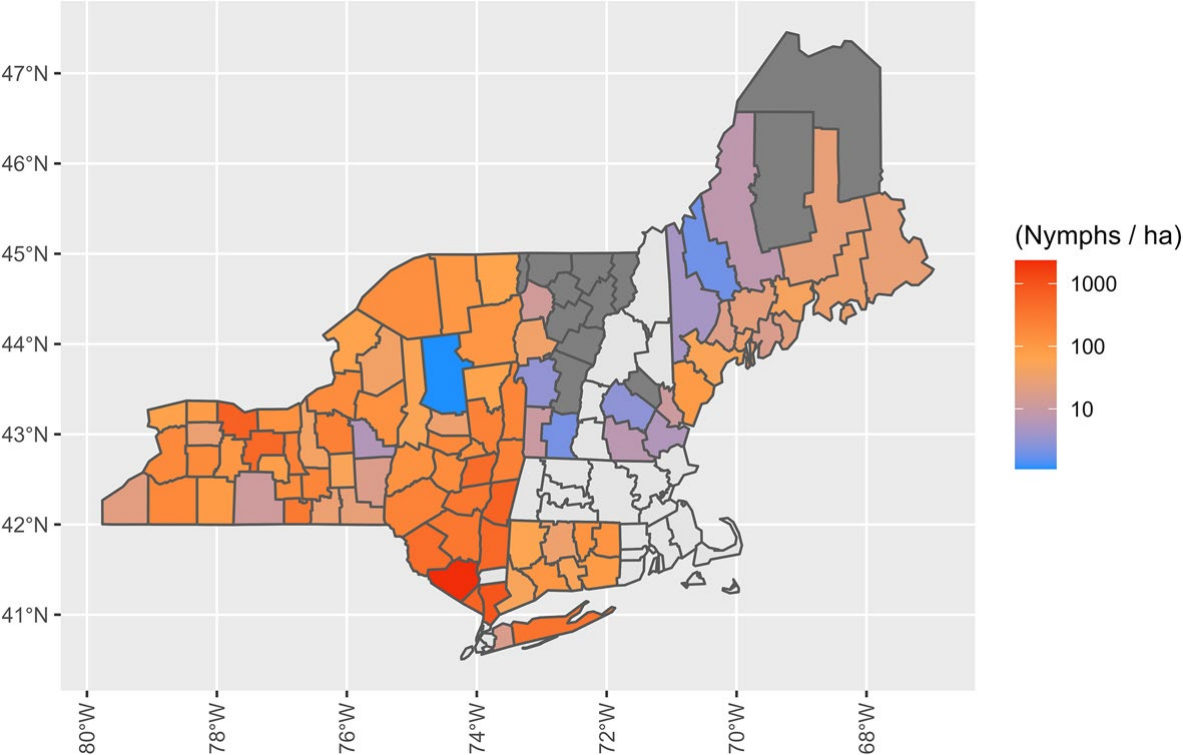
Average abundance of nymphal *Ixodes scapularis* per hectare in the northeastern USA. The nymphal season was from May to September, with the abundance calculated as the total number of nymphs collected divided by the total area sampled during a single season within a county. An average was taken of all abundance estimates for each county, and these are shown using a logistic scale, with dark-gray shading denoting counties that were sampled, but no nymphs found. Counties without color fill (light gray) were those for which we had no sampling data

Averaged across all years, the mean (± SD) *I. scapularis* nymph tick abundance was 293.5 ± 671.1 nymphs per hectare for New York, 79.5 ± 48.0 nymphs per hectare for Connecticut, 38.7 ± 55.1 nymphs per hectare for Maine, 5.8 ± 8.5 nymphs per hectare for New Hampshire and 5.1 ± 17.1 nymphs per hectare for Vermont (Fig. 2). While variance is large and overlaps among states, mean abundance of nymphs was higher in the southern part of the study area, intermediate in Maine and lowest in New Hampshire and Vermont (Fig. 3). Using our model to more precisely look at variance in the dataset, we found a small increase in abundance (ticks per hectare) of *I. scapularis* nymphs over time (*P* < 0.01, *t* = 4.58), with smaller nymph abundance but a larger rate of abundance increase at higher latitudes (Table 2; Additional file 1: Figure S1). With the Year and Year: Latitude interaction model coefficients accounted for, at our lowest latitude (40.75°), the effective increase in log abundance was − 0.03 (95% confidence interval [CI] − 0.11 to 0.04) per year; at the center latitude (43.70°), the effective increase was 0.05 (95% CI 0.03–0.08); and at our highest latitude (46.66°), the effective increase was 0.14 (95% CI 0.06–0.22).

**Table 2:** Linear models evaluating the change in *Ixodes scapularis* abundance (ticks per hectare) and pathogen prevalence over time in the northeastern USA, including latitude as a continuous variable and state as a categorical predictor variable, with an interaction between year and latitude

Model	Year	Latitude	Year:Latitude^a^	*Y*-Intercept	State^b^				*R*^2^
					Maine	New Hampshire	New York	Vermont	
Nymph abundance	0.05*	− 1.34*	0.03*	1.50*	1.39*	− 1.09	1.30*	− 1.27*	0.42
Adult abundance	0.08*	− 1.03*	0.02*	1.64*	2.59*	0.91	2.03*	1.26*	0.27
N. *Borrelia burgdorferi*	0.38*	3.08*	− 0.25*	11.55*	8.53*	1.61	6.85*	NA	0.04
N. *Anaplasma phagocytophilum*	0.23*	− 1.38*	0.01	0.77	4.28*	NA	1.24	NA	0.06
N. *Babesia microti*	0.21*	− 2.75*	− 0.21*	− 1.56	7.35*	NA	1.19	NA	0.28
N. *Borrelia miyamotoi*	− 0.04	− 0.39*	− 0.03	0.63	NA	NA	− 0.19	NA	0.04
A. *B. burgdorferi*	1.72*	− 0.32	− 0.11	19.22*	27.15*	31.92*	7.74	5.14	0.15
A. *A. phagocytophilum*	0.92*	− 3.34*	0.24*	− 0.09	NA	NA	2.40	3.74	0.22
A. *B. microti*	0.37*	− 2.21*	− 0.09	5.21*	NA	NA	− 4.85*	− 3.63*	0.30
A. *B. miyamotoi*	0.05	− 0.57*	0.06	1.15*	NA	NA	− 0.53	− 0.60	0.12

* A* Adult,* N* nymph

*Significant model variables at *p* < 0.05

^a^Year was centered on 2005 for abundance and *Borrelia burgdorferi* prevalence models, on 2015 for *Anaplasma phagocytophilum* and *Babesia microti* prevalence models and on 2018 for *Borrelia miyamotoi* prevalence models. Latitude was centered at 43.7° for all models

^b^Connecticut was absorbed into the intercept of the model

**Fig. 4:**
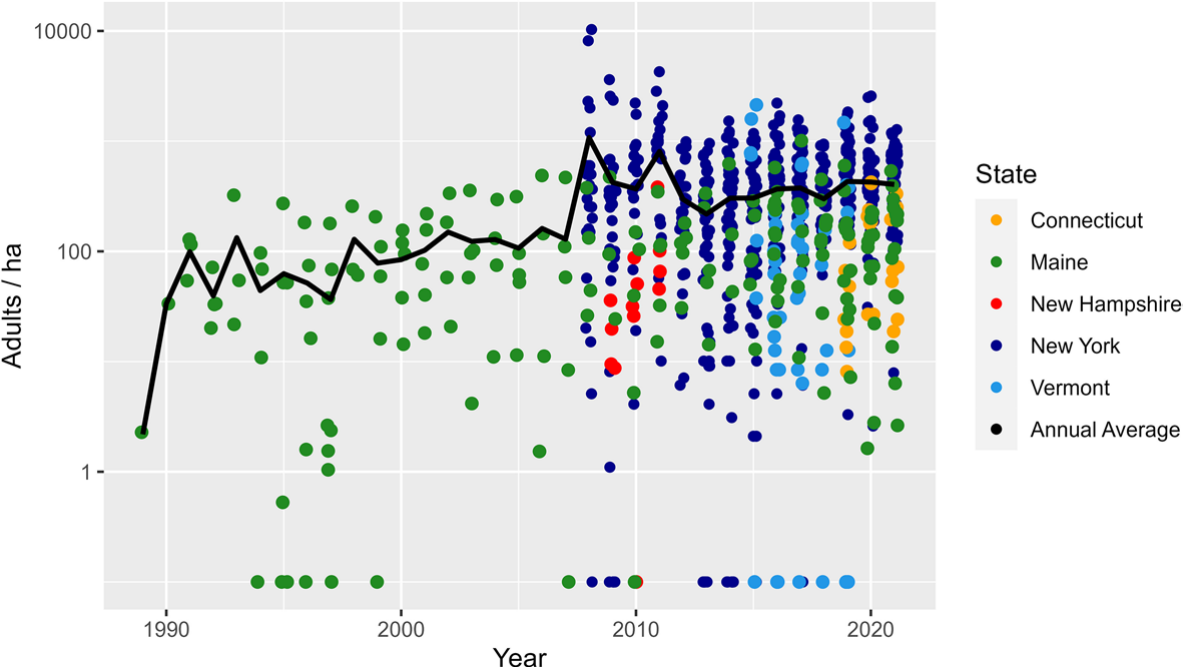
Abundance of adult *Ixodes scapularis* per hectare over time in the northeastern USA on a logistic scale, with each point representing a county’s abundance within a season. The adult season was from October to December, with the abundance calculated as the total number of adults collected divided by the total area sampled during the season within a county

**Fig. 5:**
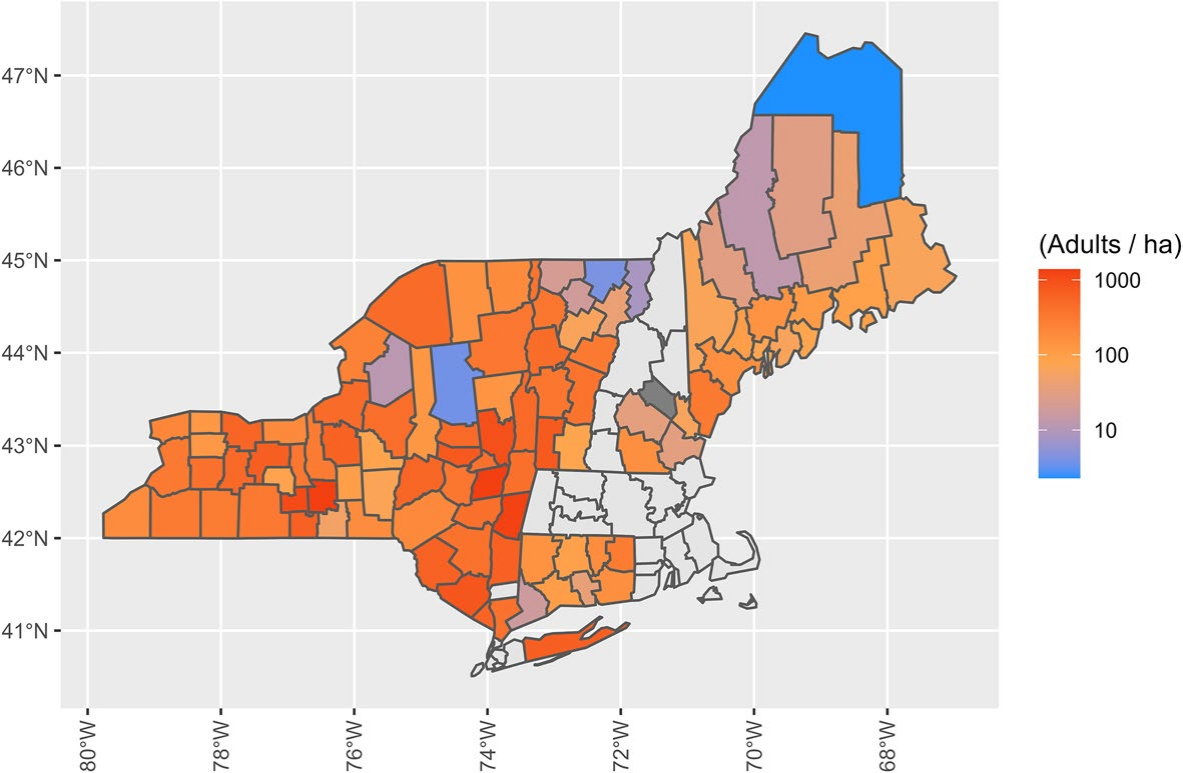
Average abundance of adult *Ixodes scapularis* per hectare in the northeastern USA. The adult season was from October to December, with the abundance calculated as the total number of adults collected divided by the total area sampled during a single season within a county. An average was taken of all abundance estimates for each county, and these are shown using a logistic scale, with dark-gray shading denoting counties that were sampled, but no adults found. Counties without color fill (light gray) were those for which we had no sampling data

The mean (± SD) adult *I. scapularis* abundance was 483.7 ± 727.0 adults per hectare for New York, 229.8 ± 444.2 adults per hectare for Vermont, 128.2 ± 127.8 adults per hectare for Connecticut, 128.1 ± 144.2 adults per hectare for Maine and 66.4 ± 99.5 adults per hectare for New Hampshire (Fig. 4). Consistent with nymph abundance, variance was large and overlapped between states. New York had the highest mean abundance of adult ticks (Fig. 5), however, Vermont, which had the lowest nymph abundance, had the second highest adult mean abundance. Evaluating our model, we found that as with nymphs, the abundance (ticks per hectare) of *I. scapularis* adults increased over time (*P* < 0.01, *t* = 8.16), with smaller adult abundance but a larger rate of abundance increase at higher latitudes (Table 2; Additional file 1: Figure S2). At our lowest latitude, the effective increase in log abundance was 0.01 (95% CI − 0.05 to 0.08) per year; at the center latitude, the effective increase was 0.08 (95% CI 0.06–0.10); and at our highest latitude, the effective increase was 0.15 (95% CI 0.09–0.21).

**Fig. 6:**
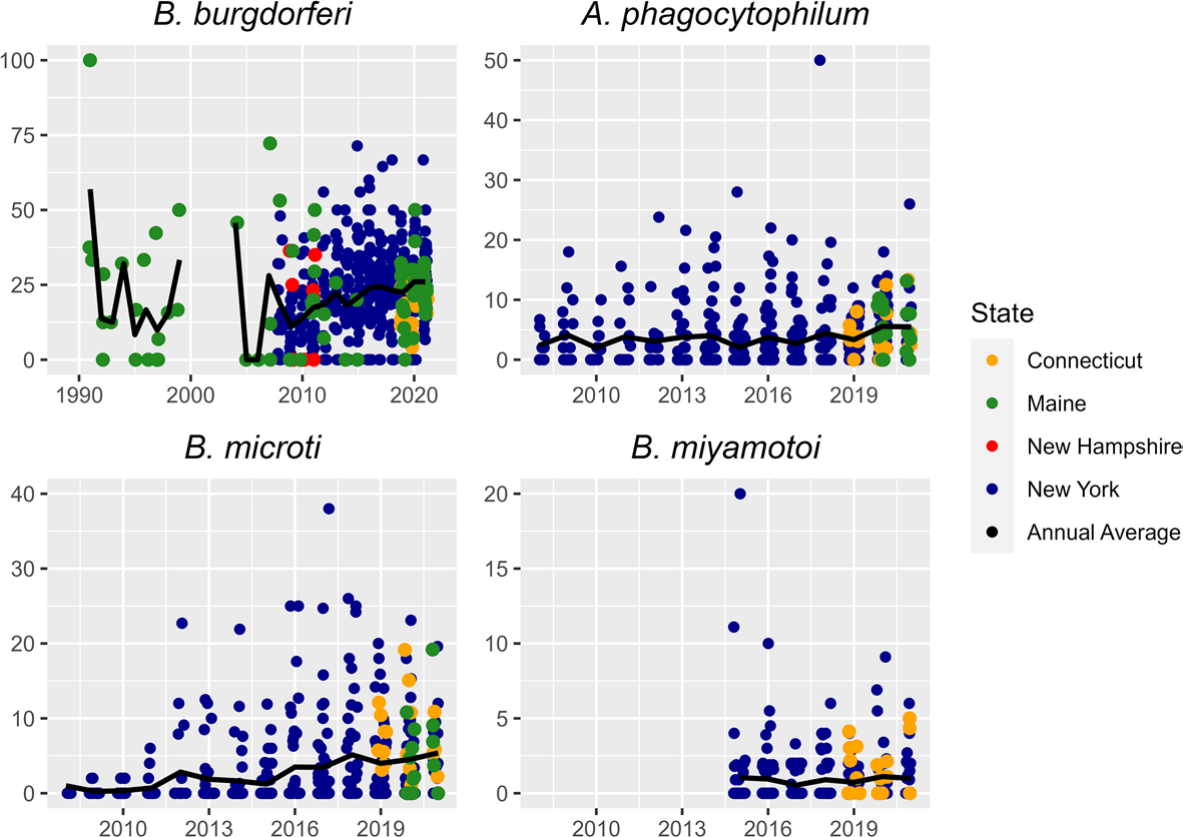
Percent pathogen prevalence of nymphal *Ixodes scapularis* in the northeastern USA, with each point representing a county’s pathogen prevalence. Pathogen prevalence was calculated as the total number of nymphs testing positive for a pathogen divided by the total number of nymphs tested for that pathogen. Top-left panel shows the prevalence of *Borrelia burgdorferi*, top-right panel shows the prevalence of *Anaplasma phagocytophilum*, bottom-left panel shows the prevalence of *Babesia microti* and bottom-right panel shows the prevalence of *Borrelia miyamotoi*

**Fig. 7:**
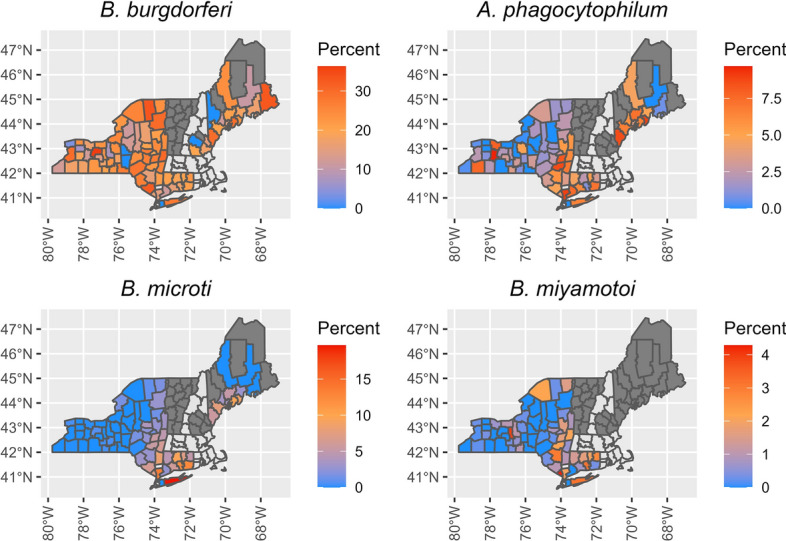
Average pathogen prevalence of nymphal *Ixodes scapularis* in the northeastern USA. Pathogen prevalence was calculated as the total number of nymphs testing positive for a pathogen divided by the total number of nymphs tested for that pathogen within a county. An average was taken of all pathogen prevalence estimates for each county, with the dark-gray shading denoting counties that were sampled for ticks, but no pathogen prevalence data were available. Counties without color fill (light gray) were those for which we had no sampling data. Top-left panel shows the prevalence of *Borrelia burgdorferi*, top-right panel shows the prevalence of *Anaplasma phagocytophilum*, bottom-left panel shows the prevalence of *Babesia microti* and bottom-right panel shows the prevalence of *Borrelia miyamotoi*

We found that *A. phagocytophilum* (*P* = 0.04, *t* = 2.06), *B. burgdorferi* (*P* < 0.01, *t* = 3.05) and *B. microti* (*P* = 0.04, *t* = 2.05) prevalence increased in nymphs over time within our models (Table 2; Fig. 6), while there was no change in *B. miyamotoi* (*P* = 0.68, *t* = − 0.42). All pathogens except for *B. burgdorferi* occurred at lower prevalence in nymphs at higher latitudes (Table 2). For *B. burgdorferi* (*P* = 0.04, *t* = − 2.08) and *B. microti* (*P* < 0.01, *t* = − 3.00), the interaction between year and latitude was negative and significant, indicating that there was less change in prevalence at higher latitudes, with no change at the highest latitudes in our study (Table 3). For *B. miyamotoi*, the mean (± SD) nymph prevalence was 1.5% ± 1.6 for Connecticut and 0.8% ± 1.9 for New York (Fig. 7). For *A. phagocytophilum*, *B. microti* and *B. burgdorferi*, we reported the mean for multiple periods, namely 2007–2011, 2012–2016 and 2017–2021, because prevalence increased over time but the data were too sparse to report annually. Among these time periods, we evaluated changes in mean values by state of pathogen prevalence, but with the large variance around means, all standard deviations overlapped. We did not identify a consistent increase in mean *B. burgdorferi* nymph prevalence across Maine, where prevalence is lower during the second time period (2012–2016) than the first (2007–2011). However, we found that mean *B. burgdorferi* nymph prevalence rose consistently in New York, the other state with data for multiple time periods (Table 4). For mean *A. phagocytophilum* and *B. microti* nymph prevalence, New York is the only state with data during multiple time periods, and we found a consistent increase in mean prevalence over time (Table 5).

**Table 3:** Effective slope (the annual change in the dependent variable taking into account the influence of latitude through the interaction term) for pathogen prevalence annual change by latitude in *Ixodes scapularis* for linear models where the interaction between year and latitude was significant

Pathogen	Latitude (decimal degrees)		
	40.75°	43.7°	46.66°
N *Borrelia burgdorferi*	1.12 (0.45–1.79)	0.38 (0.13–0.62)	− 0.37(− 1.18–0.44)
N *Babesia microti*	0.84 (0.56–1.12)	0.21 (0.01–0.42)	− 0.42 (− 1.00–0.17)
A *Anaplasma phagocytophilum*	0.23 (− 0.14–0.59)	0.92 (0.67–1.18)	1.62 (0.85–2.38)

Values in table are presented as the mean with the 95% confidence interval in parentheses

* A* Adult,* N* nymph

**Table 4:** Five-year average percent prevalence of *Borrelia burgdorferi* in *Ixodes scapularis* in the northeastern USA

State	Prevalence (%)		
	2007–2011	2012–2016	2017–2021
*Nymph*			
Connecticut	NA	NA	18.7 ± 6.6
Maine	25.0 ± 23.0	11.3 ± 10.7	24.3 ± 10.5
New Hampshire	14.0 ± 15.5	NA	NA
New York	13.9 ± 11.2	21.3 ± 14.9	25.1 ± 11.8
Vermont	NA	NA	NA
*Adult*			
Connecticut	NA	NA	49.2 ± 9.0
Maine	54.9 ± 16.4	40.3 ± 11.1	NA
New Hampshire	60.3 ± 19.0	NA	NA
New York	30.6 ± 19.6	45.2 ± 21.6	53.9 ± 12.5
Vermont	NA	37.7 ± 30.0	49.3 ± 29.3

Values in table are the 5-year average percent prevalence and are presented as the mean ± standard deviation

Testing was by PCR except for Maine where testing for *Borrelia* spp. through 2015 was by direct immunofluorescence microscopy. Pathogen prevalence was included for states with any data in the time period

*NA* Not available

**Table 5:** Five-year average percent prevalence of *Anaplasma phagocytophilum*, *Babesia microti* and *Borrelia miyamotoi* in *Ixodes scapularis* in the northeastern USA

Pathogen	State	Prevalence (%)		
		2007–2011	2012–2016	2017–2021
*Anaplasma phagocytophilum*	*Nymph*			
	Connecticut	NA	NA	4.7 ± 3.5
	Maine	NA	NA	5.5 ± 3.7
	New York	3.1 ± 4.2	3.3 ± 5.4	4.2 ± 5.6
	*Adult*			
	Connecticut	NA	NA	9.0 ± 4.8
	New York	4.4 ± 6.0	4.5 ± 6.6	8.6 ± 7.5
	Vermont	NA	4.4 ± 6.9	4.4 ± 6.2
*Babesia microti*	*Nymph*	2007–2011	2012–2016	2017–2021
	Connecticut	NA	NA	7.5 ± 5.1
	Maine	NA	NA	4.7 ± 4.8
	New York	0.6 ± 1.7	2.1 ± 4.8	4.2 ± 6.6
	*Adult*			
	Connecticut	NA	NA	12.6 ± 6.8
	New York	0.7 ± 1.9	2.2 ± 5.0	4.3 ± 5.9
	Vermont	NA	0.9 ± 2.2	2.0 ± 4.4
*Borrelia miyamotoi*	*Nymph*	2007–2011	2012–2016	2017–2021
	Connecticut	NA	NA	1.5 ± 1.6
	New York	NA	1.0 ± 2.8	0.8 ± 1.4
	*Adult*			
	Connecticut	NA	NA	2.2 ± 2.5
	New York	NA	1.1 ± 1.8	1.2 ± 1.5
	Vermont	NA	0.0 ± 0.0	0.5 ± 1.5

Values in table are the 5-year average percent prevalence and are presented as the mean ± standard deviation

Pathogen prevalence was included for states with any data in the time period

*NA* Not available

**Fig. 8:**
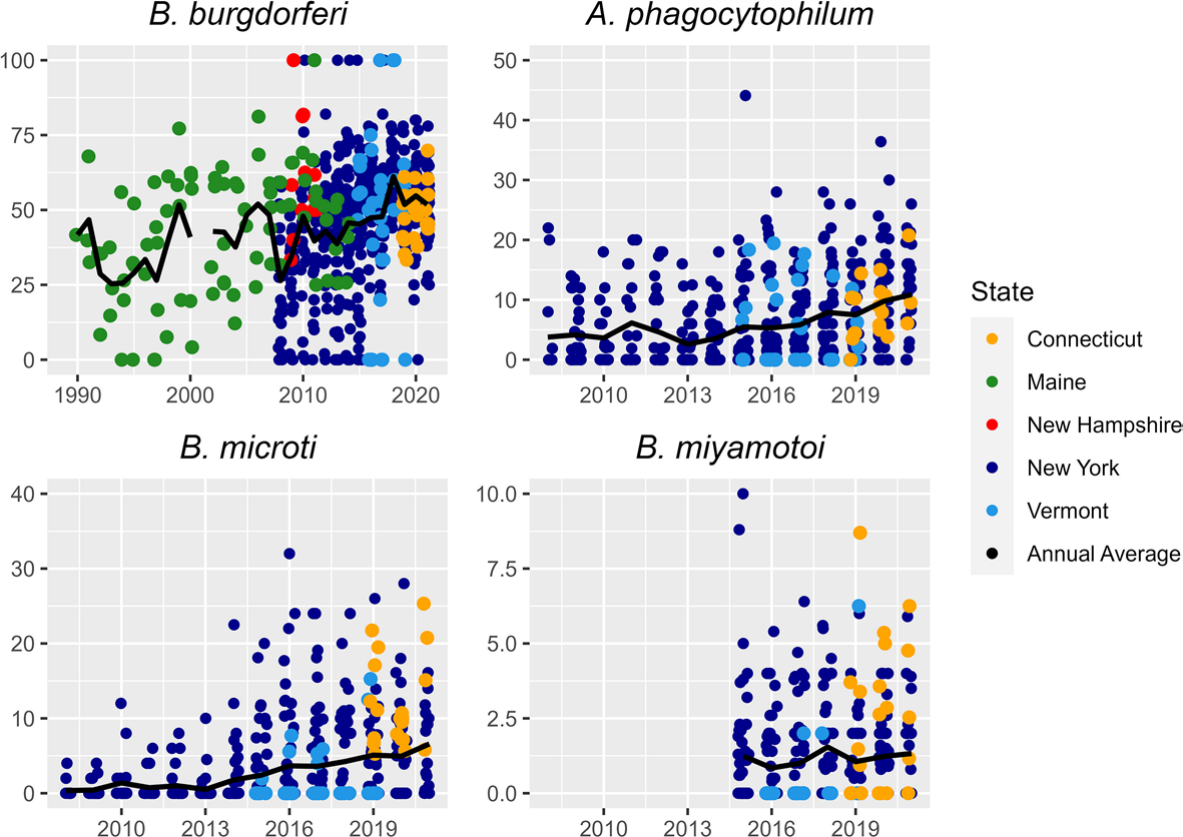
Percent pathogen prevalence of adult *Ixodes scapularis* in the northeastern USA, with each point representing a county’s pathogen prevalence. Pathogen prevalence was calculated as the total number of adults testing positive for a pathogen divided by the total number of adults tested for that pathogen. Top-left panel shows the prevalence of *Borrelia burgdorferi*, top-right panel shows the prevalence of *Anaplasma phagocytophilum*, bottom-left panel shows the prevalence of *Babesia microti* and bottom-right panel shows the prevalence of *Borrelia miyamotoi*

**Fig. 9:**
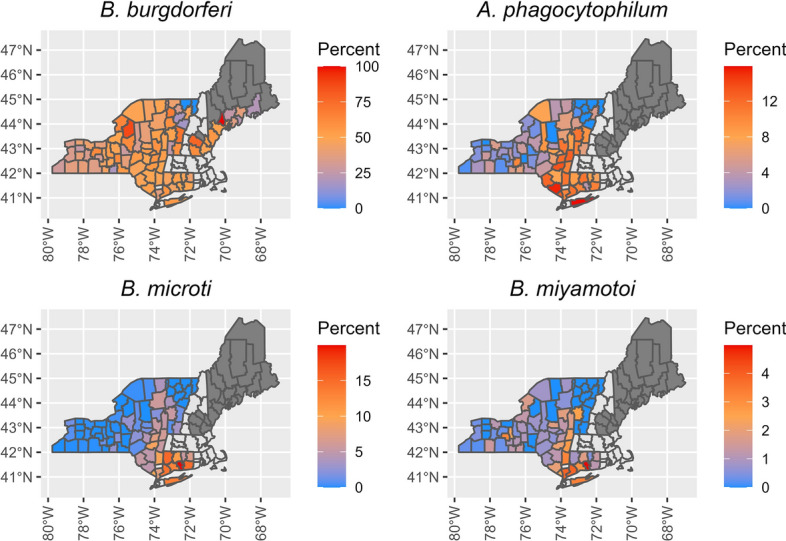
Average pathogen prevalence of adult *Ixodes scapularis* in the northeastern USA. Pathogen prevalence was calculated as the total number of nymphs testing positive for a pathogen divided by the total number of adults tested for that pathogen within a county. An average was taken of all pathogen prevalence estimates for each county, with the dark-gray shading denoting counties that were sampled for ticks, but no pathogen prevalence data were available. Counties without color fill (light gray) were those for which we had no sampling data. Top-left panel shows the prevalence of *Borrelia burgdorferi*, top-right panel shows the prevalence of *Anaplasma phagocytophilum*, bottom-left panel shows the prevalence of *Babesia microti* and bottom-right panel shows the prevalence of *Borrelia miyamotoi*

As with nymphs, we found *A. phagocytophilum* (*P* < 0.01, *t* = 7.14), *B. burgdorferi* (*P* < 0.01, *t* = 8.46) and *B. microti* (*P* < 0.01, *t* = 3.79) prevalence increased over time in adult *I. scapularis* within our models (Table 2; Fig. 8), while there was no change in *B. miyamotoi* (*P* = 0.41, *t* = 0.83). All pathogens except for *B. burgdorferi* occurred at lower prevalence in adult ticks at higher latitudes (Table 2), and the interaction between year and latitude was positive and significant for *A. phagocytophilum* (*P* = 0.01, *t* = 2.52), indicating there was more change in prevalence at higher latitudes, with no change at the lowest latitudes in our study (Table 3). For *B. miyamotoi*, the mean (± SD) adult pathogen prevalence was 2.2% ± 2.5 for Connecticut, 1.2% ± 1.6 for New York and 0.3% ± 1.2 for Vermont (Fig. 9). For *A. phagocytophilum*, *B. microti* and *B. burgdorferi* adult prevalence, we reported mean values for three time periods, 2007–2011, 2012–2016, and 2017–2021, as was done for the nymph data. Among time periods, we evaluated changes in mean values by state of pathogen prevalence; however, with the large variance around means, all standard deviations overlapped. For *B. burgdorferi* prevalence, the mean prevalence for Maine decreased from the first time period to the second time period, but we found consistent increases in adult prevalence across New York and Vermont, the only other states with data from multiple time periods (Table 4). For mean *A. phagocytophilum* and *B. microti* adult prevalence, New York and Vermont are the only states with data during multiple time periods. We found a consistent increase in mean prevalence for both pathogens in these states across time periods except for *A. phagocytophilum* in Vermont, where the mean prevalence was unchanged between the second and third time periods (Table 5).

## Discussion

In contrast with previous studies that have either collected consistent data from substantial field campaigns over limited time periods [7, 9] or classified tick distributions categorically [4–6], in the present study we leveraged disparate datasets to create a geographically extensive estimate of *I. scapularis* abundance across the northeastern USA. There is one key advantage of this approach: research on the dynamics of ticks and tick-borne disease is limited by the availability of data, and the developed dataset, when used appropriately given its constraints, can help address this limitation. Importantly, this dataset can provide a critical evaluation of process-based and statistical models of ticks and tick-borne disease [32–35] that are already being used to explore the relationships between environmental change, *I. scapularis* dynamics and tick-borne diseases. For some questions asked by modelers, using the whole dataset may be appropriate; for others, a subset of data (e.g. tick abundance for a specific set of years) may be more appropriate and is possible given the publication of source data (see Additional files).

Our results show that regional *I. scapularis* abundance was generally stable during the study period at lower latitudes, with a slight increase in abundance at higher latitudes, but that the prevalence of *A. phagocytophilum*, *B. microti* and *B. burgdorferi* increased throughout the entire region. This is consistent with the increasing number of reported anaplasmosis and babesiosis cases in the USA, although other factors (e.g. changes in availability of diagnostic testing, disease recognition by medical providers) may also explain the increased reported incidence. Nationally, anaplasmosis case counts have more than doubled, and babesiosis case counts have increased by approximately 1.4-fold from 2013 to 2019 [2]. For the five states in our study, Connecticut, Maine, New Hampshire, New York and Vermont, anaplasmosis and babesiosis case counts (Additional file 1: Table S6) have also increased based on data from the Johns Hopkins Lyme and Tickborne Diseases Dashboard [36], which spans 2008 to 2019 for anaplasmosis and from 2011 to 2019 for babesiosis. Both of these case counts show an increasing trend in these states, with anaplasmosis cases increasing tenfold during the 12-year period and babesiosis cases more than doubling during the 9-year period.

In contrast, the number of reported cases of Lyme disease during the past 10 years (from 2009 to 2019) appears to be stable across the USA [2] and in the states of interest in the present study [36], despite the increased prevalence of *B. burgdorferi* in *I. scapularis*. This could be a result of Lyme disease prevention efforts (e.g., tick avoidance, tick checks, insect repellents) or underreporting of tick-borne diseases (i.e., reported case counts are far below the Centers for Disease Control and Prevention estimated case counts), but we would expect prevention efforts and underreporting to impact case counts of anaplasmosis and babesiosis as well. When evaluating case counts by state (Additional file 1: Table S7), we noted an increase in Lyme disease in Maine and Vermont, while the case counts in Connecticut, New Hampshire and New York were stable over the last 10 years. This inconsistency between the case counts of anaplasmosis and babesiosis and the case counts of Lyme disease makes it difficult to identify potential causes, but changes in case definitions and testing protocols confound interpretation of long-term trends in the incidence of tick-borne disease [37]. Although our results indicated that *B. miyamotoi* occurs at higher prevalence at lower latitudes, we did not find an increasing prevalence of *B. miyamotoi* in *I. scapularis*. This pathogen should continue to be monitored into the future, especially since our dataset only included the prevalence of *B. miyamotoi* from 2015 to 2021, providing only a short period for us to evaluate temporal changes.

It is well documented that *I. scapularis* ticks are expanding in range [5], and as ticks enter new regions, they will increase in abundance until becoming established and reaching a stable population size. While this population size may fluctuate based on environmental conditions, these fluctuations are around an equilibrium state rather than around a trend of increasing abundance [38, 39]. Our dataset covered parts of states where tick populations are in low abundance and may still be increasing in population size, such as northern Maine. Our models show increasing abundance in northern latitudes, which indicates that southern counties already had stable populations when data collection began. The abundance of tick nymphs and adults was lower in our dataset at the most northern latitudes. We found extremely low nymph tick abundance in Vermont, including several counties with no nymphs collected, while still finding high adult tick abundance. However, this low nymph abundance is likely due to reduced sampling during the middle of the nymph tick season in Vermont (sampling occurred primarily in May with limited sampling in June, followed by resumption of sampling after September), when abundance would likely be higher. A low nymph tick abundance was also documented in New Hampshire, but sampling in New Hampshire had better coverage during times when nymphs are typically questing. Sampling in New Hampshire only occurred in the southern part of the state, so nymph abundance in northern New Hampshire remains poorly known.

While *I. scapularis* nymph abundance was low in New Hampshire, the mean number of nymphs collected is within 1 SD of the abundance reported by Diuk-Wasser et al. [8] based on tick drag surveys in 2004–2006, with the abundance determined in our study being 0.3 nymphs per hectare greater. The mean abundance for Maine (2.2 nymphs per hectare greater), New York (175 nymphs per hectare greater) and Vermont (6.9 nymphs per hectare less) in our study is also within 1 SD of that reported by Diuk-Wasser et al. [8]. However, the mean abundance for Connecticut in our study (242 nymphs per hectare less) was > 1 SD less than that reported by Diuk-Wasser et al. [8]. Diuk-Wasser et al. [8] only sampled two sites in Connecticut and Vermont, respectively, and four sites in New Hampshire, and the mean for Connecticut may be more representative of these two sites than for the state as a whole. These same authors [8] sampled 11 sites in Maine and 21 sites in New York. Our mean state abundances are lower than those identified in several other studies in the Northeast that took place in New York [40] and Vermont [41, 42], but this difference is likely due to the limited range of sampling within these earlier studies. While other studies have analyzed *I. scapularis* using active surveillance across our states of interest, we have not directly compared our results to these studies because they incorporate some of the same data that we used [43–45].

Beyond the evaluation of models, the dataset reported here could be used to ask targeted questions about the observed dynamics of tick-borne disease. One example could be to assess the association between *I. scapularis* abundance and land use, which is unlikely to substantially change over the relatively limited *I. scapularis* collection time periods. This dataset could also be applied to analyze historical pathogen prevalence in *I. scapularis*, as pathogen prevalence is normalized by the number of *I. scapularis* surveyed; therefore, it is less sensitive to differences in tick survey methods.

Despite the value of this dataset, there are several limitations that arise from compiling active tick surveillance data from multiple states over several decades. Among states, the amount of time or distance the drag was pulled or flagging was performed was completed before any check was made for ticks varied, which can affect density estimates from drag and flag surveys [46]. Converting between distance and time surveys can also introduce errors. The linear relationship that we applied to convert Maine’s surveys from time to distance had an *R*^2^ = 0.34, indicating that we did not explain a majority of the variation using this model. While this correlation is not ideal, other functional relationships, including using log and square-root transformations on these data, did not provide a better fit. Our conversion method does not account for differences in distance as calculated using time due to habitat and landscape differences, which could make movement quicker or slower while sampling ticks. Generally, the worst conversions were for very short or very long tick drags, which were rare in the dataset, and the alternative of simply excluding the data leaves out one of the most comprehensive tick survey datasets in the region. For tick drags during the nymph tick season, 97.5% of tick drags were ≥ 30 min, 75.4% of tick drags were ≥ 60 min and only 3.4% of tick drags were > 360 min. For tick drags during the adult tick season, 84.8% of tick drags were ≥ 30 min, 53.7% of tick drags were ≥ 60 min and only 2.7% of tick drags > 360 min. Additionally, some of the data for Maine were collected using flagging techniques instead of dragging, which may influence density estimates, although a difference in results between these methods for *I. scapularis* has not been identified in previous research [47, 48]. In addition, because we conducted our analysis at the county level, we disregard important sources of variability and uncertainty within counties.

In addition to differences across tick surveys that influenced *I. scapularis* abundance estimates, pathogen testing techniques varied both across states and time. Improvements in technology have increased the ability to detect pathogens in samples, and the adoption of new testing techniques may have resulted in variation in our dataset that was not caused by true changes in pathogen prevalence. These updates to techniques were not continuous and occurred at discrete times, limiting their impact on analysis, but this source of variation is still worth consideration. The *R*^2^ values for nymphal *B. burgdorferi* and *A. phagocytophilum* were < 0.1, indicating a weak model fit [49]. However, we chose to evaluate these trends because they were significant and the purpose of our linear model was to explore trends, not create an accurate model for prediction. There is also uncertainty in our findings using linear models of both abundance and pathogen prevalence data due to our combining datasets from across the northeastern USA with varying levels of coverage and data that we described in the Methods section.

The range of issues identified above highlight the need for consistent sampling protocols across space and time. Given the inconsistencies in techniques, simplistic modeling might not adequately account for the variation present. The efforts to standardize protocols by the CDC [11] can help to alleviate these problems in the future, but it is important that both agencies and academics adopt the same standard.

## Conclusions

We created an *I. scapularis* abundance and pathogen prevalence dataset by harmonizing preexisting surveillance data covering five states in northeastern USA over a 33-year time period. This is one of the most spatially and temporally comprehensive tick abundance and pathogen prevalence datasets in the USA. This effort provides a template for the construction of region-wide tick datasets which, to the extent possible, standardize disparate surveillance data collected prior to 2019, when the CDC published guidelines for standardized collections. This dataset has several important applications. First, this dataset provides a critical benchmark to evaluate both statistical and process-based models of tick abundance and pathogen prevalence, which are essential to understanding tick dynamics in a changing environment. Second, this dataset can be used together with other factors that influence tick abundance and pathogen prevalence (e.g. land cover, climate, population, forest composition, host distribution) to explore the impacts of environmental conditions on tick-borne disease. Third, this dataset could identify priorities for future surveillance, such as data collection in northern New Hampshire and Massachusetts, or more extensive surveys of areas like Vermont, which have limited nymph data in this dataset. Finally, this dataset could inform public health decisions by providing tick abundance and pathogen prevalence by county so that tick control and public outreach efforts can be focused on potential tick-borne diseases hotspots.

## Supplementary Information

**Additional file 1: Table S1.** *Ixodes scapularis* nymph abundance dataset created in the manuscript. **Table S2.** *Ixodes scapularis* adult abundance dataset created in the manuscript. **Table S3.** *Ixodes scapularis* nymph pathogen prevalence dataset created in the manuscript. **Table S4.** *Ixodes scapularis* adult pathogen prevalence dataset created in the manuscript. **Table S5.** County centroid latitudes in decimal degrees for all counties where *Ixodes scapularis* were collected. **Table S6.** Total tick-borne disease case counts across states evaluated in the manuscript summarized from data obtained from the Johns Hopkins Lyme and Tickborne Diseases Dashboard. **Table S7.** Tick-borne disease case counts by state summarized from data obtained from the Johns Hopkins Lyme and Tickborne Diseases Dashboard. **Figure S1.** *Ixodes scapularis* nymph abundance predictions by state from linear models. **Figure S2.** *Ixodes scapularis* adult abundance predictions by state from linear models.

## Data Availability

The datasets supporting the conclusions of this article are included within the article and its additional files.
