# Correction: Spatial and temporal distribution of Ixodes scapularis and tick-borne pathogens across the northeastern United States

**DOI:** 10.1186/s13071-024-06625-7

**Published:** 2025-02-21

**Authors:** Lucas E. Price, Jonathan M. Winter, Jamie L. Cantoni, Duncan W. Cozens, Megan A. Linske, Scott C. Williams, Griffin M. Dill, Allison M. Gardner, Susan P. Elias, Thomas F. Rounsville, Robert P. Smith, Michael W. Palace, Christina Herrick, Melissa A. Prusinski, Patti Casey, Eliza M. Doncaster, Joseph D. T. Savage, Dorothy I. Wallace, Xun Shi

**Affiliations:** 1 Department of Geography, Dartmouth College, 6017 Fairchild, Hanover, NH 03755 USA; 2 Department of Entomology, The Connecticut Agricultural Experiment Station, 123 Huntington Street, New Haven, CT 06511 USA; 3 Department of Environmental Science and Forestry, The Connecticut Agricultural Experiment Station, 123 Huntington Street, New Haven, CT 06511 USA; 4 Diagnostic and Research Laboratory, University of Maine Cooperative Extension, 17 Godfrey Drive, Orono, ME 04473 USA; 5 School of Biology and Ecology, University of Maine, 5722 Deering Hall, Orono, ME 04469 USA; 6 Vector-Borne Disease Laboratory, MaineHealth Institute for Research, 81 Research Drive, Scarborough, ME 04074 USA; 7 Institute for the Study of Earth, Oceans and Space, Department of Earth Sciences, University of New Hampshire, Morse Hall, Durham, NH 03824 USA; 8 Vector Ecology Laboratory, Bureau of Communicable Disease Control, New York State Department of Health, Biggs Laboratory C-456-C-470A, Wadsworth Center, Empire State Plaza, Albany, NY 12237 USA; 9 Environmental Surveillance Program, Vermont Agency of Agriculture Food & Markets, 116 State Street, Montpelier, VT 05620 USA; 10 Graduate Program in Ecology, Evolution, Environment, and Society, Dartmouth College, Hanover, NH 03755 USA; 11 Department of Mathematics, Dartmouth College, 6188 Kemeny Hall, Hanover, NH 03755 USA

**Correction: Parasites & Vectors (2024) 17:481** 10.1186/s13071-024-06518-9

Following publication of the original article [1], it came to the authors' attention that there were errors in Table 1. The column label "Borrelia microti” had been detailed instead of (the correct) “Babesia microti”, while the column label "Babesia miyamotoi” had been given instead of (the correct) “Borrelia miyamotoi”. The table has since been corrected. The authors thank you for reading this erratum and apologize for any inconvenience caused.
