# Supplementary material for: Spatial and temporal distribution of Ixodes scapularis and tick-borne pathogens across the northeastern United States
Source: Parasit Vectors. 2024 Nov 22;17:481. doi: 10.1186/s13071-024-06518-9 (PMC11583392; doi:10.1186/s13071-024-06518-9)

Table S1. Nymph *Ixodes scapularis* abundance (ticks/ha) based on active tick surveillance methods (tick dragging or flagging) in Connecticut, Maine, New Hampshire, New York, and Vermont, with tick abundance being the total number of nymphs collected within a county from May to September each year divided by the total area sampled within that time period.

| Year | State | County | Nymphs per ha |
| --- | --- | --- | --- |
| 1991 | Maine | Knox | 2.1 |
| 1991 | Maine | Lincoln | 39.3 |
| 1991 | Maine | York | 7.9 |
| 1992 | Maine | Lincoln | 16.0 |
| 1992 | Maine | York | 15.1 |
| 1993 | Maine | Knox | 0.0 |
| 1993 | Maine | Lincoln | 7.0 |
| 1993 | Maine | Sagadahoc | 0.0 |
| 1993 | Maine | York | 0.0 |
| 1994 | Maine | Cumberland | 0.0 |
| 1994 | Maine | Knox | 0.0 |
| 1994 | Maine | Sagadahoc | 0.0 |
| 1994 | Maine | Waldo | 0.0 |
| 1994 | Maine | York | 110.2 |
| 1995 | Maine | Cumberland | 3.3 |
| 1995 | Maine | Hancock | 0.0 |
| 1995 | Maine | Kennebec | 0.0 |
| 1995 | Maine | Lincoln | 32.4 |
| 1996 | Maine | Cumberland | 3.1 |
| 1996 | Maine | Knox | 26.8 |
| 1996 | Maine | Lincoln | 25.4 |
| 1996 | Maine | York | 73.5 |
| 1997 | Maine | Cumberland | 3.7 |
| 1997 | Maine | Hancock | 0.8 |
| 1997 | Maine | Lincoln | 16.1 |
| 1997 | Maine | Sagadahoc | 0.0 |
| 1997 | Maine | Waldo | 2.3 |
| 1997 | Maine | Washington | 1.8 |
| 1997 | Maine | York | 136.1 |
| 1998 | Maine | Lincoln | 10.3 |
| 1999 | Maine | Cumberland | 11.9 |
| 1999 | Maine | Lincoln | 1.4 |
| 2000 | Maine | Lincoln | 5.6 |
| 2000 | Maine | York | 130.8 |
| 2001 | Maine | Cumberland | 173.1 |
| 2001 | Maine | Lincoln | 0.0 |
| 2001 | Maine | York | 275.8 |
| 2002 | Maine | Cumberland | 141.7 |
| 2002 | Maine | Lincoln | 1.7 |
| 2002 | Maine | York | 209.3 |
| 2003 | Maine | Cumberland | 193.7 |
| 2003 | Maine | Hancock | 7.8 |
| 2003 | Maine | Lincoln | 0.0 |
| 2003 | Maine | York | 195.4 |
| 2004 | Maine | York | 293.9 |
| 2005 | Maine | Hancock | 12.4 |
| 2005 | Maine | Sagadahoc | 6.6 |
| 2006 | Maine | Sagadahoc | 19.4 |
| 2007 | Maine | Cumberland | 0.0 |
| 2007 | Maine | Kennebec | 8.0 |
| 2007 | Maine | Knox | 0.0 |
| 2007 | Maine | Oxford | 0.0 |
| 2007 | Maine | Somerset | 7.5 |
| 2007 | Maine | York | 90.2 |
| 2008 | Maine | Cumberland | 94.3 |
| 2008 | New York | Albany | 1100.0 |
| 2008 | New York | Clinton | 20.0 |
| 2008 | New York | Columbia | 1635.0 |
| 2008 | New York | Dutchess | 200.0 |
| 2008 | New York | Herkimer | 150.0 |
| 2008 | New York | Jefferson | 20.0 |
| 2008 | New York | Orange | 1200.0 |
| 2008 | New York | Oswego | 108.0 |
| 2008 | New York | Rockland | 500.0 |
| 2008 | New York | Saratoga | 148.0 |
| 2008 | New York | Schoharie | 68.0 |
| 2008 | New York | Sullivan | 150.0 |
| 2008 | New York | Ulster | 850.0 |
| 2008 | New York | Washington | 104.0 |
| 2008 | New York | Westchester | 700.0 |
| 2009 | Maine | Cumberland | 97.6 |
| 2009 | Maine | Knox | 0.0 |
| 2009 | Maine | Lincoln | 13.0 |
| 2009 | Maine | York | 4.6 |
| 2009 | New Hampshire | Hillsborough | 5.1 |
| 2009 | New Hampshire | Merrimack | 0.0 |
| 2009 | New Hampshire | Rockingham | 0.8 |
| 2009 | New Hampshire | Strafford | 3.0 |
| 2009 | New York | Albany | 445.0 |
| 2009 | New York | Cattaraugus | 21.0 |
| 2009 | New York | Chemung | 206.0 |
| 2009 | New York | Columbia | 2345.0 |
| 2009 | New York | Dutchess | 324.0 |
| 2009 | New York | Fulton | 0.0 |
| 2009 | New York | Greene | 540.0 |
| 2009 | New York | Monroe | 7.0 |
| 2009 | New York | Onondaga | 129.0 |
| 2009 | New York | Orange | 1815.0 |
| 2009 | New York | Oswego | 67.0 |
| 2009 | New York | Otsego | 8.0 |
| 2009 | New York | Rockland | 761.0 |
| 2009 | New York | Saratoga | 710.0 |
| 2009 | New York | Schuyler | 101.0 |
| 2009 | New York | Seneca | 99.0 |
| 2009 | New York | Sullivan | 483.0 |
| 2009 | New York | Ulster | 178.0 |
| 2009 | New York | Warren | 73.0 |
| 2009 | New York | Westchester | 1277.0 |
| 2010 | Maine | Cumberland | 10.2 |
| 2010 | Maine | Kennebec | 0.8 |
| 2010 | Maine | Waldo | 5.2 |
| 2010 | Maine | York | 49.0 |
| 2010 | New Hampshire | Belknap | 0.0 |
| 2010 | New Hampshire | Hillsborough | 3.7 |
| 2010 | New Hampshire | Merrimack | 4.3 |
| 2010 | New Hampshire | Rockingham | 0.0 |
| 2010 | New Hampshire | Strafford | 0.9 |
| 2010 | New York | Albany | 373.0 |
| 2010 | New York | Allegany | 0.0 |
| 2010 | New York | Cattaraugus | 71.0 |
| 2010 | New York | Chautauqua | 4.0 |
| 2010 | New York | Chemung | 84.0 |
| 2010 | New York | Columbia | 617.0 |
| 2010 | New York | Dutchess | 281.0 |
| 2010 | New York | Onondaga | 249.0 |
| 2010 | New York | Orange | 806.0 |
| 2010 | New York | Oswego | 364.0 |
| 2010 | New York | Rockland | 275.0 |
| 2010 | New York | Saratoga | 165.0 |
| 2010 | New York | Schenectady | 39.0 |
| 2010 | New York | Schuyler | 15.0 |
| 2010 | New York | Seneca | 309.0 |
| 2010 | New York | Sullivan | 223.0 |
| 2010 | New York | Ulster | 156.0 |
| 2010 | New York | Washington | 61.0 |
| 2010 | New York | Westchester | 245.0 |
| 2011 | Maine | Aroostook | 0.0 |
| 2011 | Maine | Cumberland | 6.5 |
| 2011 | Maine | Kennebec | 9.6 |
| 2011 | Maine | Knox | 7.7 |
| 2011 | Maine | Lincoln | 6.5 |
| 2011 | Maine | Penobscot | 0.0 |
| 2011 | Maine | Piscataquis | 0.0 |
| 2011 | Maine | Waldo | 55.7 |
| 2011 | Maine | Washington | 1.9 |
| 2011 | Maine | York | 23.3 |
| 2011 | New Hampshire | Belknap | 0.0 |
| 2011 | New Hampshire | Hillsborough | 12.5 |
| 2011 | New Hampshire | Merrimack | 4.5 |
| 2011 | New Hampshire | Rockingham | 15.8 |
| 2011 | New Hampshire | Strafford | 30.0 |
| 2011 | New York | Albany | 378.0 |
| 2011 | New York | Cattaraugus | 119.0 |
| 2011 | New York | Chemung | 238.0 |
| 2011 | New York | Columbia | 555.0 |
| 2011 | New York | Dutchess | 404.0 |
| 2011 | New York | Monroe | 149.0 |
| 2011 | New York | Onondaga | 193.0 |
| 2011 | New York | Orange | 3760.0 |
| 2011 | New York | Oswego | 248.0 |
| 2011 | New York | Rockland | 759.0 |
| 2011 | New York | Saratoga | 207.0 |
| 2011 | New York | Schoharie | 117.0 |
| 2011 | New York | Schuyler | 112.0 |
| 2011 | New York | Seneca | 353.0 |
| 2011 | New York | Sullivan | 580.0 |
| 2011 | New York | Ulster | 288.0 |
| 2011 | New York | Westchester | 1004.0 |
| 2012 | Maine | Aroostook | 0.0 |
| 2012 | Maine | Cumberland | 35.2 |
| 2012 | Maine | Kennebec | 21.1 |
| 2012 | Maine | Knox | 52.4 |
| 2012 | Maine | Waldo | 49.3 |
| 2012 | Maine | York | 63.1 |
| 2012 | New York | Albany | 666.0 |
| 2012 | New York | Allegany | 2.0 |
| 2012 | New York | Cattaraugus | 82.0 |
| 2012 | New York | Chautauqua | 0.0 |
| 2012 | New York | Chemung | 218.0 |
| 2012 | New York | Clinton | 45.0 |
| 2012 | New York | Columbia | 142.0 |
| 2012 | New York | Dutchess | 200.0 |
| 2012 | New York | Essex | 0.0 |
| 2012 | New York | Monroe | 376.0 |
| 2012 | New York | Onondaga | 40.0 |
| 2012 | New York | Orange | 949.0 |
| 2012 | New York | Oswego | 39.0 |
| 2012 | New York | Rockland | 91.0 |
| 2012 | New York | Saratoga | 393.0 |
| 2012 | New York | Schuyler | 82.0 |
| 2012 | New York | Seneca | 188.0 |
| 2012 | New York | Sullivan | 127.0 |
| 2012 | New York | Ulster | 147.0 |
| 2012 | New York | Warren | 98.0 |
| 2012 | New York | Westchester | 90.0 |
| 2013 | Maine | Androscoggin | 14.8 |
| 2013 | Maine | Cumberland | 41.9 |
| 2013 | Maine | Kennebec | 10.1 |
| 2013 | Maine | Knox | 27.8 |
| 2013 | Maine | Waldo | 101.8 |
| 2013 | Maine | York | 32.6 |
| 2013 | New York | Albany | 843.0 |
| 2013 | New York | Cattaraugus | 476.0 |
| 2013 | New York | Chemung | 886.0 |
| 2013 | New York | Columbia | 658.0 |
| 2013 | New York | Cortland | 39.0 |
| 2013 | New York | Dutchess | 1267.0 |
| 2013 | New York | Essex | 4.0 |
| 2013 | New York | Franklin | 23.0 |
| 2013 | New York | Fulton | 24.0 |
| 2013 | New York | Greene | 534.0 |
| 2013 | New York | Hamilton | 0.0 |
| 2013 | New York | Madison | 0.0 |
| 2013 | New York | Monroe | 1233.0 |
| 2013 | New York | Montgomery | 217.0 |
| 2013 | New York | Onondaga | 1182.0 |
| 2013 | New York | Orange | 1829.0 |
| 2013 | New York | Oswego | 567.0 |
| 2013 | New York | Otsego | 33.0 |
| 2013 | New York | Rockland | 1201.0 |
| 2013 | New York | Saratoga | 206.0 |
| 2013 | New York | Schenectady | 177.0 |
| 2013 | New York | Schoharie | 147.0 |
| 2013 | New York | Schuyler | 369.0 |
| 2013 | New York | Seneca | 473.0 |
| 2013 | New York | Suffolk | 132.0 |
| 2013 | New York | Sullivan | 543.0 |
| 2013 | New York | Ulster | 367.0 |
| 2013 | New York | Warren | 29.0 |
| 2013 | New York | Washington | 742.0 |
| 2013 | New York | Westchester | 1205.0 |
| 2014 | Maine | Cumberland | 135.1 |
| 2014 | Maine | Hancock | 44.2 |
| 2014 | Maine | York | 0.0 |
| 2014 | New York | Albany | 220.0 |
| 2014 | New York | Allegany | 0.0 |
| 2014 | New York | Broome | 0.0 |
| 2014 | New York | Cattaraugus | 71.0 |
| 2014 | New York | Chautauqua | 0.0 |
| 2014 | New York | Chemung | 667.0 |
| 2014 | New York | Chenango | 20.0 |
| 2014 | New York | Clinton | 0.0 |
| 2014 | New York | Columbia | 118.0 |
| 2014 | New York | Cortland | 0.0 |
| 2014 | New York | Delaware | 0.0 |
| 2014 | New York | Dutchess | 330.0 |
| 2014 | New York | Erie | 290.0 |
| 2014 | New York | Essex | 14.0 |
| 2014 | New York | Franklin | 0.0 |
| 2014 | New York | Fulton | 6.0 |
| 2014 | New York | Genesee | 0.0 |
| 2014 | New York | Greene | 150.0 |
| 2014 | New York | Hamilton | 0.0 |
| 2014 | New York | Herkimer | 30.0 |
| 2014 | New York | Jefferson | 0.0 |
| 2014 | New York | Lewis | 0.0 |
| 2014 | New York | Livingston | 10.0 |
| 2014 | New York | Madison | 0.0 |
| 2014 | New York | Monroe | 650.0 |
| 2014 | New York | Montgomery | 57.0 |
| 2014 | New York | Niagara | 10.0 |
| 2014 | New York | Onondaga | 16.0 |
| 2014 | New York | Ontario | 0.0 |
| 2014 | New York | Orange | 1276.0 |
| 2014 | New York | Orleans | 0.0 |
| 2014 | New York | Oswego | 85.0 |
| 2014 | New York | Otsego | 0.0 |
| 2014 | New York | Rensselaer | 41.0 |
| 2014 | New York | Rockland | 530.0 |
| 2014 | New York | Saratoga | 140.0 |
| 2014 | New York | Schenectady | 66.0 |
| 2014 | New York | Schoharie | 24.0 |
| 2014 | New York | Schuyler | 119.0 |
| 2014 | New York | Seneca | 182.0 |
| 2014 | New York | St. Lawrence | 0.0 |
| 2014 | New York | Steuben | 2.0 |
| 2014 | New York | Suffolk | 214.0 |
| 2014 | New York | Sullivan | 279.0 |
| 2014 | New York | Tompkins | 50.0 |
| 2014 | New York | Ulster | 241.0 |
| 2014 | New York | Warren | 270.0 |
| 2014 | New York | Washington | 167.0 |
| 2014 | New York | Wayne | 0.0 |
| 2014 | New York | Westchester | 925.0 |
| 2014 | New York | Wyoming | 40.0 |
| 2014 | New York | Yates | 10.0 |
| 2015 | Maine | Cumberland | 42.4 |
| 2015 | Maine | Hancock | 0.0 |
| 2015 | Maine | Knox | 14.0 |
| 2015 | Maine | Waldo | 49.3 |
| 2015 | Maine | York | 30.5 |
| 2015 | New York | Albany | 207.0 |
| 2015 | New York | Allegany | 6.0 |
| 2015 | New York | Broome | 0.0 |
| 2015 | New York | Cattaraugus | 115.0 |
| 2015 | New York | Cayuga | 58.0 |
| 2015 | New York | Chautauqua | 11.0 |
| 2015 | New York | Chemung | 186.0 |
| 2015 | New York | Clinton | 10.0 |
| 2015 | New York | Columbia | 245.0 |
| 2015 | New York | Cortland | 0.0 |
| 2015 | New York | Delaware | 155.0 |
| 2015 | New York | Dutchess | 342.0 |
| 2015 | New York | Erie | 300.0 |
| 2015 | New York | Essex | 15.0 |
| 2015 | New York | Franklin | 3.0 |
| 2015 | New York | Fulton | 10.0 |
| 2015 | New York | Genesee | 40.0 |
| 2015 | New York | Greene | 303.0 |
| 2015 | New York | Hamilton | 0.0 |
| 2015 | New York | Livingston | 40.0 |
| 2015 | New York | Monroe | 2460.0 |
| 2015 | New York | Montgomery | 108.0 |
| 2015 | New York | Nassau | 17.0 |
| 2015 | New York | Niagara | 0.0 |
| 2015 | New York | Onondaga | 90.0 |
| 2015 | New York | Orange | 1725.0 |
| 2015 | New York | Orleans | 20.0 |
| 2015 | New York | Oswego | 163.0 |
| 2015 | New York | Otsego | 119.0 |
| 2015 | New York | Rensselaer | 200.0 |
| 2015 | New York | Rockland | 486.0 |
| 2015 | New York | Saratoga | 299.0 |
| 2015 | New York | Schenectady | 40.0 |
| 2015 | New York | Schoharie | 191.0 |
| 2015 | New York | Schuyler | 157.0 |
| 2015 | New York | Seneca | 325.0 |
| 2015 | New York | St. Lawrence | 11.0 |
| 2015 | New York | Steuben | 10.0 |
| 2015 | New York | Suffolk | 78.0 |
| 2015 | New York | Sullivan | 340.0 |
| 2015 | New York | Tioga | 29.0 |
| 2015 | New York | Tompkins | 70.0 |
| 2015 | New York | Ulster | 234.0 |
| 2015 | New York | Warren | 6.0 |
| 2015 | New York | Washington | 162.0 |
| 2015 | New York | Wayne | 60.0 |
| 2015 | New York | Westchester | 557.0 |
| 2015 | New York | Wyoming | 342.0 |
| 2015 | Vermont | Bennington | 41.7 |
| 2016 | Maine | Cumberland | 43.9 |
| 2016 | Maine | Kennebec | 16.9 |
| 2016 | Maine | Knox | 25.5 |
| 2016 | Maine | Oxford | 0.0 |
| 2016 | Maine | Waldo | 54.3 |
| 2016 | Maine | York | 126.9 |
| 2016 | New York | Albany | 240.0 |
| 2016 | New York | Allegany | 25.0 |
| 2016 | New York | Broome | 40.0 |
| 2016 | New York | Cattaraugus | 59.0 |
| 2016 | New York | Cayuga | 13.0 |
| 2016 | New York | Chautauqua | 12.0 |
| 2016 | New York | Chemung | 313.0 |
| 2016 | New York | Chenango | 33.0 |
| 2016 | New York | Clinton | 0.0 |
| 2016 | New York | Columbia | 92.0 |
| 2016 | New York | Cortland | 0.0 |
| 2016 | New York | Delaware | 112.0 |
| 2016 | New York | Dutchess | 170.0 |
| 2016 | New York | Erie | 110.0 |
| 2016 | New York | Essex | 12.0 |
| 2016 | New York | Franklin | 4.0 |
| 2016 | New York | Fulton | 31.0 |
| 2016 | New York | Genesee | 0.0 |
| 2016 | New York | Greene | 113.0 |
| 2016 | New York | Hamilton | 0.0 |
| 2016 | New York | Herkimer | 0.0 |
| 2016 | New York | Jefferson | 3.0 |
| 2016 | New York | Lewis | 0.0 |
| 2016 | New York | Livingston | 0.0 |
| 2016 | New York | Madison | 0.0 |
| 2016 | New York | Monroe | 635.0 |
| 2016 | New York | Montgomery | 110.0 |
| 2016 | New York | Niagara | 10.0 |
| 2016 | New York | Oneida | 0.0 |
| 2016 | New York | Onondaga | 135.0 |
| 2016 | New York | Orange | 2596.0 |
| 2016 | New York | Oswego | 30.0 |
| 2016 | New York | Otsego | 200.0 |
| 2016 | New York | Rensselaer | 87.0 |
| 2016 | New York | Rockland | 261.0 |
| 2016 | New York | Saratoga | 197.0 |
| 2016 | New York | Schenectady | 86.0 |
| 2016 | New York | Schoharie | 79.0 |
| 2016 | New York | Schuyler | 240.0 |
| 2016 | New York | Seneca | 63.0 |
| 2016 | New York | St. Lawrence | 30.0 |
| 2016 | New York | Steuben | 0.0 |
| 2016 | New York | Suffolk | 524.0 |
| 2016 | New York | Sullivan | 305.0 |
| 2016 | New York | Tioga | 0.0 |
| 2016 | New York | Tompkins | 10.0 |
| 2016 | New York | Ulster | 66.0 |
| 2016 | New York | Warren | 69.0 |
| 2016 | New York | Washington | 190.0 |
| 2016 | New York | Westchester | 419.0 |
| 2016 | New York | Wyoming | 81.0 |
| 2016 | Vermont | Addison | 16.7 |
| 2016 | Vermont | Bennington | 0.0 |
| 2016 | Vermont | Caledonia | 0.0 |
| 2016 | Vermont | Chittenden | 0.0 |
| 2016 | Vermont | Essex | 0.0 |
| 2016 | Vermont | Franklin | 0.0 |
| 2016 | Vermont | Grand Isle | 0.0 |
| 2016 | Vermont | Lamoille | 0.0 |
| 2016 | Vermont | Orange | 0.0 |
| 2016 | Vermont | Orleans | 0.0 |
| 2016 | Vermont | Rutland | 6.3 |
| 2016 | Vermont | Washington | 0.0 |
| 2016 | Vermont | Windham | 0.0 |
| 2016 | Vermont | Windsor | 0.0 |
| 2017 | Maine | Aroostook | 0.0 |
| 2017 | Maine | Cumberland | 0.0 |
| 2017 | Maine | Hancock | 24.6 |
| 2017 | Maine | Knox | 36.2 |
| 2017 | Maine | Waldo | 12.6 |
| 2017 | New York | Albany | 501.0 |
| 2017 | New York | Allegany | 20.0 |
| 2017 | New York | Broome | 67.0 |
| 2017 | New York | Cattaraugus | 60.0 |
| 2017 | New York | Cayuga | 86.0 |
| 2017 | New York | Chautauqua | 4.0 |
| 2017 | New York | Chemung | 353.0 |
| 2017 | New York | Chenango | 0.0 |
| 2017 | New York | Clinton | 183.0 |
| 2017 | New York | Columbia | 741.0 |
| 2017 | New York | Cortland | 93.0 |
| 2017 | New York | Delaware | 145.0 |
| 2017 | New York | Dutchess | 350.0 |
| 2017 | New York | Erie | 30.0 |
| 2017 | New York | Essex | 645.0 |
| 2017 | New York | Franklin | 183.0 |
| 2017 | New York | Fulton | 27.0 |
| 2017 | New York | Genesee | 29.0 |
| 2017 | New York | Greene | 488.0 |
| 2017 | New York | Hamilton | 0.0 |
| 2017 | New York | Herkimer | 42.0 |
| 2017 | New York | Jefferson | 121.0 |
| 2017 | New York | Livingston | 56.0 |
| 2017 | New York | Madison | 0.0 |
| 2017 | New York | Monroe | 186.0 |
| 2017 | New York | Montgomery | 319.0 |
| 2017 | New York | Niagara | 90.0 |
| 2017 | New York | Oneida | 80.0 |
| 2017 | New York | Onondaga | 208.0 |
| 2017 | New York | Ontario | 304.0 |
| 2017 | New York | Orange | 1222.0 |
| 2017 | New York | Orleans | 60.0 |
| 2017 | New York | Oswego | 148.0 |
| 2017 | New York | Otsego | 208.0 |
| 2017 | New York | Rensselaer | 91.0 |
| 2017 | New York | Rockland | 813.0 |
| 2017 | New York | Saratoga | 102.0 |
| 2017 | New York | Schenectady | 286.0 |
| 2017 | New York | Schoharie | 241.0 |
| 2017 | New York | Schuyler | 349.0 |
| 2017 | New York | Seneca | 87.0 |
| 2017 | New York | St. Lawrence | 170.0 |
| 2017 | New York | Steuben | 21.0 |
| 2017 | New York | Suffolk | 354.0 |
| 2017 | New York | Sullivan | 978.0 |
| 2017 | New York | Tompkins | 47.0 |
| 2017 | New York | Ulster | 218.0 |
| 2017 | New York | Warren | 23.0 |
| 2017 | New York | Washington | 144.0 |
| 2017 | New York | Wayne | 0.0 |
| 2017 | New York | Westchester | 1776.0 |
| 2017 | New York | Wyoming | 240.0 |
| 2017 | New York | Yates | 119.0 |
| 2017 | Vermont | Addison | 93.8 |
| 2017 | Vermont | Bennington | 0.0 |
| 2017 | Vermont | Caledonia | 0.0 |
| 2017 | Vermont | Chittenden | 25.0 |
| 2017 | Vermont | Essex | 0.0 |
| 2017 | Vermont | Franklin | 0.0 |
| 2017 | Vermont | Lamoille | 0.0 |
| 2017 | Vermont | Orange | 0.0 |
| 2017 | Vermont | Orleans | 0.0 |
| 2017 | Vermont | Rutland | 0.0 |
| 2017 | Vermont | Washington | 0.0 |
| 2017 | Vermont | Windham | 6.3 |
| 2017 | Vermont | Windsor | 0.0 |
| 2018 | Maine | Cumberland | 21.4 |
| 2018 | Maine | Hancock | 27.1 |
| 2018 | Maine | Kennebec | 5.4 |
| 2018 | Maine | Knox | 14.2 |
| 2018 | Maine | Lincoln | 0.0 |
| 2018 | Maine | Waldo | 100.6 |
| 2018 | Maine | York | 125.3 |
| 2018 | New York | Albany | 554.0 |
| 2018 | New York | Allegany | 32.0 |
| 2018 | New York | Cattaraugus | 131.0 |
| 2018 | New York | Chautauqua | 20.0 |
| 2018 | New York | Chemung | 38.0 |
| 2018 | New York | Clinton | 23.0 |
| 2018 | New York | Columbia | 189.0 |
| 2018 | New York | Delaware | 138.0 |
| 2018 | New York | Dutchess | 389.0 |
| 2018 | New York | Erie | 5.0 |
| 2018 | New York | Essex | 15.0 |
| 2018 | New York | Franklin | 48.0 |
| 2018 | New York | Fulton | 9.0 |
| 2018 | New York | Greene | 291.0 |
| 2018 | New York | Hamilton | 2.0 |
| 2018 | New York | Lewis | 0.0 |
| 2018 | New York | Livingston | 7.0 |
| 2018 | New York | Monroe | 195.0 |
| 2018 | New York | Montgomery | 103.0 |
| 2018 | New York | Niagara | 5.0 |
| 2018 | New York | Onondaga | 149.0 |
| 2018 | New York | Orange | 550.0 |
| 2018 | New York | Orleans | 14.0 |
| 2018 | New York | Oswego | 70.0 |
| 2018 | New York | Otsego | 144.0 |
| 2018 | New York | Rensselaer | 38.0 |
| 2018 | New York | Rockland | 190.0 |
| 2018 | New York | Saratoga | 85.0 |
| 2018 | New York | Schenectady | 307.0 |
| 2018 | New York | Schoharie | 180.0 |
| 2018 | New York | Schuyler | 84.0 |
| 2018 | New York | St. Lawrence | 0.0 |
| 2018 | New York | Steuben | 12.0 |
| 2018 | New York | Suffolk | 746.0 |
| 2018 | New York | Sullivan | 128.0 |
| 2018 | New York | Tioga | 63.0 |
| 2018 | New York | Tompkins | 210.0 |
| 2018 | New York | Ulster | 43.0 |
| 2018 | New York | Warren | 16.0 |
| 2018 | New York | Washington | 42.0 |
| 2018 | New York | Westchester | 1786.0 |
| 2018 | New York | Wyoming | 45.0 |
| 2018 | Vermont | Addison | 0.0 |
| 2018 | Vermont | Bennington | 0.0 |
| 2018 | Vermont | Caledonia | 0.0 |
| 2018 | Vermont | Franklin | 0.0 |
| 2018 | Vermont | Orange | 0.0 |
| 2018 | Vermont | Orleans | 0.0 |
| 2018 | Vermont | Washington | 0.0 |
| 2018 | Vermont | Windham | 0.0 |
| 2018 | Vermont | Windsor | 0.0 |
| 2019 | Connecticut | Fairfield | 40.0 |
| 2019 | Connecticut | Hartford | 18.1 |
| 2019 | Connecticut | Litchfield | 60.8 |
| 2019 | Connecticut | Middlesex | 17.4 |
| 2019 | Connecticut | New Haven | 31.9 |
| 2019 | Connecticut | New London | 41.6 |
| 2019 | Connecticut | Tolland | 106.7 |
| 2019 | Connecticut | Windham | 110.9 |
| 2019 | Maine | Androscoggin | 37.0 |
| 2019 | Maine | Aroostook | 0.0 |
| 2019 | Maine | Cumberland | 104.2 |
| 2019 | Maine | Franklin | 0.0 |
| 2019 | Maine | Hancock | 178.0 |
| 2019 | Maine | Kennebec | 122.4 |
| 2019 | Maine | Knox | 82.9 |
| 2019 | Maine | Lincoln | 58.8 |
| 2019 | Maine | Oxford | 15.7 |
| 2019 | Maine | Penobscot | 2.4 |
| 2019 | Maine | Piscataquis | 0.0 |
| 2019 | Maine | Sagadahoc | 144.1 |
| 2019 | Maine | Somerset | 4.8 |
| 2019 | Maine | Waldo | 126.6 |
| 2019 | Maine | Washington | 102.1 |
| 2019 | Maine | York | 158.6 |
| 2019 | New York | Albany | 554.0 |
| 2019 | New York | Allegany | 147.5 |
| 2019 | New York | Cattaraugus | 416.8 |
| 2019 | New York | Chautauqua | 63.8 |
| 2019 | New York | Chemung | 70.0 |
| 2019 | New York | Chenango | 18.0 |
| 2019 | New York | Clinton | 87.6 |
| 2019 | New York | Columbia | 310.1 |
| 2019 | New York | Cortland | 177.0 |
| 2019 | New York | Delaware | 655.2 |
| 2019 | New York | Dutchess | 1670.0 |
| 2019 | New York | Erie | 75.4 |
| 2019 | New York | Essex | 176.5 |
| 2019 | New York | Franklin | 204.0 |
| 2019 | New York | Fulton | 99.0 |
| 2019 | New York | Genesee | 80.0 |
| 2019 | New York | Greene | 280.9 |
| 2019 | New York | Hamilton | 0.0 |
| 2019 | New York | Jefferson | 193.7 |
| 2019 | New York | Lewis | 155.0 |
| 2019 | New York | Livingston | 472.0 |
| 2019 | New York | Madison | 10.0 |
| 2019 | New York | Monroe | 907.5 |
| 2019 | New York | Montgomery | 316.3 |
| 2019 | New York | Niagara | 365.0 |
| 2019 | New York | Onondaga | 133.3 |
| 2019 | New York | Ontario | 640.0 |
| 2019 | New York | Orange | 516.5 |
| 2019 | New York | Orleans | 45.0 |
| 2019 | New York | Oswego | 87.3 |
| 2019 | New York | Otsego | 166.6 |
| 2019 | New York | Rensselaer | 165.5 |
| 2019 | New York | Rockland | 1027.0 |
| 2019 | New York | Saratoga | 206.6 |
| 2019 | New York | Schenectady | 182.6 |
| 2019 | New York | Schoharie | 249.4 |
| 2019 | New York | Schuyler | 235.0 |
| 2019 | New York | St. Lawrence | 355.0 |
| 2019 | New York | Steuben | 26.3 |
| 2019 | New York | Suffolk | 385.0 |
| 2019 | New York | Sullivan | 748.0 |
| 2019 | New York | Tompkins | 324.0 |
| 2019 | New York | Ulster | 253.5 |
| 2019 | New York | Warren | 64.2 |
| 2019 | New York | Washington | 206.8 |
| 2019 | New York | Wayne | 0.0 |
| 2019 | New York | Westchester | 1000.0 |
| 2019 | New York | Wyoming | 204.0 |
| 2020 | Connecticut | Fairfield | 15.2 |
| 2020 | Connecticut | Hartford | 29.3 |
| 2020 | Connecticut | Litchfield | 39.5 |
| 2020 | Connecticut | Middlesex | 56.5 |
| 2020 | Connecticut | New Haven | 93.3 |
| 2020 | Connecticut | New London | 124.8 |
| 2020 | Connecticut | Tolland | 125.9 |
| 2020 | Connecticut | Windham | 135.5 |
| 2020 | Maine | Androscoggin | 4.1 |
| 2020 | Maine | Cumberland | 20.9 |
| 2020 | Maine | Franklin | 0.0 |
| 2020 | Maine | Hancock | 11.1 |
| 2020 | Maine | Kennebec | 26.2 |
| 2020 | Maine | Knox | 17.4 |
| 2020 | Maine | Lincoln | 7.1 |
| 2020 | Maine | Oxford | 0.0 |
| 2020 | Maine | Penobscot | 0.0 |
| 2020 | Maine | Piscataquis | 0.0 |
| 2020 | Maine | Sagadahoc | 27.8 |
| 2020 | Maine | Somerset | 0.0 |
| 2020 | Maine | Waldo | 12.8 |
| 2020 | Maine | Washington | 4.6 |
| 2020 | Maine | York | 33.8 |
| 2020 | New York | Albany | 144.1 |
| 2020 | New York | Allegany | 69.0 |
| 2020 | New York | Cattaraugus | 245.1 |
| 2020 | New York | Cayuga | 11.5 |
| 2020 | New York | Chautauqua | 99.8 |
| 2020 | New York | Clinton | 33.6 |
| 2020 | New York | Columbia | 106.8 |
| 2020 | New York | Delaware | 128.7 |
| 2020 | New York | Dutchess | 265.7 |
| 2020 | New York | Erie | 86.0 |
| 2020 | New York | Essex | 58.3 |
| 2020 | New York | Franklin | 87.0 |
| 2020 | New York | Fulton | 22.9 |
| 2020 | New York | Genesee | 8.0 |
| 2020 | New York | Greene | 76.6 |
| 2020 | New York | Hamilton | 2.3 |
| 2020 | New York | Montgomery | 143.8 |
| 2020 | New York | Niagara | 0.0 |
| 2020 | New York | Onondaga | 231.0 |
| 2020 | New York | Oswego | 7.3 |
| 2020 | New York | Otsego | 150.3 |
| 2020 | New York | Rensselaer | 297.8 |
| 2020 | New York | Rockland | 439.5 |
| 2020 | New York | Saratoga | 54.2 |
| 2020 | New York | Schenectady | 89.3 |
| 2020 | New York | Schoharie | 86.7 |
| 2020 | New York | St. Lawrence | 60.0 |
| 2020 | New York | Steuben | 11.0 |
| 2020 | New York | Suffolk | 274.8 |
| 2020 | New York | Tompkins | 210.5 |
| 2020 | New York | Ulster | 28.3 |
| 2020 | New York | Warren | 22.0 |
| 2020 | New York | Washington | 72.2 |
| 2020 | New York | Westchester | 713.0 |
| 2021 | Connecticut | Fairfield | 95.8 |
| 2021 | Connecticut | Hartford | 53.3 |
| 2021 | Connecticut | Litchfield | 77.3 |
| 2021 | Connecticut | Middlesex | 76.2 |
| 2021 | Connecticut | New Haven | 207.1 |
| 2021 | Connecticut | New London | 109.5 |
| 2021 | Connecticut | Tolland | 127.4 |
| 2021 | Connecticut | Windham | 115.0 |
| 2021 | Maine | Androscoggin | 21.3 |
| 2021 | Maine | Cumberland | 62.9 |
| 2021 | Maine | Franklin | 5.7 |
| 2021 | Maine | Hancock | 70.6 |
| 2021 | Maine | Kennebec | 49.9 |
| 2021 | Maine | Knox | 96.6 |
| 2021 | Maine | Lincoln | 56.8 |
| 2021 | Maine | Oxford | 6.9 |
| 2021 | Maine | Penobscot | 105.6 |
| 2021 | Maine | Piscataquis | 0.0 |
| 2021 | Maine | Sagadahoc | 78.1 |
| 2021 | Maine | Somerset | 18.4 |
| 2021 | Maine | Waldo | 74.4 |
| 2021 | Maine | Washington | 28.9 |
| 2021 | Maine | York | 24.3 |
| 2021 | New York | Albany | 310.8 |
| 2021 | New York | Allegany | 630.0 |
| 2021 | New York | Cattaraugus | 500.1 |
| 2021 | New York | Chautauqua | 9.0 |
| 2021 | New York | Chemung | 314.0 |
| 2021 | New York | Clinton | 232.0 |
| 2021 | New York | Columbia | 1063.3 |
| 2021 | New York | Delaware | 434.0 |
| 2021 | New York | Dutchess | 975.0 |
| 2021 | New York | Erie | 468.3 |
| 2021 | New York | Essex | 168.0 |
| 2021 | New York | Franklin | 360.0 |
| 2021 | New York | Fulton | 96.7 |
| 2021 | New York | Genesee | 55.6 |
| 2021 | New York | Greene | 404.6 |
| 2021 | New York | Hamilton | 5.6 |
| 2021 | New York | Livingston | 147.0 |
| 2021 | New York | Madison | 25.0 |
| 2021 | New York | Monroe | 502.0 |
| 2021 | New York | Montgomery | 198.7 |
| 2021 | New York | Niagara | 85.0 |
| 2021 | New York | Oneida | 311.0 |
| 2021 | New York | Onondaga | 325.0 |
| 2021 | New York | Ontario | 1107.0 |
| 2021 | New York | Orange | 12200.0 |
| 2021 | New York | Orleans | 390.0 |
| 2021 | New York | Oswego | 229.5 |
| 2021 | New York | Otsego | 268.8 |
| 2021 | New York | Rensselaer | 859.7 |
| 2021 | New York | Rockland | 1000.0 |
| 2021 | New York | Saratoga | 204.2 |
| 2021 | New York | Schenectady | 216.7 |
| 2021 | New York | Schoharie | 356.7 |
| 2021 | New York | Schuyler | 68.0 |
| 2021 | New York | St. Lawrence | 531.0 |
| 2021 | New York | Suffolk | 539.6 |
| 2021 | New York | Sullivan | 940.0 |
| 2021 | New York | Tompkins | 673.0 |
| 2021 | New York | Ulster | 1311.0 |
| 2021 | New York | Warren | 61.0 |
| 2021 | New York | Washington | 230.8 |
| 2021 | New York | Wayne | 597.0 |
| 2021 | New York | Westchester | 1092.0 |
| 2021 | New York | Wyoming | 200.0 |
| 2021 | New York | Yates | 108.0 |
| 2022 | Maine | Cumberland | 38.5 |
| 2022 | Maine | Knox | 1.6 |
| 2022 | Maine | Waldo | 6.2 |
| 2022 | Maine | York | 71.1 |

Table S2. Adult *Ixodes scapularis* abundance (ticks/ha) based on active tick surveillance methods (tick dragging or flagging) in Connecticut, Maine, New Hampshire, New York, and Vermont, with tick abundance being the total number of adults collected within a county from October to December each year divided by the total area sampled within that time period.

| Year | State | County | Adults per ha |
| --- | --- | --- | --- |
| 1989 | Maine | York | 2.2 |
| 1990 | Maine | Lincoln | 33.5 |
| 1991 | Maine | Knox | 115.3 |
| 1991 | Maine | Lincoln | 54.1 |
| 1991 | Maine | York | 128.4 |
| 1992 | Maine | Cumberland | 20.0 |
| 1992 | Maine | Knox | 32.8 |
| 1992 | Maine | Lincoln | 33.4 |
| 1992 | Maine | York | 71.4 |
| 1993 | Maine | Knox | 21.6 |
| 1993 | Maine | Lincoln | 54.4 |
| 1993 | Maine | York | 323.1 |
| 1994 | Maine | Hancock | 0.0 |
| 1994 | Maine | Knox | 10.8 |
| 1994 | Maine | Lincoln | 68.8 |
| 1994 | Maine | York | 96.7 |
| 1995 | Maine | Cumberland | 51.8 |
| 1995 | Maine | Kennebec | 0.4 |
| 1995 | Maine | Lincoln | 51.9 |
| 1995 | Maine | Somerset | 0.0 |
| 1995 | Maine | Waldo | 0.0 |
| 1995 | Maine | York | 272.0 |
| 1996 | Maine | Cumberland | 16.2 |
| 1996 | Maine | Hancock | 1.5 |
| 1996 | Maine | Knox | 74.2 |
| 1996 | Maine | Lincoln | 35.1 |
| 1996 | Maine | Waldo | 0.0 |
| 1996 | Maine | York | 182.5 |
| 1997 | Maine | Cumberland | 0.0 |
| 1997 | Maine | Hancock | 2.5 |
| 1997 | Maine | Knox | 68.4 |
| 1997 | Maine | Lincoln | 37.6 |
| 1997 | Maine | Sagadahoc | 0.9 |
| 1997 | Maine | Waldo | 2.3 |
| 1997 | Maine | Washington | 1.4 |
| 1997 | Maine | York | 178.9 |
| 1998 | Maine | Knox | 60.9 |
| 1998 | Maine | Lincoln | 68.3 |
| 1998 | Maine | York | 256.8 |
| 1999 | Maine | Cumberland | 110.0 |
| 1999 | Maine | Knox | 59.3 |
| 1999 | Maine | Lincoln | 16.0 |
| 1999 | Maine | Washington | 0.0 |
| 1999 | Maine | York | 205.7 |
| 2000 | Maine | Cumberland | 119.0 |
| 2000 | Maine | Hancock | 14.2 |
| 2000 | Maine | Knox | 94.5 |
| 2000 | Maine | Lincoln | 37.9 |
| 2000 | Maine | York | 155.0 |
| 2001 | Maine | Cumberland | 219.4 |
| 2001 | Maine | Hancock | 18.0 |
| 2001 | Maine | Knox | 76.9 |
| 2001 | Maine | Lincoln | 40.1 |
| 2001 | Maine | York | 156.5 |
| 2002 | Maine | Cumberland | 183.2 |
| 2002 | Maine | Knox | 57.8 |
| 2002 | Maine | Lincoln | 20.7 |
| 2002 | Maine | York | 336.4 |
| 2003 | Maine | Cumberland | 95.8 |
| 2003 | Maine | Knox | 57.8 |
| 2003 | Maine | Lincoln | 4.1 |
| 2003 | Maine | Sagadahoc | 101.9 |
| 2003 | Maine | York | 356.6 |
| 2004 | Maine | Cumberland | 131.3 |
| 2004 | Maine | Knox | 74.8 |
| 2004 | Maine | Lincoln | 10.9 |
| 2004 | Maine | York | 295.0 |
| 2005 | Maine | Cumberland | 96.0 |
| 2005 | Maine | Hancock | 52.4 |
| 2005 | Maine | Lincoln | 11.3 |
| 2005 | Maine | Sagadahoc | 61.0 |
| 2005 | Maine | York | 313.6 |
| 2006 | Maine | Cumberland | 144.8 |
| 2006 | Maine | Lincoln | 1.4 |
| 2006 | Maine | Waldo | 11.1 |
| 2006 | Maine | York | 487.9 |
| 2007 | Maine | Cumberland | 110.1 |
| 2007 | Maine | Hancock | 58.0 |
| 2007 | Maine | Lincoln | 8.3 |
| 2007 | Maine | Oxford | 0.0 |
| 2007 | Maine | York | 467.9 |
| 2008 | Maine | Cumberland | 132.3 |
| 2008 | Maine | Knox | 26.1 |
| 2008 | Maine | Lincoln | 44.1 |
| 2008 | Maine | York | 377.0 |
| 2008 | New York | Albany | 2000.0 |
| 2008 | New York | Cattaraugus | 57.0 |
| 2008 | New York | Chautauqua | 5.0 |
| 2008 | New York | Chemung | 253.0 |
| 2008 | New York | Clinton | 0.0 |
| 2008 | New York | Columbia | 2300.0 |
| 2008 | New York | Dutchess | 400.0 |
| 2008 | New York | Erie | 15.0 |
| 2008 | New York | Herkimer | 20.0 |
| 2008 | New York | Jefferson | 200.0 |
| 2008 | New York | Monroe | 150.0 |
| 2008 | New York | Orange | 600.0 |
| 2008 | New York | Oswego | 255.0 |
| 2008 | New York | Rockland | 500.0 |
| 2008 | New York | Saratoga | 1200.0 |
| 2008 | New York | Schoharie | 160.0 |
| 2008 | New York | Schuyler | 8150.0 |
| 2008 | New York | Seneca | 600.0 |
| 2008 | New York | Sullivan | 366.0 |
| 2008 | New York | Tompkins | 10310.0 |
| 2008 | New York | Ulster | 500.0 |
| 2008 | New York | Washington | 150.0 |
| 2008 | New York | Westchester | 300.0 |
| 2009 | Maine | Cumberland | 94.1 |
| 2009 | Maine | Waldo | 24.3 |
| 2009 | Maine | York | 472.7 |
| 2009 | New Hampshire | Hillsborough | 8.6 |
| 2009 | New Hampshire | Merrimack | 9.4 |
| 2009 | New Hampshire | Rockingham | 19.7 |
| 2009 | New Hampshire | Strafford | 35.8 |
| 2009 | New York | Albany | 3616.0 |
| 2009 | New York | Allegany | 105.0 |
| 2009 | New York | Broome | 320.0 |
| 2009 | New York | Cattaraugus | 88.0 |
| 2009 | New York | Cayuga | 0.0 |
| 2009 | New York | Chautauqua | 1.0 |
| 2009 | New York | Chemung | 352.0 |
| 2009 | New York | Columbia | 2557.0 |
| 2009 | New York | Dutchess | 364.0 |
| 2009 | New York | Erie | 100.0 |
| 2009 | New York | Greene | 581.0 |
| 2009 | New York | Livingston | 0.0 |
| 2009 | New York | Monroe | 72.0 |
| 2009 | New York | Onondaga | 536.0 |
| 2009 | New York | Ontario | 0.0 |
| 2009 | New York | Orange | 676.0 |
| 2009 | New York | Oswego | 268.0 |
| 2009 | New York | Rensselaer | 190.0 |
| 2009 | New York | Rockland | 310.0 |
| 2009 | New York | Saratoga | 2352.0 |
| 2009 | New York | Schuyler | 135.0 |
| 2009 | New York | Seneca | 297.0 |
| 2009 | New York | Steuben | 54.0 |
| 2009 | New York | Sullivan | 685.0 |
| 2009 | New York | Ulster | 271.0 |
| 2009 | New York | Warren | 251.0 |
| 2009 | New York | Westchester | 357.0 |
| 2009 | New York | Wyoming | 0.0 |
| 2009 | New York | Yates | 8.0 |
| 2010 | Maine | Aroostook | 0.0 |
| 2010 | Maine | Cumberland | 104.1 |
| 2010 | Maine | Lincoln | 5.1 |
| 2010 | Maine | Waldo | 39.5 |
| 2010 | Maine | York | 149.6 |
| 2010 | New Hampshire | Belknap | 0.0 |
| 2010 | New Hampshire | Hillsborough | 87.5 |
| 2010 | New Hampshire | Merrimack | 25.8 |
| 2010 | New Hampshire | Rockingham | 31.5 |
| 2010 | New Hampshire | Strafford | 50.5 |
| 2010 | New York | Albany | 2217.0 |
| 2010 | New York | Allegany | 0.0 |
| 2010 | New York | Cattaraugus | 68.0 |
| 2010 | New York | Chautauqua | 0.0 |
| 2010 | New York | Chemung | 934.0 |
| 2010 | New York | Columbia | 1750.0 |
| 2010 | New York | Dutchess | 598.0 |
| 2010 | New York | Erie | 19.0 |
| 2010 | New York | Lewis | 4.0 |
| 2010 | New York | Monroe | 105.0 |
| 2010 | New York | Onondaga | 406.0 |
| 2010 | New York | Orange | 739.0 |
| 2010 | New York | Oswego | 678.0 |
| 2010 | New York | Rockland | 571.0 |
| 2010 | New York | Saratoga | 887.0 |
| 2010 | New York | Schenectady | 143.0 |
| 2010 | New York | Schuyler | 304.0 |
| 2010 | New York | Seneca | 526.0 |
| 2010 | New York | Sullivan | 395.0 |
| 2010 | New York | Ulster | 350.0 |
| 2010 | New York | Washington | 221.0 |
| 2010 | New York | Westchester | 383.0 |
| 2011 | Maine | Androscoggin | 15.0 |
| 2011 | Maine | Cumberland | 114.6 |
| 2011 | Maine | Knox | 32.2 |
| 2011 | Maine | Waldo | 111.5 |
| 2011 | Maine | York | 347.1 |
| 2011 | New Hampshire | Hillsborough | 381.8 |
| 2011 | New Hampshire | Merrimack | 65.6 |
| 2011 | New Hampshire | Rockingham | 45.4 |
| 2011 | New Hampshire | Strafford | 101.2 |
| 2011 | New York | Albany | 4267.0 |
| 2011 | New York | Allegany | 10.0 |
| 2011 | New York | Cattaraugus | 177.0 |
| 2011 | New York | Chemung | 1011.0 |
| 2011 | New York | Columbia | 1101.0 |
| 2011 | New York | Dutchess | 974.0 |
| 2011 | New York | Livingston | 57.0 |
| 2011 | New York | Monroe | 971.0 |
| 2011 | New York | Onondaga | 729.0 |
| 2011 | New York | Orange | 1271.0 |
| 2011 | New York | Oswego | 843.0 |
| 2011 | New York | Rockland | 2100.0 |
| 2011 | New York | Saratoga | 2837.0 |
| 2011 | New York | Schoharie | 133.0 |
| 2011 | New York | Schuyler | 690.0 |
| 2011 | New York | Seneca | 196.0 |
| 2011 | New York | Sullivan | 1518.0 |
| 2011 | New York | Ulster | 776.0 |
| 2011 | New York | Westchester | 1684.0 |
| 2012 | Maine | Cumberland | 118.4 |
| 2012 | Maine | Hancock | 180.6 |
| 2012 | Maine | Knox | 30.6 |
| 2012 | Maine | Waldo | 132.4 |
| 2012 | Maine | York | 96.8 |
| 2012 | New York | Albany | 854.0 |
| 2012 | New York | Allegany | 41.0 |
| 2012 | New York | Cattaraugus | 298.0 |
| 2012 | New York | Chautauqua | 61.0 |
| 2012 | New York | Chemung | 159.0 |
| 2012 | New York | Clinton | 63.0 |
| 2012 | New York | Columbia | 717.0 |
| 2012 | New York | Dutchess | 685.0 |
| 2012 | New York | Erie | 382.0 |
| 2012 | New York | Essex | 7.0 |
| 2012 | New York | Fulton | 6.0 |
| 2012 | New York | Livingston | 62.0 |
| 2012 | New York | Monroe | 492.0 |
| 2012 | New York | Montgomery | 389.0 |
| 2012 | New York | Onondaga | 398.0 |
| 2012 | New York | Orange | 478.0 |
| 2012 | New York | Oswego | 887.0 |
| 2012 | New York | Rockland | 230.0 |
| 2012 | New York | Saratoga | 989.0 |
| 2012 | New York | Schoharie | 227.0 |
| 2012 | New York | Schuyler | 272.0 |
| 2012 | New York | Seneca | 201.0 |
| 2012 | New York | Steuben | 27.0 |
| 2012 | New York | Sullivan | 388.0 |
| 2012 | New York | Ulster | 305.0 |
| 2012 | New York | Warren | 151.0 |
| 2012 | New York | Westchester | 177.0 |
| 2013 | Maine | Cumberland | 247.5 |
| 2013 | Maine | Hancock | 66.9 |
| 2013 | Maine | Knox | 14.1 |
| 2013 | Maine | Waldo | 52.1 |
| 2013 | Maine | York | 335.6 |
| 2013 | New York | Albany | 732.0 |
| 2013 | New York | Allegany | 97.0 |
| 2013 | New York | Cattaraugus | 288.0 |
| 2013 | New York | Chautauqua | 108.0 |
| 2013 | New York | Chemung | 550.0 |
| 2013 | New York | Chenango | 10.0 |
| 2013 | New York | Columbia | 753.0 |
| 2013 | New York | Cortland | 0.0 |
| 2013 | New York | Delaware | 9.0 |
| 2013 | New York | Dutchess | 403.0 |
| 2013 | New York | Erie | 156.0 |
| 2013 | New York | Essex | 0.0 |
| 2013 | New York | Franklin | 20.0 |
| 2013 | New York | Fulton | 9.0 |
| 2013 | New York | Greene | 139.0 |
| 2013 | New York | Hamilton | 4.0 |
| 2013 | New York | Jefferson | 0.0 |
| 2013 | New York | Lewis | 0.0 |
| 2013 | New York | Livingston | 65.0 |
| 2013 | New York | Madison | 10.0 |
| 2013 | New York | Monroe | 287.0 |
| 2013 | New York | Montgomery | 192.0 |
| 2013 | New York | Oneida | 0.0 |
| 2013 | New York | Onondaga | 432.0 |
| 2013 | New York | Orange | 955.0 |
| 2013 | New York | Oswego | 618.0 |
| 2013 | New York | Otsego | 18.0 |
| 2013 | New York | Rensselaer | 280.0 |
| 2013 | New York | Rockland | 424.0 |
| 2013 | New York | Saratoga | 819.0 |
| 2013 | New York | Schenectady | 529.0 |
| 2013 | New York | Schoharie | 89.0 |
| 2013 | New York | Schuyler | 233.0 |
| 2013 | New York | Seneca | 145.0 |
| 2013 | New York | Steuben | 155.0 |
| 2013 | New York | Sullivan | 216.0 |
| 2013 | New York | Tompkins | 55.0 |
| 2013 | New York | Ulster | 320.0 |
| 2013 | New York | Warren | 113.0 |
| 2013 | New York | Washington | 287.0 |
| 2013 | New York | Westchester | 213.0 |
| 2013 | New York | Wyoming | 0.0 |
| 2013 | New York | Yates | 15.0 |
| 2014 | Maine | Cumberland | 142.6 |
| 2014 | Maine | Hancock | 43.0 |
| 2014 | Maine | York | 622.2 |
| 2014 | New York | Albany | 1078.0 |
| 2014 | New York | Allegany | 112.0 |
| 2014 | New York | Broome | 339.0 |
| 2014 | New York | Cattaraugus | 221.0 |
| 2014 | New York | Chautauqua | 74.0 |
| 2014 | New York | Chemung | 215.0 |
| 2014 | New York | Chenango | 3.0 |
| 2014 | New York | Clinton | 21.0 |
| 2014 | New York | Columbia | 1523.0 |
| 2014 | New York | Cortland | 0.0 |
| 2014 | New York | Delaware | 39.0 |
| 2014 | New York | Dutchess | 494.0 |
| 2014 | New York | Erie | 190.0 |
| 2014 | New York | Essex | 194.0 |
| 2014 | New York | Franklin | 0.0 |
| 2014 | New York | Fulton | 266.0 |
| 2014 | New York | Genesee | 25.0 |
| 2014 | New York | Greene | 459.0 |
| 2014 | New York | Hamilton | 0.0 |
| 2014 | New York | Herkimer | 0.0 |
| 2014 | New York | Jefferson | 358.0 |
| 2014 | New York | Lewis | 10.0 |
| 2014 | New York | Livingston | 110.0 |
| 2014 | New York | Madison | 0.0 |
| 2014 | New York | Monroe | 525.0 |
| 2014 | New York | Montgomery | 1223.0 |
| 2014 | New York | Niagara | 0.0 |
| 2014 | New York | Oneida | 0.0 |
| 2014 | New York | Onondaga | 1173.0 |
| 2014 | New York | Ontario | 20.0 |
| 2014 | New York | Orange | 961.0 |
| 2014 | New York | Orleans | 0.0 |
| 2014 | New York | Oswego | 494.0 |
| 2014 | New York | Otsego | 161.0 |
| 2014 | New York | Rensselaer | 815.0 |
| 2014 | New York | Rockland | 545.0 |
| 2014 | New York | Saratoga | 525.0 |
| 2014 | New York | Schenectady | 208.0 |
| 2014 | New York | Schoharie | 368.0 |
| 2014 | New York | Schuyler | 120.0 |
| 2014 | New York | Seneca | 141.0 |
| 2014 | New York | Steuben | 168.0 |
| 2014 | New York | Suffolk | 409.0 |
| 2014 | New York | Sullivan | 264.0 |
| 2014 | New York | Tompkins | 90.0 |
| 2014 | New York | Ulster | 374.0 |
| 2014 | New York | Warren | 175.0 |
| 2014 | New York | Washington | 741.0 |
| 2014 | New York | Wayne | 10.0 |
| 2014 | New York | Westchester | 191.0 |
| 2014 | New York | Wyoming | 438.0 |
| 2014 | New York | Yates | 30.0 |
| 2015 | Maine | Cumberland | 286.1 |
| 2015 | Maine | Hancock | 81.7 |
| 2015 | Maine | Kennebec | 50.0 |
| 2015 | Maine | Penobscot | 12.8 |
| 2015 | Maine | Waldo | 84.3 |
| 2015 | Maine | York | 211.7 |
| 2015 | New York | Albany | 358.0 |
| 2015 | New York | Allegany | 78.0 |
| 2015 | New York | Cattaraugus | 107.0 |
| 2015 | New York | Cayuga | 0.0 |
| 2015 | New York | Chautauqua | 90.0 |
| 2015 | New York | Chemung | 203.0 |
| 2015 | New York | Chenango | 0.0 |
| 2015 | New York | Clinton | 80.0 |
| 2015 | New York | Columbia | 444.0 |
| 2015 | New York | Delaware | 102.0 |
| 2015 | New York | Dutchess | 623.0 |
| 2015 | New York | Erie | 62.0 |
| 2015 | New York | Essex | 75.0 |
| 2015 | New York | Franklin | 18.0 |
| 2015 | New York | Fulton | 330.0 |
| 2015 | New York | Genesee | 70.0 |
| 2015 | New York | Greene | 254.0 |
| 2015 | New York | Hamilton | 2.0 |
| 2015 | New York | Herkimer | 27.0 |
| 2015 | New York | Jefferson | 196.0 |
| 2015 | New York | Livingston | 903.0 |
| 2015 | New York | Madison | 2.0 |
| 2015 | New York | Monroe | 1175.0 |
| 2015 | New York | Montgomery | 1003.0 |
| 2015 | New York | Niagara | 5.0 |
| 2015 | New York | Oneida | 10.0 |
| 2015 | New York | Onondaga | 207.0 |
| 2015 | New York | Ontario | 80.0 |
| 2015 | New York | Orange | 1033.0 |
| 2015 | New York | Orleans | 30.0 |
| 2015 | New York | Oswego | 59.0 |
| 2015 | New York | Otsego | 334.0 |
| 2015 | New York | Rensselaer | 425.0 |
| 2015 | New York | Rockland | 391.0 |
| 2015 | New York | Saratoga | 464.0 |
| 2015 | New York | Schenectady | 233.0 |
| 2015 | New York | Schoharie | 230.0 |
| 2015 | New York | Schuyler | 670.0 |
| 2015 | New York | Seneca | 299.0 |
| 2015 | New York | St. Lawrence | 270.0 |
| 2015 | New York | Steuben | 101.0 |
| 2015 | New York | Suffolk | 664.0 |
| 2015 | New York | Sullivan | 230.0 |
| 2015 | New York | Tioga | 20.0 |
| 2015 | New York | Tompkins | 0.0 |
| 2015 | New York | Ulster | 294.0 |
| 2015 | New York | Warren | 59.0 |
| 2015 | New York | Washington | 530.0 |
| 2015 | New York | Wayne | 33.0 |
| 2015 | New York | Westchester | 476.0 |
| 2015 | New York | Wyoming | 247.0 |
| 2015 | New York | Yates | 69.0 |
| 2015 | Vermont | Bennington | 2133.3 |
| 2015 | Vermont | Chittenden | 781.3 |
| 2015 | Vermont | Franklin | 37.5 |
| 2015 | Vermont | Grand Isle | 750.0 |
| 2015 | Vermont | Washington | 0.0 |
| 2015 | Vermont | Windham | 125.0 |
| 2015 | Vermont | Windsor | 1587.5 |
| 2016 | Maine | Cumberland | 347.3 |
| 2016 | Maine | Hancock | 146.6 |
| 2016 | Maine | Kennebec | 138.4 |
| 2016 | Maine | Knox | 236.8 |
| 2016 | Maine | Lincoln | 277.8 |
| 2016 | Maine | Oxford | 54.8 |
| 2016 | Maine | Penobscot | 94.2 |
| 2016 | Maine | Piscataquis | 35.3 |
| 2016 | Maine | Somerset | 23.0 |
| 2016 | Maine | Waldo | 252.9 |
| 2016 | Maine | Washington | 46.7 |
| 2016 | Maine | York | 575.8 |
| 2016 | New York | Albany | 490.0 |
| 2016 | New York | Allegany | 87.0 |
| 2016 | New York | Broome | 149.0 |
| 2016 | New York | Cattaraugus | 107.0 |
| 2016 | New York | Cayuga | 459.0 |
| 2016 | New York | Chautauqua | 66.0 |
| 2016 | New York | Chemung | 158.0 |
| 2016 | New York | Chenango | 146.0 |
| 2016 | New York | Clinton | 150.0 |
| 2016 | New York | Columbia | 1141.0 |
| 2016 | New York | Cortland | 213.0 |
| 2016 | New York | Delaware | 157.0 |
| 2016 | New York | Dutchess | 631.0 |
| 2016 | New York | Erie | 471.0 |
| 2016 | New York | Essex | 1700.0 |
| 2016 | New York | Franklin | 370.0 |
| 2016 | New York | Fulton | 1526.0 |
| 2016 | New York | Genesee | 94.0 |
| 2016 | New York | Greene | 455.0 |
| 2016 | New York | Hamilton | 5.0 |
| 2016 | New York | Lewis | 33.0 |
| 2016 | New York | Livingston | 450.0 |
| 2016 | New York | Madison | 112.0 |
| 2016 | New York | Monroe | 376.0 |
| 2016 | New York | Montgomery | 2215.0 |
| 2016 | New York | Niagara | 35.0 |
| 2016 | New York | Oneida | 775.0 |
| 2016 | New York | Onondaga | 493.0 |
| 2016 | New York | Ontario | 679.0 |
| 2016 | New York | Orange | 1065.0 |
| 2016 | New York | Orleans | 160.0 |
| 2016 | New York | Oswego | 203.0 |
| 2016 | New York | Otsego | 1113.0 |
| 2016 | New York | Rensselaer | 249.0 |
| 2016 | New York | Rockland | 416.0 |
| 2016 | New York | Saratoga | 579.0 |
| 2016 | New York | Schenectady | 378.0 |
| 2016 | New York | Schoharie | 594.0 |
| 2016 | New York | Schuyler | 550.0 |
| 2016 | New York | St. Lawrence | 479.0 |
| 2016 | New York | Steuben | 215.0 |
| 2016 | New York | Suffolk | 937.0 |
| 2016 | New York | Sullivan | 380.0 |
| 2016 | New York | Tompkins | 1397.0 |
| 2016 | New York | Ulster | 251.0 |
| 2016 | New York | Warren | 69.0 |
| 2016 | New York | Washington | 669.0 |
| 2016 | New York | Westchester | 431.0 |
| 2016 | New York | Wyoming | 720.0 |
| 2016 | Vermont | Addison | 62.5 |
| 2016 | Vermont | Bennington | 8.3 |
| 2016 | Vermont | Caledonia | 0.0 |
| 2016 | Vermont | Chittenden | 175.0 |
| 2016 | Vermont | Essex | 25.0 |
| 2016 | Vermont | Franklin | 8.3 |
| 2016 | Vermont | Grand Isle | 12.5 |
| 2016 | Vermont | Lamoille | 0.0 |
| 2016 | Vermont | Orange | 16.7 |
| 2016 | Vermont | Orleans | 0.0 |
| 2016 | Vermont | Rutland | 225.0 |
| 2016 | Vermont | Washington | 25.0 |
| 2016 | Vermont | Windham | 81.3 |
| 2016 | Vermont | Windsor | 100.0 |
| 2017 | Maine | Cumberland | 246.3 |
| 2017 | Maine | Hancock | 151.7 |
| 2017 | Maine | Knox | 82.1 |
| 2017 | Maine | Lincoln | 10.8 |
| 2017 | Maine | Waldo | 267.8 |
| 2017 | Maine | Washington | 47.5 |
| 2017 | Maine | York | 1009.0 |
| 2017 | New York | Albany | 742.0 |
| 2017 | New York | Allegany | 67.0 |
| 2017 | New York | Broome | 0.0 |
| 2017 | New York | Cattaraugus | 304.0 |
| 2017 | New York | Chautauqua | 81.0 |
| 2017 | New York | Chemung | 790.0 |
| 2017 | New York | Clinton | 260.0 |
| 2017 | New York | Columbia | 1560.0 |
| 2017 | New York | Delaware | 559.0 |
| 2017 | New York | Dutchess | 442.0 |
| 2017 | New York | Erie | 313.0 |
| 2017 | New York | Essex | 206.0 |
| 2017 | New York | Franklin | 91.0 |
| 2017 | New York | Fulton | 343.0 |
| 2017 | New York | Genesee | 160.0 |
| 2017 | New York | Greene | 687.0 |
| 2017 | New York | Hamilton | 6.0 |
| 2017 | New York | Herkimer | 163.0 |
| 2017 | New York | Livingston | 549.0 |
| 2017 | New York | Madison | 100.0 |
| 2017 | New York | Monroe | 810.0 |
| 2017 | New York | Montgomery | 443.0 |
| 2017 | New York | Niagara | 200.0 |
| 2017 | New York | Onondaga | 897.0 |
| 2017 | New York | Ontario | 920.0 |
| 2017 | New York | Orange | 1306.0 |
| 2017 | New York | Orleans | 260.0 |
| 2017 | New York | Oswego | 484.0 |
| 2017 | New York | Otsego | 232.0 |
| 2017 | New York | Rensselaer | 477.0 |
| 2017 | New York | Rockland | 1183.0 |
| 2017 | New York | Saratoga | 371.0 |
| 2017 | New York | Schenectady | 364.0 |
| 2017 | New York | Schoharie | 573.0 |
| 2017 | New York | Schuyler | 910.0 |
| 2017 | New York | Seneca | 350.0 |
| 2017 | New York | St. Lawrence | 416.0 |
| 2017 | New York | Steuben | 254.0 |
| 2017 | New York | Suffolk | 1252.0 |
| 2017 | New York | Sullivan | 601.0 |
| 2017 | New York | Tioga | 13.0 |
| 2017 | New York | Tompkins | 537.0 |
| 2017 | New York | Ulster | 416.0 |
| 2017 | New York | Warren | 129.0 |
| 2017 | New York | Washington | 317.0 |
| 2017 | New York | Wayne | 0.0 |
| 2017 | New York | Westchester | 211.0 |
| 2017 | New York | Wyoming | 424.0 |
| 2017 | New York | Yates | 200.0 |
| 2017 | Vermont | Addison | 175.0 |
| 2017 | Vermont | Bennington | 125.0 |
| 2017 | Vermont | Caledonia | 118.8 |
| 2017 | Vermont | Chittenden | 62.5 |
| 2017 | Vermont | Essex | 8.3 |
| 2017 | Vermont | Franklin | 41.7 |
| 2017 | Vermont | Lamoille | 37.5 |
| 2017 | Vermont | Orange | 600.0 |
| 2017 | Vermont | Orleans | 0.0 |
| 2017 | Vermont | Rutland | 625.0 |
| 2017 | Vermont | Washington | 6.3 |
| 2017 | Vermont | Windham | 106.3 |
| 2017 | Vermont | Windsor | 220.0 |
| 2018 | Maine | Aroostook | 5.1 |
| 2018 | Maine | Cumberland | 182.7 |
| 2018 | Maine | Hancock | 290.4 |
| 2018 | Maine | Kennebec | 92.7 |
| 2018 | Maine | Knox | 171.6 |
| 2018 | Maine | Lincoln | 127.6 |
| 2018 | Maine | Sagadahoc | 121.1 |
| 2018 | Maine | Somerset | 27.4 |
| 2018 | Maine | Waldo | 192.9 |
| 2018 | Maine | York | 449.2 |
| 2018 | New York | Albany | 420.0 |
| 2018 | New York | Allegany | 193.0 |
| 2018 | New York | Cattaraugus | 624.0 |
| 2018 | New York | Chautauqua | 452.0 |
| 2018 | New York | Chemung | 380.0 |
| 2018 | New York | Chenango | 166.0 |
| 2018 | New York | Clinton | 154.0 |
| 2018 | New York | Columbia | 283.0 |
| 2018 | New York | Delaware | 155.0 |
| 2018 | New York | Dutchess | 718.0 |
| 2018 | New York | Erie | 297.0 |
| 2018 | New York | Essex | 330.0 |
| 2018 | New York | Franklin | 197.0 |
| 2018 | New York | Fulton | 899.0 |
| 2018 | New York | Greene | 273.0 |
| 2018 | New York | Jefferson | 290.0 |
| 2018 | New York | Livingston | 655.0 |
| 2018 | New York | Monroe | 329.0 |
| 2018 | New York | Montgomery | 717.0 |
| 2018 | New York | Niagara | 520.0 |
| 2018 | New York | Onondaga | 940.0 |
| 2018 | New York | Ontario | 128.0 |
| 2018 | New York | Orange | 541.0 |
| 2018 | New York | Oswego | 255.0 |
| 2018 | New York | Otsego | 404.0 |
| 2018 | New York | Rensselaer | 173.0 |
| 2018 | New York | Rockland | 407.0 |
| 2018 | New York | Saratoga | 403.0 |
| 2018 | New York | Schenectady | 129.0 |
| 2018 | New York | Schoharie | 263.0 |
| 2018 | New York | Schuyler | 290.0 |
| 2018 | New York | Seneca | 387.0 |
| 2018 | New York | St. Lawrence | 287.0 |
| 2018 | New York | Steuben | 311.0 |
| 2018 | New York | Suffolk | 520.0 |
| 2018 | New York | Sullivan | 912.0 |
| 2018 | New York | Tioga | 125.0 |
| 2018 | New York | Tompkins | 293.0 |
| 2018 | New York | Ulster | 179.0 |
| 2018 | New York | Warren | 61.0 |
| 2018 | New York | Washington | 174.0 |
| 2018 | New York | Westchester | 110.0 |
| 2018 | New York | Yates | 125.0 |
| 2018 | Vermont | Addison | 75.0 |
| 2018 | Vermont | Essex | 0.0 |
| 2018 | Vermont | Rutland | 156.3 |
| 2018 | Vermont | Windham | 8.3 |
| 2018 | Vermont | Windsor | 12.5 |
| 2019 | Connecticut | Fairfield | 8.0 |
| 2019 | Connecticut | Hartford | 24.0 |
| 2019 | Connecticut | Litchfield | 48.0 |
| 2019 | Connecticut | Middlesex | 13.3 |
| 2019 | Connecticut | New Haven | 66.7 |
| 2019 | Connecticut | New London | 18.7 |
| 2019 | Connecticut | Tolland | 64.0 |
| 2019 | Connecticut | Windham | 120.0 |
| 2019 | Maine | Androscoggin | 67.0 |
| 2019 | Maine | Cumberland | 600.4 |
| 2019 | Maine | Hancock | 157.0 |
| 2019 | Maine | Kennebec | 53.8 |
| 2019 | Maine | Knox | 141.9 |
| 2019 | Maine | Lincoln | 169.0 |
| 2019 | Maine | Oxford | 36.9 |
| 2019 | Maine | Penobscot | 24.2 |
| 2019 | Maine | Piscataquis | 65.8 |
| 2019 | Maine | Sagadahoc | 29.5 |
| 2019 | Maine | Somerset | 7.1 |
| 2019 | Maine | Waldo | 180.9 |
| 2019 | Maine | York | 356.3 |
| 2019 | New York | Albany | 1322.7 |
| 2019 | New York | Allegany | 1735.0 |
| 2019 | New York | Broome | 173.0 |
| 2019 | New York | Cattaraugus | 770.0 |
| 2019 | New York | Cayuga | 425.0 |
| 2019 | New York | Chautauqua | 588.0 |
| 2019 | New York | Clinton | 199.5 |
| 2019 | New York | Columbia | 629.8 |
| 2019 | New York | Delaware | 182.5 |
| 2019 | New York | Dutchess | 520.0 |
| 2019 | New York | Erie | 680.5 |
| 2019 | New York | Essex | 283.5 |
| 2019 | New York | Franklin | 119.4 |
| 2019 | New York | Fulton | 871.7 |
| 2019 | New York | Genesee | 248.0 |
| 2019 | New York | Greene | 436.2 |
| 2019 | New York | Hamilton | 3.2 |
| 2019 | New York | Jefferson | 368.0 |
| 2019 | New York | Livingston | 1083.0 |
| 2019 | New York | Monroe | 1152.5 |
| 2019 | New York | Montgomery | 849.7 |
| 2019 | New York | Niagara | 500.0 |
| 2019 | New York | Oneida | 1079.0 |
| 2019 | New York | Onondaga | 908.0 |
| 2019 | New York | Ontario | 1840.0 |
| 2019 | New York | Orange | 986.0 |
| 2019 | New York | Orleans | 148.0 |
| 2019 | New York | Oswego | 775.0 |
| 2019 | New York | Otsego | 1348.3 |
| 2019 | New York | Rensselaer | 539.7 |
| 2019 | New York | Rockland | 317.5 |
| 2019 | New York | Saratoga | 627.5 |
| 2019 | New York | Schenectady | 454.3 |
| 2019 | New York | Schoharie | 514.7 |
| 2019 | New York | St. Lawrence | 507.0 |
| 2019 | New York | Suffolk | 305.8 |
| 2019 | New York | Sullivan | 689.0 |
| 2019 | New York | Tompkins | 368.2 |
| 2019 | New York | Ulster | 600.0 |
| 2019 | New York | Warren | 115.4 |
| 2019 | New York | Washington | 800.3 |
| 2019 | New York | Westchester | 326.3 |
| 2019 | New York | Wyoming | 298.0 |
| 2019 | Vermont | Addison | 1475.0 |
| 2019 | Vermont | Caledonia | 0.0 |
| 2019 | Vermont | Chittenden | 383.3 |
| 2019 | Vermont | Essex | 0.0 |
| 2019 | Vermont | Franklin | 0.0 |
| 2019 | Vermont | Orleans | 12.5 |
| 2019 | Vermont | Rutland | 400.0 |
| 2019 | Vermont | Washington | 225.0 |
| 2019 | Vermont | Windsor | 0.0 |
| 2020 | Connecticut | Fairfield | 26.7 |
| 2020 | Connecticut | Hartford | 237.3 |
| 2020 | Connecticut | Litchfield | 186.7 |
| 2020 | Connecticut | Middlesex | 26.7 |
| 2020 | Connecticut | New Haven | 178.7 |
| 2020 | Connecticut | New London | 413.3 |
| 2020 | Connecticut | Tolland | 208.0 |
| 2020 | Connecticut | Windham | 424.0 |
| 2020 | Maine | Androscoggin | 72.8 |
| 2020 | Maine | Aroostook | 1.5 |
| 2020 | Maine | Cumberland | 199.8 |
| 2020 | Maine | Franklin | 22.0 |
| 2020 | Maine | Hancock | 138.0 |
| 2020 | Maine | Kennebec | 122.4 |
| 2020 | Maine | Knox | 228.6 |
| 2020 | Maine | Lincoln | 140.4 |
| 2020 | Maine | Oxford | 56.6 |
| 2020 | Maine | Piscataquis | 2.7 |
| 2020 | Maine | Sagadahoc | 108.8 |
| 2020 | Maine | Waldo | 201.1 |
| 2020 | Maine | Washington | 73.0 |
| 2020 | Maine | York | 223.2 |
| 2020 | New York | Albany | 371.5 |
| 2020 | New York | Allegany | 500.0 |
| 2020 | New York | Cattaraugus | 573.8 |
| 2020 | New York | Cayuga | 445.0 |
| 2020 | New York | Chautauqua | 402.5 |
| 2020 | New York | Chemung | 2569.0 |
| 2020 | New York | Chenango | 31.0 |
| 2020 | New York | Clinton | 320.3 |
| 2020 | New York | Columbia | 2492.8 |
| 2020 | New York | Cortland | 183.0 |
| 2020 | New York | Delaware | 219.3 |
| 2020 | New York | Dutchess | 1330.0 |
| 2020 | New York | Erie | 152.8 |
| 2020 | New York | Essex | 205.3 |
| 2020 | New York | Franklin | 187.0 |
| 2020 | New York | Fulton | 301.7 |
| 2020 | New York | Genesee | 85.0 |
| 2020 | New York | Greene | 400.0 |
| 2020 | New York | Hamilton | 2.5 |
| 2020 | New York | Jefferson | 267.0 |
| 2020 | New York | Livingston | 1537.0 |
| 2020 | New York | Monroe | 423.0 |
| 2020 | New York | Montgomery | 309.3 |
| 2020 | New York | Niagara | 220.0 |
| 2020 | New York | Oneida | 785.0 |
| 2020 | New York | Onondaga | 353.0 |
| 2020 | New York | Ontario | 1486.0 |
| 2020 | New York | Orange | 631.0 |
| 2020 | New York | Orleans | 78.0 |
| 2020 | New York | Oswego | 450.5 |
| 2020 | New York | Otsego | 149.3 |
| 2020 | New York | Rensselaer | 261.3 |
| 2020 | New York | Rockland | 464.0 |
| 2020 | New York | Saratoga | 568.0 |
| 2020 | New York | Schenectady | 639.0 |
| 2020 | New York | Schoharie | 417.7 |
| 2020 | New York | Schuyler | 753.0 |
| 2020 | New York | Seneca | 355.0 |
| 2020 | New York | St. Lawrence | 425.0 |
| 2020 | New York | Steuben | 603.5 |
| 2020 | New York | Suffolk | 517.7 |
| 2020 | New York | Sullivan | 749.0 |
| 2020 | New York | Tompkins | 524.0 |
| 2020 | New York | Ulster | 408.5 |
| 2020 | New York | Warren | 198.0 |
| 2020 | New York | Washington | 672.3 |
| 2020 | New York | Wayne | 705.0 |
| 2020 | New York | Westchester | 420.5 |
| 2020 | New York | Wyoming | 1058.0 |
| 2020 | New York | Yates | 145.0 |
| 2021 | Connecticut | Fairfield | 18.7 |
| 2021 | Connecticut | Hartford | 24.0 |
| 2021 | Connecticut | Litchfield | 194.7 |
| 2021 | Connecticut | Middlesex | 72.0 |
| 2021 | Connecticut | New Haven | 66.7 |
| 2021 | Connecticut | New London | 53.3 |
| 2021 | Connecticut | Tolland | 250.7 |
| 2021 | Connecticut | Windham | 333.3 |
| 2021 | Maine | Androscoggin | 247.4 |
| 2021 | Maine | Aroostook | 2.5 |
| 2021 | Maine | Cumberland | 221.5 |
| 2021 | Maine | Franklin | 39.3 |
| 2021 | Maine | Hancock | 212.5 |
| 2021 | Maine | Kennebec | 536.6 |
| 2021 | Maine | Knox | 86.7 |
| 2021 | Maine | Lincoln | 295.8 |
| 2021 | Maine | Oxford | 190.3 |
| 2021 | Maine | Penobscot | 37.9 |
| 2021 | Maine | Piscataquis | 13.5 |
| 2021 | Maine | Sagadahoc | 105.1 |
| 2021 | Maine | Somerset | 6.2 |
| 2021 | Maine | Waldo | 217.2 |
| 2021 | Maine | Washington | 222.3 |
| 2021 | Maine | York | 393.2 |
| 2021 | New York | Albany | 858.3 |
| 2021 | New York | Allegany | 768.0 |
| 2021 | New York | Broome | 102.0 |
| 2021 | New York | Cattaraugus | 498.4 |
| 2021 | New York | Cayuga | 453.0 |
| 2021 | New York | Chautauqua | 367.0 |
| 2021 | New York | Chenango | 142.7 |
| 2021 | New York | Clinton | 613.0 |
| 2021 | New York | Columbia | 763.3 |
| 2021 | New York | Delaware | 327.0 |
| 2021 | New York | Dutchess | 668.0 |
| 2021 | New York | Erie | 1136.0 |
| 2021 | New York | Franklin | 331.0 |
| 2021 | New York | Fulton | 336.0 |
| 2021 | New York | Genesee | 157.0 |
| 2021 | New York | Greene | 411.8 |
| 2021 | New York | Hamilton | 7.8 |
| 2021 | New York | Herkimer | 422.0 |
| 2021 | New York | Jefferson | 278.0 |
| 2021 | New York | Madison | 272.0 |
| 2021 | New York | Monroe | 896.7 |
| 2021 | New York | Montgomery | 322.8 |
| 2021 | New York | Oneida | 406.0 |
| 2021 | New York | Onondaga | 268.0 |
| 2021 | New York | Ontario | 823.0 |
| 2021 | New York | Orange | 373.0 |
| 2021 | New York | Orleans | 186.0 |
| 2021 | New York | Oswego | 489.9 |
| 2021 | New York | Otsego | 1187.0 |
| 2021 | New York | Rensselaer | 777.1 |
| 2021 | New York | Rockland | 140.5 |
| 2021 | New York | Saratoga | 806.3 |
| 2021 | New York | Schenectady | 969.3 |
| 2021 | New York | Schoharie | 629.0 |
| 2021 | New York | Schuyler | 744.0 |
| 2021 | New York | Seneca | 520.0 |
| 2021 | New York | St. Lawrence | 882.0 |
| 2021 | New York | Steuben | 1273.5 |
| 2021 | New York | Suffolk | 634.9 |
| 2021 | New York | Sullivan | 1150.0 |
| 2021 | New York | Tompkins | 186.0 |
| 2021 | New York | Ulster | 363.0 |
| 2021 | New York | Warren | 138.5 |
| 2021 | New York | Washington | 891.0 |
| 2021 | New York | Westchester | 123.3 |

Table S3. Nymph *Ixodes scapularis* percent pathogen prevalence from ticks collected using active tick surveillance methods (tick dragging or flagging) in Connecticut, Maine, New Hampshire, New York, and Vermont.

| Year | State | County | *B. burgdorferi* | *A. phagocytophilum* | *B. microti* | *B. miyamotoi* |
| --- | --- | --- | --- | --- | --- | --- |
| 1991 | Maine | Knox | 33.3 | NA | NA | NA |
| 1991 | Maine | Lincoln | 100.0 | NA | NA | NA |
| 1991 | Maine | York | 37.5 | NA | NA | NA |
| 1992 | Maine | Cumberland | 0.0 | NA | NA | NA |
| 1992 | Maine | Lincoln | 28.6 | NA | NA | NA |
| 1992 | Maine | York | 12.5 | NA | NA | NA |
| 1993 | Maine | York | 12.5 | NA | NA | NA |
| 1994 | Maine | York | 32.1 | NA | NA | NA |
| 1995 | Maine | Cumberland | 0.0 | NA | NA | NA |
| 1995 | Maine | Lincoln | 16.7 | NA | NA | NA |
| 1996 | Maine | Cumberland | 33.3 | NA | NA | NA |
| 1996 | Maine | Lincoln | 0.0 | NA | NA | NA |
| 1997 | Maine | Cumberland | 0.0 | NA | NA | NA |
| 1997 | Maine | Hancock | 0.0 | NA | NA | NA |
| 1997 | Maine | Lincoln | 6.9 | NA | NA | NA |
| 1997 | Maine | Waldo | 0.0 | NA | NA | NA |
| 1997 | Maine | York | 42.3 | NA | NA | NA |
| 1998 | Maine | Lincoln | 15.7 | NA | NA | NA |
| 1999 | Maine | Cumberland | 16.7 | NA | NA | NA |
| 1999 | Maine | Lincoln | 50.0 | NA | NA | NA |
| 2004 | Maine | York | 45.7 | NA | NA | NA |
| 2005 | Maine | Sagadahoc | 0.0 | NA | NA | NA |
| 2006 | Maine | Sagadahoc | 0.0 | NA | NA | NA |
| 2007 | Maine | Kennebec | 12.0 | NA | NA | NA |
| 2007 | Maine | Somerset | 0.0 | NA | NA | NA |
| 2007 | Maine | York | 72.2 | NA | NA | NA |
| 2008 | Maine | Cumberland | 53.1 | NA | NA | NA |
| 2008 | New York | Albany | 40.0 | 0.0 | 0.0 | NA |
| 2008 | New York | Clinton | 0.0 | 0.0 | 0.0 | NA |
| 2008 | New York | Columbia | 48.0 | 6.0 | 0.0 | NA |
| 2008 | New York | Dutchess | 32.0 | 4.0 | 8.0 | NA |
| 2008 | New York | Herkimer | 0.0 | 0.0 | 0.0 | NA |
| 2008 | New York | Jefferson | 0.0 | 0.0 | 0.0 | NA |
| 2008 | New York | Onondaga | 32.1 | 1.9 | 0.0 | NA |
| 2008 | New York | Orange | 14.0 | 2.0 | 0.0 | NA |
| 2008 | New York | Oswego | 14.3 | 2.0 | 0.0 | NA |
| 2008 | New York | Rockland | 10.0 | 2.0 | 0.0 | NA |
| 2008 | New York | Saratoga | 23.3 | 6.7 | 0.0 | NA |
| 2008 | New York | Schoharie | 2.1 | 0.0 | 0.0 | NA |
| 2008 | New York | Sullivan | 12.5 | 0.0 | 0.0 | NA |
| 2008 | New York | Ulster | 16.0 | 2.0 | 0.0 | NA |
| 2008 | New York | Washington | 10.9 | 5.5 | 0.0 | NA |
| 2008 | New York | Westchester | 10.0 | 6.0 | 8.0 | NA |
| 2009 | Maine | Cumberland | 36.4 | NA | NA | NA |
| 2009 | Maine | Lincoln | 0.0 | NA | NA | NA |
| 2009 | Maine | York | 0.0 | NA | NA | NA |
| 2009 | New Hampshire | Hillsborough | 36.4 | NA | NA | NA |
| 2009 | New Hampshire | Rockingham | 0.0 | NA | NA | NA |
| 2009 | New Hampshire | Strafford | 25.0 | NA | NA | NA |
| 2009 | New York | Albany | 20.0 | 2.0 | 0.0 | NA |
| 2009 | New York | Cattaraugus | 0.0 | 0.0 | 0.0 | NA |
| 2009 | New York | Chemung | 0.0 | 0.0 | 0.0 | NA |
| 2009 | New York | Columbia | 14.3 | 4.1 | 0.0 | NA |
| 2009 | New York | Dutchess | 2.0 | 2.0 | 0.0 | NA |
| 2009 | New York | Greene | 8.0 | 0.0 | 0.0 | NA |
| 2009 | New York | Orange | 6.0 | 6.0 | 0.0 | NA |
| 2009 | New York | Oswego | 20.0 | 0.0 | 0.0 | NA |
| 2009 | New York | Otsego | 0.0 | 0.0 | 0.0 | NA |
| 2009 | New York | Rockland | 2.0 | 10.0 | 0.0 | NA |
| 2009 | New York | Saratoga | 6.0 | 6.0 | 0.0 | NA |
| 2009 | New York | Schuyler | 11.8 | 0.0 | 0.0 | NA |
| 2009 | New York | Seneca | 14.0 | 8.0 | 0.0 | NA |
| 2009 | New York | Sullivan | 8.0 | 2.0 | 0.0 | NA |
| 2009 | New York | Ulster | 22.0 | 18.0 | 2.0 | NA |
| 2009 | New York | Warren | 17.1 | 0.0 | 0.0 | NA |
| 2009 | New York | Westchester | 4.0 | 12.0 | 2.0 | NA |
| 2010 | Maine | Waldo | 0.0 | NA | NA | NA |
| 2010 | New Hampshire | Hillsborough | 0.0 | NA | NA | NA |
| 2010 | New Hampshire | Merrimack | 0.0 | NA | NA | NA |
| 2010 | New Hampshire | Strafford | 0.0 | NA | NA | NA |
| 2010 | New York | Albany | 36.2 | 6.4 | 0.0 | NA |
| 2010 | New York | Cattaraugus | 21.6 | 0.0 | 0.0 | NA |
| 2010 | New York | Chautauqua | 0.0 | 0.0 | 0.0 | NA |
| 2010 | New York | Chemung | 14.0 | 2.0 | 0.0 | NA |
| 2010 | New York | Columbia | 18.0 | 10.0 | 2.0 | NA |
| 2010 | New York | Dutchess | 12.0 | 0.0 | 0.0 | NA |
| 2010 | New York | Onondaga | 35.2 | 1.9 | 0.0 | NA |
| 2010 | New York | Orange | 6.0 | 2.0 | 0.0 | NA |
| 2010 | New York | Oswego | 28.6 | 0.0 | 0.0 | NA |
| 2010 | New York | Rockland | 8.0 | 4.0 | 2.0 | NA |
| 2010 | New York | Saratoga | 40.7 | 0.0 | 0.0 | NA |
| 2010 | New York | Schenectady | 11.1 | 0.0 | 0.0 | NA |
| 2010 | New York | Schuyler | 0.0 | 0.0 | 0.0 | NA |
| 2010 | New York | Seneca | 23.3 | 1.7 | 0.0 | NA |
| 2010 | New York | Sullivan | 22.0 | 0.0 | 0.0 | NA |
| 2010 | New York | Ulster | 6.0 | 4.0 | 0.0 | NA |
| 2010 | New York | Washington | 13.5 | 1.9 | 0.0 | NA |
| 2010 | New York | Westchester | 10.0 | 2.0 | 2.0 | NA |
| 2011 | Maine | Cumberland | 41.7 | NA | NA | NA |
| 2011 | Maine | Kennebec | 15.7 | NA | NA | NA |
| 2011 | Maine | Knox | 20.0 | NA | NA | NA |
| 2011 | Maine | Lincoln | 50.0 | NA | NA | NA |
| 2011 | Maine | Waldo | 29.5 | NA | NA | NA |
| 2011 | Maine | York | 18.9 | NA | NA | NA |
| 2011 | New Hampshire | Hillsborough | 23.3 | NA | NA | NA |
| 2011 | New Hampshire | Merrimack | 0.0 | NA | NA | NA |
| 2011 | New Hampshire | Rockingham | 35.0 | NA | NA | NA |
| 2011 | New Hampshire | Strafford | 20.8 | NA | NA | NA |
| 2011 | New York | Albany | 18.0 | 12.0 | 2.0 | NA |
| 2011 | New York | Cattaraugus | 7.8 | 15.6 | 0.0 | NA |
| 2011 | New York | Chemung | 11.5 | 0.0 | 0.0 | NA |
| 2011 | New York | Columbia | 25.0 | 2.3 | 0.0 | NA |
| 2011 | New York | Dutchess | 10.0 | 0.0 | 0.0 | NA |
| 2011 | New York | Monroe | 7.6 | 6.1 | 0.0 | NA |
| 2011 | New York | Onondaga | 16.1 | 3.2 | 0.0 | NA |
| 2011 | New York | Orange | 28.0 | 0.0 | 0.0 | NA |
| 2011 | New York | Oswego | 8.6 | 0.0 | 0.0 | NA |
| 2011 | New York | Rockland | 20.0 | 10.0 | 4.0 | NA |
| 2011 | New York | Saratoga | 16.7 | 0.0 | 0.0 | NA |
| 2011 | New York | Schoharie | 4.0 | 0.0 | 0.0 | NA |
| 2011 | New York | Schuyler | 3.8 | 0.0 | 0.0 | NA |
| 2011 | New York | Seneca | 20.8 | 0.0 | 0.0 | NA |
| 2011 | New York | Sullivan | 10.0 | 0.0 | 0.0 | NA |
| 2011 | New York | Ulster | 2.0 | 12.0 | 0.0 | NA |
| 2011 | New York | Westchester | 6.0 | 4.0 | 6.0 | NA |
| 2012 | Maine | Cumberland | 7.1 | NA | NA | NA |
| 2012 | Maine | Waldo | 15.2 | NA | NA | NA |
| 2012 | New York | Albany | 28.1 | 12.2 | 7.9 | NA |
| 2012 | New York | Allegany | 0.0 | 0.0 | 0.0 | NA |
| 2012 | New York | Cattaraugus | 15.0 | 23.8 | 0.0 | NA |
| 2012 | New York | Chemung | 21.7 | 3.3 | 0.0 | NA |
| 2012 | New York | Clinton | 0.0 | 0.0 | 0.0 | NA |
| 2012 | New York | Columbia | 40.0 | 4.4 | 0.0 | NA |
| 2012 | New York | Dutchess | 24.0 | 0.0 | 12.0 | NA |
| 2012 | New York | Monroe | 17.0 | 2.3 | 0.0 | NA |
| 2012 | New York | Onondaga | 0.0 | 0.0 | 0.0 | NA |
| 2012 | New York | Orange | 56.0 | 4.0 | 0.0 | NA |
| 2012 | New York | Oswego | 0.0 | 0.0 | 0.0 | NA |
| 2012 | New York | Rockland | 18.2 | 0.0 | 9.1 | NA |
| 2012 | New York | Saratoga | 15.7 | 2.0 | 0.0 | NA |
| 2012 | New York | Schuyler | 8.0 | 0.0 | 0.0 | NA |
| 2012 | New York | Seneca | 28.6 | 2.0 | 0.0 | NA |
| 2012 | New York | Sullivan | 38.0 | 2.0 | 0.0 | NA |
| 2012 | New York | Ulster | 4.0 | 0.0 | 2.0 | NA |
| 2012 | New York | Warren | 25.8 | 0.0 | 0.0 | NA |
| 2012 | New York | Westchester | 27.3 | 2.3 | 22.7 | NA |
| 2013 | Maine | Waldo | 25.7 | NA | NA | NA |
| 2013 | New York | Albany | 25.4 | 15.3 | 8.5 | NA |
| 2013 | New York | Cattaraugus | 10.8 | 21.6 | 0.0 | NA |
| 2013 | New York | Chemung | 20.0 | 2.0 | 0.0 | NA |
| 2013 | New York | Columbia | 22.6 | 4.8 | 0.0 | NA |
| 2013 | New York | Cortland | 0.0 | 0.0 | 0.0 | NA |
| 2013 | New York | Dutchess | 26.0 | 4.0 | 10.0 | NA |
| 2013 | New York | Essex | 0.0 | 0.0 | 0.0 | NA |
| 2013 | New York | Franklin | 50.0 | 0.0 | 0.0 | NA |
| 2013 | New York | Fulton | 16.7 | 0.0 | 0.0 | NA |
| 2013 | New York | Greene | 27.9 | 11.5 | 3.3 | NA |
| 2013 | New York | Monroe | 10.0 | 10.0 | 0.0 | NA |
| 2013 | New York | Montgomery | 34.8 | 0.0 | 1.1 | NA |
| 2013 | New York | Onondaga | 27.1 | 2.1 | 0.0 | NA |
| 2013 | New York | Orange | 42.0 | 6.0 | 0.0 | NA |
| 2013 | New York | Oswego | 28.9 | 0.0 | 0.0 | NA |
| 2013 | New York | Otsego | 0.0 | 0.0 | 0.0 | NA |
| 2013 | New York | Rockland | 16.0 | 0.0 | 2.0 | NA |
| 2013 | New York | Saratoga | 37.0 | 11.1 | 0.0 | NA |
| 2013 | New York | Schenectady | 16.3 | 0.0 | 0.0 | NA |
| 2013 | New York | Schoharie | 26.0 | 0.0 | 0.0 | NA |
| 2013 | New York | Schuyler | 20.0 | 0.0 | 0.0 | NA |
| 2013 | New York | Seneca | 28.0 | 0.0 | 0.0 | NA |
| 2013 | New York | Suffolk | 25.0 | 7.5 | 12.5 | NA |
| 2013 | New York | Sullivan | 26.0 | 2.0 | 0.0 | NA |
| 2013 | New York | Ulster | 18.3 | 1.4 | 2.8 | NA |
| 2013 | New York | Warren | 10.0 | 0.0 | 0.0 | NA |
| 2013 | New York | Washington | 39.7 | 0.0 | 0.0 | NA |
| 2013 | New York | Westchester | 18.0 | 6.0 | 12.0 | NA |
| 2014 | Maine | Cumberland | 0.0 | NA | NA | NA |
| 2014 | New York | Albany | 35.6 | 15.3 | 7.6 | NA |
| 2014 | New York | Cattaraugus | 14.3 | 5.4 | 0.0 | NA |
| 2014 | New York | Chemung | 24.0 | 2.0 | 0.0 | NA |
| 2014 | New York | Chenango | 0.0 | 0.0 | 0.0 | NA |
| 2014 | New York | Clinton | 0.0 | 0.0 | 0.0 | NA |
| 2014 | New York | Columbia | 32.9 | 7.1 | 3.5 | NA |
| 2014 | New York | Dutchess | 14.0 | 2.3 | 11.6 | NA |
| 2014 | New York | Erie | 14.0 | 4.0 | 0.0 | NA |
| 2014 | New York | Essex | 25.6 | 0.0 | 0.0 | NA |
| 2014 | New York | Franklin | 0.0 | 0.0 | 0.0 | NA |
| 2014 | New York | Fulton | 33.3 | 0.0 | 0.0 | NA |
| 2014 | New York | Greene | 14.8 | 20.5 | 1.1 | NA |
| 2014 | New York | Herkimer | 17.4 | 4.3 | 0.0 | NA |
| 2014 | New York | Livingston | 0.0 | 0.0 | 0.0 | NA |
| 2014 | New York | Monroe | 19.8 | 15.6 | 0.0 | NA |
| 2014 | New York | Montgomery | 46.2 | 1.9 | 0.0 | NA |
| 2014 | New York | Niagara | 0.0 | 0.0 | 0.0 | NA |
| 2014 | New York | Onondaga | 26.9 | 7.7 | 0.0 | NA |
| 2014 | New York | Orange | 24.0 | 2.0 | 0.0 | NA |
| 2014 | New York | Oswego | 33.3 | 0.0 | 0.0 | NA |
| 2014 | New York | Rensselaer | 15.0 | 0.0 | 0.0 | NA |
| 2014 | New York | Rockland | 12.0 | 0.0 | 0.0 | NA |
| 2014 | New York | Saratoga | 20.0 | 5.7 | 0.0 | NA |
| 2014 | New York | Schenectady | 31.6 | 2.6 | 0.0 | NA |
| 2014 | New York | Schoharie | 33.3 | 0.0 | 0.0 | NA |
| 2014 | New York | Schuyler | 13.7 | 0.0 | 0.0 | NA |
| 2014 | New York | Seneca | 15.7 | 9.8 | 0.0 | NA |
| 2014 | New York | Steuben | 50.0 | 0.0 | 0.0 | NA |
| 2014 | New York | Suffolk | 34.4 | 0.0 | 21.9 | NA |
| 2014 | New York | Sullivan | 20.3 | 4.1 | 0.0 | NA |
| 2014 | New York | Tompkins | 0.0 | 0.0 | 0.0 | NA |
| 2014 | New York | Ulster | 17.0 | 5.7 | 5.7 | NA |
| 2014 | New York | Warren | 26.0 | 1.0 | 0.0 | NA |
| 2014 | New York | Washington | 17.3 | 18.7 | 1.3 | NA |
| 2014 | New York | Westchester | 6.1 | 12.2 | 8.2 | NA |
| 2014 | New York | Wyoming | 0.0 | 0.0 | 0.0 | NA |
| 2014 | New York | Yates | 0.0 | 0.0 | 0.0 | NA |
| 2015 | Maine | Knox | 0.0 | NA | NA | NA |
| 2015 | Maine | Waldo | 20.0 | NA | NA | NA |
| 2015 | New York | Albany | 17.8 | 6.8 | 4.1 | 0.0 |
| 2015 | New York | Allegany | 0.0 | 0.0 | 0.0 | 0.0 |
| 2015 | New York | Broome | 0.0 | 0.0 | 0.0 | 0.0 |
| 2015 | New York | Cattaraugus | 11.8 | 2.9 | 0.0 | 0.0 |
| 2015 | New York | Cayuga | 8.9 | 0.0 | 0.0 | 2.2 |
| 2015 | New York | Chautauqua | 0.0 | 0.0 | 0.0 | 0.0 |
| 2015 | New York | Chemung | 13.7 | 0.0 | 0.0 | 0.0 |
| 2015 | New York | Clinton | 56.0 | 4.0 | 0.0 | 4.0 |
| 2015 | New York | Columbia | 29.6 | 5.6 | 8.5 | 1.4 |
| 2015 | New York | Delaware | 15.3 | 0.0 | 0.0 | 0.0 |
| 2015 | New York | Dutchess | 34.8 | 1.8 | 2.7 | 1.8 |
| 2015 | New York | Erie | 33.3 | 0.0 | 0.0 | 0.0 |
| 2015 | New York | Essex | 41.4 | 0.0 | 0.0 | 0.0 |
| 2015 | New York | Franklin | 71.4 | 0.0 | 0.0 | 0.0 |
| 2015 | New York | Fulton | 30.0 | 0.0 | 0.0 | 0.0 |
| 2015 | New York | Genesee | 0.0 | 0.0 | 0.0 | 0.0 |
| 2015 | New York | Greene | 26.7 | 1.7 | 1.7 | 0.0 |
| 2015 | New York | Hamilton | 0.0 | 0.0 | 0.0 | 0.0 |
| 2015 | New York | Livingston | 40.0 | 0.0 | 0.0 | 0.0 |
| 2015 | New York | Monroe | 12.0 | 12.0 | 0.0 | 0.0 |
| 2015 | New York | Montgomery | 15.3 | 0.0 | 1.7 | 0.0 |
| 2015 | New York | Nassau | 0.0 | 0.0 | 0.0 | 0.0 |
| 2015 | New York | Onondaga | 16.2 | 1.1 | 0.0 | 0.0 |
| 2015 | New York | Orange | 44.0 | 4.0 | 2.0 | 0.0 |
| 2015 | New York | Orleans | 0.0 | 0.0 | 0.0 | 0.0 |
| 2015 | New York | Oswego | 16.7 | 0.0 | 0.0 | 0.0 |
| 2015 | New York | Otsego | 42.0 | 0.0 | 4.0 | 0.0 |
| 2015 | New York | Rensselaer | 31.4 | 9.8 | 0.0 | 0.0 |
| 2015 | New York | Rockland | 14.0 | 0.0 | 0.0 | 20.0 |
| 2015 | New York | Saratoga | 34.2 | 6.1 | 1.8 | 0.9 |
| 2015 | New York | Schenectady | 22.2 | 0.0 | 0.0 | 0.0 |
| 2015 | New York | Schoharie | 41.0 | 0.0 | 0.0 | 0.0 |
| 2015 | New York | Schuyler | 21.8 | 0.0 | 0.0 | 1.8 |
| 2015 | New York | Seneca | 24.1 | 0.0 | 0.0 | 1.9 |
| 2015 | New York | St. Lawrence | 22.2 | 0.0 | 0.0 | 11.1 |
| 2015 | New York | Steuben | 0.0 | 0.0 | 0.0 | 0.0 |
| 2015 | New York | Suffolk | 22.9 | 3.7 | 11.9 | 0.9 |
| 2015 | New York | Sullivan | 34.0 | 0.0 | 6.0 | 2.0 |
| 2015 | New York | Tioga | 0.0 | 0.0 | 0.0 | 0.0 |
| 2015 | New York | Tompkins | 14.3 | 0.0 | 0.0 | 0.0 |
| 2015 | New York | Ulster | 12.0 | 4.0 | 4.0 | 0.0 |
| 2015 | New York | Warren | 30.0 | 0.0 | 0.0 | 0.0 |
| 2015 | New York | Washington | 29.8 | 3.5 | 1.8 | 0.0 |
| 2015 | New York | Wayne | 16.7 | 0.0 | 0.0 | 0.0 |
| 2015 | New York | Westchester | 6.0 | 28.0 | 6.0 | 0.0 |
| 2015 | New York | Wyoming | 30.8 | 0.0 | 0.0 | 0.0 |
| 2016 | New York | Albany | 32.0 | 12.0 | 10.7 | 1.3 |
| 2016 | New York | Allegany | 0.0 | 0.0 | 0.0 | 0.0 |
| 2016 | New York | Broome | 50.0 | 0.0 | 0.0 | 0.0 |
| 2016 | New York | Cattaraugus | 12.9 | 2.5 | 0.0 | 0.0 |
| 2016 | New York | Cayuga | 0.0 | 0.0 | 0.0 | 0.0 |
| 2016 | New York | Chautauqua | 0.0 | 0.0 | 0.0 | 0.0 |
| 2016 | New York | Chemung | 30.0 | 0.0 | 0.0 | 2.0 |
| 2016 | New York | Chenango | 0.0 | 9.1 | 0.0 | 0.0 |
| 2016 | New York | Columbia | 28.6 | 14.3 | 6.3 | 1.6 |
| 2016 | New York | Delaware | 20.7 | 0.0 | 0.0 | 0.0 |
| 2016 | New York | Dutchess | 23.5 | 5.9 | 17.6 | 0.0 |
| 2016 | New York | Erie | 30.5 | 3.4 | 0.0 | 0.0 |
| 2016 | New York | Essex | 42.9 | 0.0 | 0.0 | 0.0 |
| 2016 | New York | Franklin | 35.7 | 0.0 | 0.0 | 0.0 |
| 2016 | New York | Fulton | 16.7 | 0.0 | 0.0 | 0.0 |
| 2016 | New York | Greene | 21.8 | 16.4 | 12.7 | 5.5 |
| 2016 | New York | Jefferson | 0.0 | 0.0 | 0.0 | 0.0 |
| 2016 | New York | Monroe | 15.7 | 17.3 | 0.0 | 0.0 |
| 2016 | New York | Montgomery | 19.7 | 3.0 | 0.0 | 4.5 |
| 2016 | New York | Niagara | 0.0 | 0.0 | 0.0 | 0.0 |
| 2016 | New York | Onondaga | 24.5 | 0.0 | 0.0 | 0.0 |
| 2016 | New York | Orange | 57.4 | 3.7 | 7.4 | 1.9 |
| 2016 | New York | Oswego | 20.0 | 0.0 | 0.0 | 0.0 |
| 2016 | New York | Otsego | 25.4 | 0.0 | 1.7 | 0.0 |
| 2016 | New York | Rensselaer | 23.9 | 4.5 | 0.0 | 1.5 |
| 2016 | New York | Rockland | 45.0 | 3.3 | 25.0 | 0.0 |
| 2016 | New York | Saratoga | 35.0 | 7.0 | 1.0 | 3.0 |
| 2016 | New York | Schenectady | 22.4 | 1.3 | 0.0 | 0.0 |
| 2016 | New York | Schoharie | 37.5 | 1.8 | 0.0 | 0.0 |
| 2016 | New York | Schuyler | 24.0 | 0.0 | 0.0 | 0.0 |
| 2016 | New York | Seneca | 30.0 | 0.0 | 0.0 | 10.0 |
| 2016 | New York | St. Lawrence | 20.0 | 0.0 | 0.0 | 0.0 |
| 2016 | New York | Suffolk | 36.8 | 6.9 | 25.0 | 3.9 |
| 2016 | New York | Sullivan | 60.0 | 22.0 | 8.0 | 0.0 |
| 2016 | New York | Tioga | 50.0 | 0.0 | 0.0 | 0.0 |
| 2016 | New York | Tompkins | 0.0 | 0.0 | 0.0 | 0.0 |
| 2016 | New York | Ulster | 20.8 | 8.3 | 8.3 | 0.0 |
| 2016 | New York | Warren | 12.0 | 0.0 | 1.3 | 0.0 |
| 2016 | New York | Washington | 26.3 | 0.0 | 7.0 | 1.8 |
| 2016 | New York | Westchester | 13.5 | 7.7 | 11.5 | 1.9 |
| 2016 | New York | Wyoming | 14.7 | 2.9 | 0.0 | 0.0 |
| 2017 | New York | Albany | 21.7 | 6.6 | 4.7 | 1.9 |
| 2017 | New York | Allegany | 23.1 | 0.0 | 0.0 | 0.0 |
| 2017 | New York | Broome | 32.1 | 0.0 | 0.0 | 0.0 |
| 2017 | New York | Cattaraugus | 21.0 | 1.9 | 0.6 | 0.6 |
| 2017 | New York | Cayuga | 22.0 | 0.0 | 0.0 | 2.0 |
| 2017 | New York | Chautauqua | 0.0 | 0.0 | 0.0 | 0.0 |
| 2017 | New York | Chemung | 40.0 | 20.0 | 0.0 | 0.0 |
| 2017 | New York | Clinton | 24.5 | 1.9 | 1.9 | 1.9 |
| 2017 | New York | Columbia | 21.1 | 9.6 | 15.8 | 0.0 |
| 2017 | New York | Cortland | 26.9 | 1.9 | 0.0 | 0.0 |
| 2017 | New York | Delaware | 27.9 | 4.9 | 0.0 | 0.0 |
| 2017 | New York | Dutchess | 28.0 | 4.0 | 12.0 | 2.0 |
| 2017 | New York | Erie | 28.6 | 0.0 | 0.0 | 0.0 |
| 2017 | New York | Essex | 22.0 | 0.0 | 6.0 | 0.0 |
| 2017 | New York | Franklin | 14.0 | 0.0 | 0.0 | 0.0 |
| 2017 | New York | Fulton | 10.0 | 0.0 | 10.0 | 0.0 |
| 2017 | New York | Genesee | 0.0 | 0.0 | 0.0 | 0.0 |
| 2017 | New York | Greene | 24.1 | 4.5 | 2.7 | 0.0 |
| 2017 | New York | Herkimer | 24.0 | 0.0 | 0.0 | 0.0 |
| 2017 | New York | Jefferson | 64.5 | 0.0 | 6.5 | 0.0 |
| 2017 | New York | Livingston | 8.3 | 4.2 | 0.0 | 0.0 |
| 2017 | New York | Monroe | 23.9 | 3.3 | 0.0 | 0.0 |
| 2017 | New York | Montgomery | 27.9 | 1.5 | 1.5 | 0.0 |
| 2017 | New York | Niagara | 0.0 | 0.0 | 0.0 | 0.0 |
| 2017 | New York | Oneida | 12.5 | 0.0 | 0.0 | 0.0 |
| 2017 | New York | Onondaga | 30.0 | 0.0 | 0.0 | 2.0 |
| 2017 | New York | Ontario | 34.9 | 2.1 | 2.1 | 0.0 |
| 2017 | New York | Orange | 42.0 | 2.0 | 8.0 | 2.0 |
| 2017 | New York | Orleans | 16.7 | 0.0 | 0.0 | 0.0 |
| 2017 | New York | Oswego | 29.1 | 0.0 | 0.0 | 0.0 |
| 2017 | New York | Otsego | 25.2 | 0.0 | 1.8 | 0.0 |
| 2017 | New York | Rensselaer | 35.3 | 11.2 | 3.4 | 1.7 |
| 2017 | New York | Rockland | 8.2 | 2.0 | 6.1 | 2.0 |
| 2017 | New York | Saratoga | 23.9 | 6.0 | 3.0 | 1.5 |
| 2017 | New York | Schenectady | 16.8 | 0.9 | 0.9 | 0.0 |
| 2017 | New York | Schoharie | 23.4 | 0.0 | 1.6 | 1.6 |
| 2017 | New York | Schuyler | 20.8 | 0.0 | 0.0 | 0.0 |
| 2017 | New York | Seneca | 23.1 | 0.0 | 0.0 | 0.0 |
| 2017 | New York | St. Lawrence | 29.4 | 0.0 | 0.0 | 0.0 |
| 2017 | New York | Steuben | 40.0 | 0.0 | 0.0 | 0.0 |
| 2017 | New York | Suffolk | 35.6 | 13.8 | 24.7 | 3.3 |
| 2017 | New York | Sullivan | 39.2 | 0.0 | 11.8 | 0.0 |
| 2017 | New York | Tompkins | 30.0 | 0.0 | 0.0 | 2.0 |
| 2017 | New York | Ulster | 24.0 | 12.0 | 4.0 | 0.0 |
| 2017 | New York | Warren | 18.8 | 0.0 | 0.0 | 0.0 |
| 2017 | New York | Washington | 31.6 | 6.6 | 2.6 | 0.0 |
| 2017 | New York | Westchester | 36.0 | 12.0 | 38.0 | 2.0 |
| 2017 | New York | Wyoming | 21.5 | 0.0 | 0.0 | 0.0 |
| 2017 | New York | Yates | 16.7 | 0.0 | 0.0 | 0.0 |
| 2018 | New York | Albany | 26.7 | 5.0 | 6.9 | 4.0 |
| 2018 | New York | Allegany | 25.0 | 0.0 | 0.0 | 0.0 |
| 2018 | New York | Cattaraugus | 22.9 | 4.3 | 0.0 | 0.0 |
| 2018 | New York | Chautauqua | 5.9 | 0.0 | 0.0 | 0.0 |
| 2018 | New York | Chemung | 33.3 | 0.0 | 0.0 | 0.0 |
| 2018 | New York | Clinton | 34.4 | 0.0 | 0.0 | 0.0 |
| 2018 | New York | Columbia | 20.4 | 1.9 | 16.7 | 0.0 |
| 2018 | New York | Delaware | 30.2 | 0.0 | 0.0 | 0.0 |
| 2018 | New York | Dutchess | 28.0 | 4.0 | 8.0 | 2.0 |
| 2018 | New York | Erie | 20.0 | 0.0 | 0.0 | 0.0 |
| 2018 | New York | Essex | 66.7 | 0.0 | 0.0 | 0.0 |
| 2018 | New York | Franklin | 24.3 | 0.0 | 2.7 | 0.0 |
| 2018 | New York | Fulton | 0.0 | 0.0 | 25.0 | 0.0 |
| 2018 | New York | Greene | 22.5 | 8.8 | 11.8 | 3.9 |
| 2018 | New York | Hamilton | 0.0 | 0.0 | 0.0 | 0.0 |
| 2018 | New York | Livingston | 0.0 | 50.0 | 0.0 | 0.0 |
| 2018 | New York | Monroe | 19.8 | 3.0 | 1.0 | 3.0 |
| 2018 | New York | Montgomery | 25.3 | 3.8 | 7.6 | 0.0 |
| 2018 | New York | Niagara | 0.0 | 0.0 | 0.0 | 0.0 |
| 2018 | New York | Onondaga | 30.0 | 0.0 | 0.0 | 0.0 |
| 2018 | New York | Orange | 36.0 | 16.0 | 18.0 | 2.0 |
| 2018 | New York | Orleans | 50.0 | 0.0 | 0.0 | 0.0 |
| 2018 | New York | Oswego | 40.0 | 0.0 | 0.0 | 0.0 |
| 2018 | New York | Otsego | 27.9 | 0.0 | 1.6 | 0.0 |
| 2018 | New York | Rensselaer | 34.6 | 0.0 | 11.5 | 0.0 |
| 2018 | New York | Rockland | 8.0 | 10.0 | 14.0 | 6.0 |
| 2018 | New York | Saratoga | 22.6 | 2.6 | 3.5 | 1.7 |
| 2018 | New York | Schenectady | 18.7 | 13.2 | 3.3 | 0.0 |
| 2018 | New York | Schoharie | 37.1 | 8.6 | 10.0 | 0.0 |
| 2018 | New York | Schuyler | 18.4 | 2.0 | 0.0 | 0.0 |
| 2018 | New York | Steuben | 0.0 | 0.0 | 0.0 | 0.0 |
| 2018 | New York | Suffolk | 27.1 | 3.5 | 24.2 | 2.9 |
| 2018 | New York | Sullivan | 30.0 | 0.0 | 2.0 | 0.0 |
| 2018 | New York | Tioga | 19.4 | 3.0 | 0.0 | 3.0 |
| 2018 | New York | Tompkins | 30.0 | 2.0 | 0.0 | 0.0 |
| 2018 | New York | Ulster | 24.0 | 2.0 | 6.0 | 4.0 |
| 2018 | New York | Warren | 11.8 | 19.6 | 3.9 | 0.0 |
| 2018 | New York | Washington | 23.8 | 3.2 | 1.6 | 1.6 |
| 2018 | New York | Westchester | 20.0 | 10.0 | 26.0 | 2.0 |
| 2018 | New York | Wyoming | 11.1 | 0.0 | 0.0 | 0.0 |
| 2019 | Connecticut | Fairfield | 15.7 | 5.7 | 5.7 | 0.0 |
| 2019 | Connecticut | Hartford | 18.8 | 3.1 | 3.1 | 3.1 |
| 2019 | Connecticut | Litchfield | 24.7 | 4.1 | 8.2 | 4.1 |
| 2019 | Connecticut | Middlesex | 24.2 | 0.0 | 12.1 | 3.0 |
| 2019 | Connecticut | New Haven | 11.5 | 3.3 | 8.2 | 0.0 |
| 2019 | Connecticut | New London | 13.4 | 4.5 | 10.4 | 0.0 |
| 2019 | Connecticut | Tolland | 13.6 | 8.0 | 5.5 | 1.0 |
| 2019 | Connecticut | Windham | 10.9 | 3.8 | 3.8 | 2.2 |
| 2019 | Maine | Cumberland | 10.5 | NA | NA | NA |
| 2019 | Maine | Hancock | 17.0 | NA | NA | NA |
| 2019 | Maine | Kennebec | 28.3 | NA | NA | NA |
| 2019 | Maine | Knox | 30.6 | NA | NA | NA |
| 2019 | Maine | Lincoln | 32.3 | NA | NA | NA |
| 2019 | Maine | Oxford | 0.0 | NA | NA | NA |
| 2019 | Maine | Penobscot | 6.3 | NA | NA | NA |
| 2019 | Maine | Sagadahoc | 24.0 | NA | NA | NA |
| 2019 | Maine | Somerset | 18.2 | NA | NA | NA |
| 2019 | Maine | Waldo | 29.1 | NA | NA | NA |
| 2019 | Maine | Washington | 16.0 | NA | NA | NA |
| 2019 | Maine | York | 28.0 | NA | NA | NA |
| 2019 | New York | Albany | 20.2 | 5.5 | 6.4 | 1.4 |
| 2019 | New York | Allegany | 30.4 | 3.6 | 0.0 | 0.0 |
| 2019 | New York | Cattaraugus | 25.7 | 7.1 | 0.0 | 0.0 |
| 2019 | New York | Chautauqua | 21.7 | 0.0 | 0.0 | 0.0 |
| 2019 | New York | Chemung | 14.3 | 7.1 | 0.0 | 0.0 |
| 2019 | New York | Chenango | 0.0 | 0.0 | 0.0 | 0.0 |
| 2019 | New York | Clinton | 30.9 | 0.0 | 5.5 | 1.8 |
| 2019 | New York | Columbia | 23.9 | 5.7 | 15.9 | 0.0 |
| 2019 | New York | Cortland | 26.9 | 1.9 | 0.0 | 0.0 |
| 2019 | New York | Delaware | 33.6 | 0.9 | 0.9 | 0.0 |
| 2019 | New York | Dutchess | 16.0 | 12.0 | 4.0 | 0.0 |
| 2019 | New York | Erie | 28.8 | 7.7 | 0.0 | 0.0 |
| 2019 | New York | Essex | 32.1 | 1.2 | 6.2 | 0.0 |
| 2019 | New York | Franklin | 26.0 | 2.0 | 0.0 | 0.0 |
| 2019 | New York | Fulton | 24.0 | 0.0 | 0.0 | 0.8 |
| 2019 | New York | Genesee | 12.5 | 0.0 | 0.0 | 0.0 |
| 2019 | New York | Greene | 29.2 | 7.7 | 9.9 | 1.3 |
| 2019 | New York | Jefferson | 26.0 | 0.0 | 0.0 | 0.0 |
| 2019 | New York | Lewis | 11.5 | 1.9 | 0.0 | 0.0 |
| 2019 | New York | Livingston | 17.6 | 3.9 | 0.0 | 0.0 |
| 2019 | New York | Madison | 0.0 | 0.0 | 0.0 | 0.0 |
| 2019 | New York | Monroe | 27.0 | 9.0 | 0.0 | 1.0 |
| 2019 | New York | Montgomery | 30.0 | 2.2 | 3.0 | 1.3 |
| 2019 | New York | Niagara | 12.3 | 2.7 | 0.0 | 1.4 |
| 2019 | New York | Onondaga | 25.4 | 1.6 | 0.0 | 0.0 |
| 2019 | New York | Ontario | 48.0 | 0.0 | 0.0 | 0.0 |
| 2019 | New York | Orange | 40.0 | 2.0 | 14.0 | 0.0 |
| 2019 | New York | Orleans | 33.3 | 0.0 | 0.0 | 0.0 |
| 2019 | New York | Oswego | 42.0 | 0.0 | 0.0 | 2.0 |
| 2019 | New York | Otsego | 26.5 | 0.8 | 0.8 | 0.8 |
| 2019 | New York | Rensselaer | 19.3 | 6.4 | 7.0 | 1.2 |
| 2019 | New York | Rockland | 14.0 | 4.0 | 20.0 | 0.0 |
| 2019 | New York | Saratoga | 24.9 | 5.9 | 9.3 | 0.4 |
| 2019 | New York | Schenectady | 20.4 | 4.9 | 2.1 | 0.7 |
| 2019 | New York | Schoharie | 23.4 | 9.0 | 2.0 | 0.0 |
| 2019 | New York | Schuyler | 25.5 | 2.1 | 4.3 | 0.0 |
| 2019 | New York | St. Lawrence | 12.0 | 0.0 | 0.0 | 2.0 |
| 2019 | New York | Steuben | 16.7 | 0.0 | 0.0 | 0.0 |
| 2019 | New York | Suffolk | 21.8 | 8.0 | 14.2 | 2.9 |
| 2019 | New York | Sullivan | 36.0 | 4.0 | 8.0 | 0.0 |
| 2019 | New York | Tompkins | 46.0 | 2.0 | 0.0 | 2.0 |
| 2019 | New York | Ulster | 12.0 | 0.0 | 0.0 | 4.0 |
| 2019 | New York | Warren | 30.1 | 1.9 | 0.0 | 1.0 |
| 2019 | New York | Washington | 21.6 | 5.4 | 6.8 | 0.0 |
| 2019 | New York | Westchester | 16.0 | 8.0 | 18.0 | 0.0 |
| 2019 | New York | Wyoming | 35.3 | 0.0 | 0.0 | 0.0 |
| 2020 | Connecticut | Fairfield | 4.2 | 12.5 | 0.0 | 0.0 |
| 2020 | Connecticut | Hartford | 22.6 | 1.9 | 15.1 | 1.9 |
| 2020 | Connecticut | Litchfield | 17.9 | 7.7 | 0.0 | 0.0 |
| 2020 | Connecticut | Middlesex | 8.5 | 0.0 | 19.1 | 2.1 |
| 2020 | Connecticut | New Haven | 12.1 | 2.2 | 3.3 | 1.1 |
| 2020 | Connecticut | New London | 23.5 | 8.8 | 10.8 | 1.0 |
| 2020 | Connecticut | Tolland | 26.0 | 2.1 | 5.2 | 1.0 |
| 2020 | Connecticut | Windham | 29.0 | 3.2 | 1.1 | 1.1 |
| 2020 | Maine | Androscoggin | 7.1 | 8.3 | 0.0 | NA |
| 2020 | Maine | Cumberland | 39.5 | 5.4 | 10.8 | NA |
| 2020 | Maine | Hancock | 30.0 | 0.0 | 0.0 | NA |
| 2020 | Maine | Kennebec | 23.3 | 9.1 | 6.1 | NA |
| 2020 | Maine | Knox | 23.1 | 10.3 | 8.5 | NA |
| 2020 | Maine | Lincoln | 21.4 | 0.0 | 0.0 | NA |
| 2020 | Maine | Sagadahoc | 30.1 | 3.8 | 1.9 | NA |
| 2020 | Maine | Somerset | 50.0 | NA | NA | NA |
| 2020 | Maine | Waldo | 26.1 | 4.3 | 2.2 | NA |
| 2020 | Maine | Washington | 50.0 | NA | NA | NA |
| 2020 | Maine | York | 26.5 | 9.5 | 4.8 | NA |
| 2020 | New York | Albany | 25.7 | 7.6 | 6.3 | 0.7 |
| 2020 | New York | Allegany | 26.9 | 5.8 | 0.0 | 0.0 |
| 2020 | New York | Cattaraugus | 32.3 | 5.8 | 0.0 | 0.0 |
| 2020 | New York | Cayuga | 0.0 | 0.0 | 0.0 | 0.0 |
| 2020 | New York | Chautauqua | 21.5 | 2.8 | 0.0 | 0.0 |
| 2020 | New York | Clinton | 29.7 | 2.7 | 0.0 | 0.0 |
| 2020 | New York | Columbia | 16.3 | 8.1 | 12.8 | 0.6 |
| 2020 | New York | Delaware | 31.5 | 1.9 | 0.0 | 0.0 |
| 2020 | New York | Dutchess | 25.0 | 2.8 | 15.3 | 0.0 |
| 2020 | New York | Erie | 44.4 | 0.0 | 0.0 | 0.0 |
| 2020 | New York | Essex | 25.8 | 12.9 | 9.7 | 0.0 |
| 2020 | New York | Franklin | 46.2 | 7.7 | 0.0 | 0.0 |
| 2020 | New York | Fulton | 32.1 | 10.7 | 0.0 | 0.0 |
| 2020 | New York | Genesee | 0.0 | 0.0 | 0.0 | 0.0 |
| 2020 | New York | Greene | 25.2 | 6.7 | 3.4 | 3.4 |
| 2020 | New York | Hamilton | 0.0 | 0.0 | 0.0 | 0.0 |
| 2020 | New York | Montgomery | 20.0 | 0.0 | 3.2 | 0.0 |
| 2020 | New York | Onondaga | 26.0 | 18.0 | 0.0 | 0.0 |
| 2020 | New York | Oswego | 50.0 | 0.0 | 0.0 | 0.0 |
| 2020 | New York | Otsego | 29.3 | 5.2 | 0.0 | 0.0 |
| 2020 | New York | Rensselaer | 50.0 | 8.6 | 8.6 | 6.9 |
| 2020 | New York | Rockland | 23.5 | 7.8 | 9.8 | 2.0 |
| 2020 | New York | Saratoga | 17.2 | 11.5 | 6.9 | 2.3 |
| 2020 | New York | Schenectady | 22.5 | 1.1 | 1.1 | 3.4 |
| 2020 | New York | Schoharie | 40.6 | 3.1 | 6.3 | 0.0 |
| 2020 | New York | St. Lawrence | 33.3 | 13.3 | 0.0 | 0.0 |
| 2020 | New York | Steuben | 28.6 | 0.0 | 0.0 | 0.0 |
| 2020 | New York | Suffolk | 22.2 | 8.4 | 23.1 | 5.5 |
| 2020 | New York | Tompkins | 42.0 | 14.0 | 2.0 | 0.0 |
| 2020 | New York | Ulster | 27.3 | 9.1 | 0.0 | 9.1 |
| 2020 | New York | Warren | 25.9 | 3.4 | 1.7 | 0.0 |
| 2020 | New York | Washington | 16.1 | 8.1 | 8.1 | 1.6 |
| 2020 | New York | Westchester | 26.0 | 2.0 | 18.0 | 2.0 |
| 2021 | Connecticut | Fairfield | 15.6 | 4.4 | 2.2 | 0.0 |
| 2021 | Connecticut | Hartford | 16.3 | 2.0 | 10.2 | 0.0 |
| 2021 | Connecticut | Litchfield | 19.3 | 1.8 | 5.3 | 0.0 |
| 2021 | Connecticut | Middlesex | 20.3 | 2.9 | 5.8 | 4.3 |
| 2021 | Connecticut | New Haven | 24.4 | 13.3 | 6.7 | 1.1 |
| 2021 | Connecticut | New London | 26.5 | 8.2 | 16.3 | 0.0 |
| 2021 | Connecticut | Tolland | 21.8 | 5.9 | 10.9 | 4.0 |
| 2021 | Connecticut | Windham | 27.5 | 2.5 | 10.0 | 5.0 |
| 2021 | Maine | Androscoggin | 17.2 | 3.4 | 6.9 | NA |
| 2021 | Maine | Cumberland | 22.9 | 8.0 | 4.4 | NA |
| 2021 | Maine | Hancock | 20.1 | 1.3 | 0.0 | NA |
| 2021 | Maine | Kennebec | 24.2 | 5.3 | 4.3 | NA |
| 2021 | Maine | Knox | 31.0 | 7.7 | 9.1 | NA |
| 2021 | Maine | Lincoln | 32.3 | 13.1 | 19.2 | NA |
| 2021 | Maine | Penobscot | 15.2 | 0.0 | 0.0 | NA |
| 2021 | Maine | Sagadahoc | 22.5 | 3.5 | 5.1 | NA |
| 2021 | Maine | Somerset | 27.3 | 4.5 | 0.0 | NA |
| 2021 | Maine | Waldo | 18.9 | 5.3 | 3.7 | NA |
| 2021 | Maine | York | 28.2 | 7.7 | 6.4 | NA |
| 2021 | New York | Albany | 33.0 | 9.1 | 11.4 | 2.3 |
| 2021 | New York | Allegany | 36.0 | 8.0 | 0.0 | 0.0 |
| 2021 | New York | Cattaraugus | 31.2 | 8.6 | 0.0 | 0.9 |
| 2021 | New York | Chautauqua | 50.0 | 0.0 | 0.0 | 0.0 |
| 2021 | New York | Chemung | 25.0 | 5.0 | 5.0 | 0.0 |
| 2021 | New York | Clinton | 37.7 | 4.9 | 8.2 | 1.6 |
| 2021 | New York | Columbia | 19.2 | 13.9 | 6.0 | 0.7 |
| 2021 | New York | Delaware | 39.0 | 6.8 | 0.0 | 0.0 |
| 2021 | New York | Dutchess | 25.0 | 6.0 | 15.0 | 1.0 |
| 2021 | New York | Erie | 38.6 | 3.0 | 0.0 | 1.0 |
| 2021 | New York | Essex | 14.3 | 7.1 | 3.6 | 0.0 |
| 2021 | New York | Franklin | 32.0 | 4.0 | 6.0 | 2.0 |
| 2021 | New York | Fulton | 22.6 | 3.2 | 0.0 | 0.0 |
| 2021 | New York | Genesee | 22.6 | 0.0 | 0.0 | 0.0 |
| 2021 | New York | Greene | 23.9 | 8.8 | 7.5 | 0.9 |
| 2021 | New York | Hamilton | 66.7 | 0.0 | 0.0 | 0.0 |
| 2021 | New York | Livingston | 6.7 | 0.0 | 0.0 | 0.0 |
| 2021 | New York | Monroe | 16.0 | 0.0 | 0.0 | 0.0 |
| 2021 | New York | Montgomery | 29.3 | 2.7 | 2.7 | 0.0 |
| 2021 | New York | Niagara | 8.8 | 0.0 | 2.9 | 0.0 |
| 2021 | New York | Onondaga | 22.0 | 26.0 | 0.0 | 0.0 |
| 2021 | New York | Ontario | 26.0 | 4.0 | 0.0 | 0.0 |
| 2021 | New York | Orange | 28.0 | 12.0 | 32.0 | 0.0 |
| 2021 | New York | Orleans | 20.0 | 0.0 | 0.0 | 0.0 |
| 2021 | New York | Oswego | 28.0 | 0.0 | 0.0 | 0.0 |
| 2021 | New York | Otsego | 36.3 | 6.6 | 4.4 | 0.0 |
| 2021 | New York | Rensselaer | 35.3 | 9.3 | 8.0 | 3.3 |
| 2021 | New York | Rockland | 28.0 | 4.0 | 10.0 | 0.0 |
| 2021 | New York | Saratoga | 28.8 | 5.6 | 12.0 | 1.6 |
| 2021 | New York | Schenectady | 22.6 | 4.8 | 6.0 | 1.2 |
| 2021 | New York | Schoharie | 24.8 | 5.7 | 9.5 | 0.0 |
| 2021 | New York | Schuyler | 42.9 | 0.0 | 0.0 | 0.0 |
| 2021 | New York | St. Lawrence | 38.0 | 6.0 | 0.0 | 0.0 |
| 2021 | New York | Suffolk | 26.4 | 7.3 | 19.6 | 2.7 |
| 2021 | New York | Sullivan | 10.0 | 6.0 | 4.0 | 0.0 |
| 2021 | New York | Tompkins | 22.0 | 6.0 | 2.0 | 0.0 |
| 2021 | New York | Ulster | 16.0 | 4.0 | 4.0 | 4.0 |
| 2021 | New York | Warren | 24.6 | 3.1 | 7.7 | 1.5 |
| 2021 | New York | Washington | 33.0 | 9.6 | 7.4 | 1.1 |
| 2021 | New York | Wayne | 30.0 | 0.0 | 0.0 | 2.0 |
| 2021 | New York | Westchester | 12.0 | 14.0 | 12.0 | 6.0 |
| 2021 | New York | Wyoming | 20.8 | 4.2 | 0.0 | 2.1 |
| 2021 | New York | Yates | 27.3 | 9.1 | 0.0 | 0.0 |

Table S4. Adult *Ixodes scapularis* percent pathogen prevalence from ticks collected using active tick surveillance methods (tick dragging or flagging) in Connecticut, Maine, New Hampshire, New York, and Vermont.

| Year | State | County | *B. burgdorferi* | *A. phagocytophilum* | *B. microti* | *B. miyamotoi* |
| --- | --- | --- | --- | --- | --- | --- |
| 1990 | Maine | Lincoln | 41.7 | NA | NA | NA |
| 1991 | Maine | Knox | 32.5 | NA | NA | NA |
| 1991 | Maine | Lincoln | 39.8 | NA | NA | NA |
| 1991 | Maine | York | 67.9 | NA | NA | NA |
| 1992 | Maine | Cumberland | 8.3 | NA | NA | NA |
| 1992 | Maine | Knox | 35.4 | NA | NA | NA |
| 1992 | Maine | Lincoln | 35.5 | NA | NA | NA |
| 1992 | Maine | York | 35.4 | NA | NA | NA |
| 1993 | Maine | Knox | 14.7 | NA | NA | NA |
| 1993 | Maine | Lincoln | 23.8 | NA | NA | NA |
| 1993 | Maine | York | 37.5 | NA | NA | NA |
| 1994 | Maine | Hancock | 0.0 | NA | NA | NA |
| 1994 | Maine | Knox | 26.5 | NA | NA | NA |
| 1994 | Maine | Lincoln | 19.9 | NA | NA | NA |
| 1994 | Maine | York | 56.0 | NA | NA | NA |
| 1995 | Maine | Cumberland | 31.4 | NA | NA | NA |
| 1995 | Maine | Kennebec | 0.0 | NA | NA | NA |
| 1995 | Maine | Lincoln | 32.3 | NA | NA | NA |
| 1995 | Maine | York | 52.2 | NA | NA | NA |
| 1996 | Maine | Cumberland | 38.4 | NA | NA | NA |
| 1996 | Maine | Lincoln | 28.6 | NA | NA | NA |
| 1997 | Maine | Hancock | 0.0 | NA | NA | NA |
| 1997 | Maine | Knox | 16.6 | NA | NA | NA |
| 1997 | Maine | Lincoln | 39.0 | NA | NA | NA |
| 1997 | Maine | Sagadahoc | 44.3 | NA | NA | NA |
| 1997 | Maine | Waldo | 0.0 | NA | NA | NA |
| 1997 | Maine | York | 59.3 | NA | NA | NA |
| 1998 | Maine | Knox | 7.6 | NA | NA | NA |
| 1998 | Maine | Lincoln | 61.2 | NA | NA | NA |
| 1998 | Maine | York | 49.7 | NA | NA | NA |
| 1999 | Maine | Cumberland | 51.4 | NA | NA | NA |
| 1999 | Maine | Knox | 19.9 | NA | NA | NA |
| 1999 | Maine | Lincoln | 77.2 | NA | NA | NA |
| 1999 | Maine | York | 58.3 | NA | NA | NA |
| 2000 | Maine | Cumberland | 61.4 | NA | NA | NA |
| 2000 | Maine | Hancock | 4.2 | NA | NA | NA |
| 2000 | Maine | Knox | 19.6 | NA | NA | NA |
| 2000 | Maine | Lincoln | 57.1 | NA | NA | NA |
| 2000 | Maine | York | 62.4 | NA | NA | NA |
| 2002 | Maine | Cumberland | 60.7 | NA | NA | NA |
| 2002 | Maine | Knox | 22.0 | NA | NA | NA |
| 2002 | Maine | Lincoln | 30.9 | NA | NA | NA |
| 2002 | Maine | York | 57.9 | NA | NA | NA |
| 2003 | Maine | Cumberland | 64.3 | NA | NA | NA |
| 2003 | Maine | Knox | 25.4 | NA | NA | NA |
| 2003 | Maine | Lincoln | 25.9 | NA | NA | NA |
| 2003 | Maine | Sagadahoc | 38.7 | NA | NA | NA |
| 2003 | Maine | York | 58.7 | NA | NA | NA |
| 2004 | Maine | Cumberland | 58.9 | NA | NA | NA |
| 2004 | Maine | Knox | 21.6 | NA | NA | NA |
| 2004 | Maine | Lincoln | 12.1 | NA | NA | NA |
| 2004 | Maine | York | 57.8 | NA | NA | NA |
| 2005 | Maine | Cumberland | 44.7 | NA | NA | NA |
| 2005 | Maine | Sagadahoc | 50.0 | NA | NA | NA |
| 2005 | Maine | York | 50.2 | NA | NA | NA |
| 2006 | Maine | Cumberland | 81.2 | NA | NA | NA |
| 2006 | Maine | Lincoln | 34.0 | NA | NA | NA |
| 2006 | Maine | Waldo | 24.2 | NA | NA | NA |
| 2006 | Maine | York | 68.5 | NA | NA | NA |
| 2007 | Maine | Cumberland | 55.8 | NA | NA | NA |
| 2007 | Maine | Hancock | 43.7 | NA | NA | NA |
| 2007 | Maine | Kennebec | 50.9 | NA | NA | NA |
| 2007 | Maine | Lincoln | 31.9 | NA | NA | NA |
| 2007 | Maine | York | 58.9 | NA | NA | NA |
| 2008 | Maine | Cumberland | 51.0 | NA | NA | NA |
| 2008 | Maine | Lincoln | 31.6 | NA | NA | NA |
| 2008 | Maine | York | 59.2 | NA | NA | NA |
| 2008 | New York | Albany | 57.7 | 1.9 | 0.0 | NA |
| 2008 | New York | Cattaraugus | 1.8 | 0.0 | 0.0 | NA |
| 2008 | New York | Chautauqua | 0.0 | 0.0 | 0.0 | NA |
| 2008 | New York | Chemung | 15.9 | 0.0 | 0.0 | NA |
| 2008 | New York | Columbia | 30.8 | 3.8 | 0.0 | NA |
| 2008 | New York | Dutchess | 20.0 | 4.0 | 2.0 | NA |
| 2008 | New York | Erie | 0.0 | 0.0 | 0.0 | NA |
| 2008 | New York | Herkimer | 27.5 | 0.0 | 0.0 | NA |
| 2008 | New York | Jefferson | 44.0 | 0.0 | 0.0 | NA |
| 2008 | New York | Monroe | 15.7 | 0.0 | 0.0 | NA |
| 2008 | New York | Onondaga | 44.0 | 0.0 | 0.0 | NA |
| 2008 | New York | Orange | 40.0 | 22.0 | 0.0 | NA |
| 2008 | New York | Oswego | 52.0 | 4.0 | 0.0 | NA |
| 2008 | New York | Rockland | 28.0 | 10.0 | 4.0 | NA |
| 2008 | New York | Saratoga | 59.6 | 0.0 | 0.0 | NA |
| 2008 | New York | Schoharie | 4.0 | 0.0 | 0.0 | NA |
| 2008 | New York | Schuyler | 12.1 | 1.1 | 0.0 | NA |
| 2008 | New York | Seneca | 13.5 | 0.0 | 0.0 | NA |
| 2008 | New York | Sullivan | 6.0 | 0.0 | 0.0 | NA |
| 2008 | New York | Tompkins | 18.0 | 8.0 | 0.0 | NA |
| 2008 | New York | Ulster | 32.0 | 8.0 | 0.0 | NA |
| 2008 | New York | Washington | 6.0 | 4.0 | 0.0 | NA |
| 2008 | New York | Westchester | 16.0 | 20.0 | 2.0 | NA |
| 2009 | Maine | Cumberland | 65.7 | NA | NA | NA |
| 2009 | Maine | Waldo | 51.7 | NA | NA | NA |
| 2009 | Maine | York | 65.8 | NA | NA | NA |
| 2009 | New Hampshire | Hillsborough | 40.0 | NA | NA | NA |
| 2009 | New Hampshire | Merrimack | 100.0 | NA | NA | NA |
| 2009 | New Hampshire | Rockingham | 33.3 | NA | NA | NA |
| 2009 | New Hampshire | Strafford | 58.3 | NA | NA | NA |
| 2009 | New York | Albany | 38.0 | 4.0 | 4.0 | NA |
| 2009 | New York | Allegany | 33.9 | 4.7 | 0.0 | NA |
| 2009 | New York | Broome | 29.9 | 0.0 | 0.0 | NA |
| 2009 | New York | Cattaraugus | 18.0 | 0.0 | 0.0 | NA |
| 2009 | New York | Chautauqua | 0.0 | 0.0 | 0.0 | NA |
| 2009 | New York | Chemung | 42.9 | 1.4 | 0.0 | NA |
| 2009 | New York | Columbia | 26.0 | 14.0 | 0.0 | NA |
| 2009 | New York | Dutchess | 24.0 | 8.0 | 2.0 | NA |
| 2009 | New York | Erie | 0.0 | 0.0 | 0.0 | NA |
| 2009 | New York | Greene | 48.0 | 10.0 | 0.0 | NA |
| 2009 | New York | Monroe | 1.9 | 0.0 | 0.0 | NA |
| 2009 | New York | Onondaga | 33.9 | 1.7 | 0.0 | NA |
| 2009 | New York | Orange | 34.0 | 12.0 | 0.0 | NA |
| 2009 | New York | Oswego | 29.9 | 0.0 | 0.0 | NA |
| 2009 | New York | Rensselaer | 32.4 | 13.5 | 2.7 | NA |
| 2009 | New York | Rockland | 36.0 | 2.0 | 0.0 | NA |
| 2009 | New York | Saratoga | 30.0 | 2.0 | 0.0 | NA |
| 2009 | New York | Schuyler | 16.1 | 0.0 | 0.0 | NA |
| 2009 | New York | Seneca | 36.7 | 6.7 | 0.0 | NA |
| 2009 | New York | Steuben | 21.4 | 0.0 | 0.0 | NA |
| 2009 | New York | Sullivan | 44.0 | 0.0 | 0.0 | NA |
| 2009 | New York | Ulster | 36.0 | 12.0 | 0.0 | NA |
| 2009 | New York | Warren | 37.3 | 0.0 | 0.0 | NA |
| 2009 | New York | Westchester | 50.0 | 12.0 | 2.0 | NA |
| 2009 | New York | Yates | 0.0 | 0.0 | 0.0 | NA |
| 2010 | Maine | Cumberland | 69.1 | NA | NA | NA |
| 2010 | Maine | Waldo | 47.4 | NA | NA | NA |
| 2010 | Maine | York | 59.9 | NA | NA | NA |
| 2010 | New Hampshire | Hillsborough | 81.3 | NA | NA | NA |
| 2010 | New Hampshire | Merrimack | 81.8 | NA | NA | NA |
| 2010 | New Hampshire | Rockingham | 62.5 | NA | NA | NA |
| 2010 | New Hampshire | Strafford | 50.0 | NA | NA | NA |
| 2010 | New York | Albany | 56.0 | 6.0 | 12.0 | NA |
| 2010 | New York | Cattaraugus | 39.5 | 2.6 | 0.0 | NA |
| 2010 | New York | Chemung | 32.6 | 2.2 | 0.0 | NA |
| 2010 | New York | Columbia | 54.0 | 8.0 | 0.0 | NA |
| 2010 | New York | Dutchess | 36.0 | 10.0 | 2.0 | NA |
| 2010 | New York | Erie | 0.0 | 0.0 | 0.0 | NA |
| 2010 | New York | Lewis | 100.0 | 0.0 | 0.0 | NA |
| 2010 | New York | Monroe | 1.3 | 0.0 | 0.0 | NA |
| 2010 | New York | Onondaga | 38.5 | 0.0 | 0.0 | NA |
| 2010 | New York | Orange | 58.0 | 18.0 | 0.0 | NA |
| 2010 | New York | Oswego | 76.0 | 0.0 | 0.0 | NA |
| 2010 | New York | Rockland | 46.0 | 6.0 | 8.0 | NA |
| 2010 | New York | Saratoga | 69.4 | 0.0 | 0.0 | NA |
| 2010 | New York | Schenectady | 48.8 | 0.0 | 0.0 | NA |
| 2010 | New York | Schuyler | 5.9 | 0.0 | 0.0 | NA |
| 2010 | New York | Seneca | 36.7 | 0.0 | 0.0 | NA |
| 2010 | New York | Ulster | 44.0 | 2.0 | 0.0 | NA |
| 2010 | New York | Washington | 21.3 | 2.1 | 2.1 | NA |
| 2010 | New York | Westchester | 30.0 | 12.0 | 2.0 | NA |
| 2011 | Maine | Androscoggin | 100.0 | NA | NA | NA |
| 2011 | Maine | Cumberland | 53.0 | NA | NA | NA |
| 2011 | Maine | Knox | 25.0 | NA | NA | NA |
| 2011 | Maine | Waldo | 56.2 | NA | NA | NA |
| 2011 | Maine | York | 66.7 | NA | NA | NA |
| 2011 | New Hampshire | Hillsborough | 61.7 | NA | NA | NA |
| 2011 | New Hampshire | Merrimack | 50.0 | NA | NA | NA |
| 2011 | New Hampshire | Rockingham | 50.0 | NA | NA | NA |
| 2011 | New Hampshire | Strafford | 54.1 | NA | NA | NA |
| 2011 | New York | Albany | 48.0 | 20.0 | 4.0 | NA |
| 2011 | New York | Allegany | 33.3 | 0.0 | 0.0 | NA |
| 2011 | New York | Cattaraugus | 29.0 | 1.9 | 0.0 | NA |
| 2011 | New York | Chemung | 46.0 | 0.0 | 0.0 | NA |
| 2011 | New York | Columbia | 34.0 | 4.0 | 0.0 | NA |
| 2011 | New York | Dutchess | 20.0 | 16.0 | 4.0 | NA |
| 2011 | New York | Livingston | 0.0 | 0.0 | 0.0 | NA |
| 2011 | New York | Monroe | 32.4 | 8.8 | 0.0 | NA |
| 2011 | New York | Onondaga | 66.0 | 0.0 | 0.0 | NA |
| 2011 | New York | Orange | 34.0 | 12.0 | 0.0 | NA |
| 2011 | New York | Oswego | 55.8 | 0.0 | 0.0 | NA |
| 2011 | New York | Rockland | 16.0 | 20.0 | 6.0 | NA |
| 2011 | New York | Saratoga | 30.0 | 4.0 | 0.0 | NA |
| 2011 | New York | Schoharie | 11.8 | 0.0 | 0.0 | NA |
| 2011 | New York | Schuyler | 20.0 | 0.0 | 0.0 | NA |
| 2011 | New York | Seneca | 42.3 | 0.0 | 0.0 | NA |
| 2011 | New York | Sullivan | 32.0 | 10.0 | 0.0 | NA |
| 2011 | New York | Ulster | 32.0 | 4.0 | 0.0 | NA |
| 2011 | New York | Westchester | 10.0 | 16.0 | 0.0 | NA |
| 2012 | Maine | Cumberland | 26.4 | NA | NA | NA |
| 2012 | Maine | Hancock | 46.7 | NA | NA | NA |
| 2012 | Maine | Waldo | 51.3 | NA | NA | NA |
| 2012 | New York | Albany | 57.3 | 17.3 | 6.0 | NA |
| 2012 | New York | Allegany | 17.0 | 0.0 | 0.0 | NA |
| 2012 | New York | Cattaraugus | 24.7 | 0.0 | 0.0 | NA |
| 2012 | New York | Chautauqua | 20.8 | 1.9 | 0.0 | NA |
| 2012 | New York | Chemung | 54.0 | 2.0 | 0.0 | NA |
| 2012 | New York | Clinton | 44.8 | 0.0 | 0.0 | NA |
| 2012 | New York | Columbia | 54.8 | 10.6 | 0.8 | NA |
| 2012 | New York | Dutchess | 82.0 | 10.0 | 8.0 | NA |
| 2012 | New York | Erie | 32.1 | 1.8 | 0.0 | NA |
| 2012 | New York | Essex | 25.0 | 0.0 | 0.0 | NA |
| 2012 | New York | Fulton | 0.0 | 0.0 | 0.0 | NA |
| 2012 | New York | Livingston | 2.0 | 0.0 | 0.0 | NA |
| 2012 | New York | Monroe | 39.1 | 0.0 | 0.0 | NA |
| 2012 | New York | Montgomery | 43.4 | 0.0 | 0.0 | NA |
| 2012 | New York | Onondaga | 55.6 | 0.0 | 0.0 | NA |
| 2012 | New York | Orange | 54.0 | 14.0 | 0.0 | NA |
| 2012 | New York | Oswego | 33.3 | 0.0 | 0.0 | NA |
| 2012 | New York | Rockland | 68.0 | 12.0 | 2.0 | NA |
| 2012 | New York | Saratoga | 64.0 | 18.0 | 0.0 | NA |
| 2012 | New York | Schoharie | 49.2 | 0.0 | 0.0 | NA |
| 2012 | New York | Schuyler | 46.5 | 0.0 | 0.0 | NA |
| 2012 | New York | Seneca | 54.1 | 0.0 | 0.0 | NA |
| 2012 | New York | Steuben | 37.5 | 0.0 | 0.0 | NA |
| 2012 | New York | Sullivan | 66.0 | 18.0 | 0.0 | NA |
| 2012 | New York | Ulster | 42.0 | 6.0 | 4.0 | NA |
| 2012 | New York | Warren | 58.0 | 2.0 | 0.0 | NA |
| 2012 | New York | Westchester | 40.0 | 10.0 | 6.0 | NA |
| 2013 | Maine | Cumberland | 50.8 | NA | NA | NA |
| 2013 | Maine | Hancock | 25.6 | NA | NA | NA |
| 2013 | Maine | Waldo | 37.0 | NA | NA | NA |
| 2013 | Maine | York | 53.4 | NA | NA | NA |
| 2013 | New York | Albany | 58.6 | 9.0 | 4.5 | NA |
| 2013 | New York | Allegany | 9.3 | 0.0 | 0.0 | NA |
| 2013 | New York | Cattaraugus | 38.0 | 0.0 | 0.0 | NA |
| 2013 | New York | Chautauqua | 28.3 | 0.0 | 0.0 | NA |
| 2013 | New York | Chemung | 45.5 | 0.0 | 0.0 | NA |
| 2013 | New York | Chenango | 0.0 | 0.0 | 0.0 | NA |
| 2013 | New York | Columbia | 68.0 | 2.0 | 10.0 | NA |
| 2013 | New York | Delaware | 0.0 | 0.0 | 0.0 | NA |
| 2013 | New York | Dutchess | 44.4 | 12.5 | 0.7 | NA |
| 2013 | New York | Erie | 60.8 | 0.0 | 0.0 | NA |
| 2013 | New York | Franklin | 37.5 | 0.0 | 0.0 | NA |
| 2013 | New York | Fulton | 0.0 | 0.0 | 0.0 | NA |
| 2013 | New York | Greene | 51.9 | 12.3 | 0.0 | NA |
| 2013 | New York | Hamilton | 100.0 | 0.0 | 0.0 | NA |
| 2013 | New York | Livingston | 7.7 | 0.0 | 0.0 | NA |
| 2013 | New York | Madison | 0.0 | 0.0 | 0.0 | NA |
| 2013 | New York | Monroe | 54.2 | 2.8 | 0.0 | NA |
| 2013 | New York | Montgomery | 57.1 | 0.0 | 0.0 | NA |
| 2013 | New York | Onondaga | 53.8 | 3.8 | 0.0 | NA |
| 2013 | New York | Orange | 46.0 | 8.0 | 0.0 | NA |
| 2013 | New York | Oswego | 32.7 | 0.0 | 0.0 | NA |
| 2013 | New York | Otsego | 25.0 | 0.0 | 0.0 | NA |
| 2013 | New York | Rensselaer | 42.5 | 2.5 | 0.0 | NA |
| 2013 | New York | Rockland | 32.7 | 1.9 | 0.0 | NA |
| 2013 | New York | Saratoga | 71.2 | 16.0 | 0.0 | NA |
| 2013 | New York | Schenectady | 62.2 | 0.0 | 0.0 | NA |
| 2013 | New York | Schoharie | 48.3 | 0.0 | 0.0 | NA |
| 2013 | New York | Schuyler | 48.7 | 0.0 | 0.0 | NA |
| 2013 | New York | Seneca | 58.0 | 0.0 | 0.0 | NA |
| 2013 | New York | Steuben | 9.1 | 0.0 | 0.0 | NA |
| 2013 | New York | Sullivan | 37.7 | 8.2 | 1.6 | NA |
| 2013 | New York | Tompkins | 9.1 | 0.0 | 0.0 | NA |
| 2013 | New York | Ulster | 56.0 | 6.0 | 0.0 | NA |
| 2013 | New York | Warren | 48.3 | 0.0 | 0.0 | NA |
| 2013 | New York | Washington | 41.2 | 4.6 | 0.0 | NA |
| 2013 | New York | Westchester | 20.0 | 6.0 | 2.0 | NA |
| 2013 | New York | Yates | 16.7 | 0.0 | 0.0 | NA |
| 2014 | Maine | Cumberland | 40.9 | NA | NA | NA |
| 2014 | Maine | Hancock | 25.8 | NA | NA | NA |
| 2014 | Maine | York | 45.5 | NA | NA | NA |
| 2014 | New York | Albany | 47.9 | 9.2 | 1.4 | NA |
| 2014 | New York | Allegany | 18.5 | 0.0 | 0.0 | NA |
| 2014 | New York | Broome | 52.3 | 0.0 | 0.0 | NA |
| 2014 | New York | Cattaraugus | 27.1 | 0.9 | 0.0 | NA |
| 2014 | New York | Chautauqua | 0.0 | 0.0 | 0.0 | NA |
| 2014 | New York | Chemung | 43.6 | 0.0 | 0.0 | NA |
| 2014 | New York | Chenango | 0.0 | 0.0 | 0.0 | NA |
| 2014 | New York | Clinton | 27.3 | 0.0 | 0.0 | NA |
| 2014 | New York | Columbia | 45.8 | 10.0 | 4.8 | NA |
| 2014 | New York | Delaware | 37.5 | 0.0 | 0.0 | NA |
| 2014 | New York | Dutchess | 51.4 | 5.7 | 7.1 | NA |
| 2014 | New York | Erie | 35.7 | 1.8 | 0.0 | NA |
| 2014 | New York | Essex | 10.3 | 0.0 | 0.0 | NA |
| 2014 | New York | Fulton | 63.0 | 0.0 | 0.0 | NA |
| 2014 | New York | Genesee | 0.0 | 0.0 | 0.0 | NA |
| 2014 | New York | Greene | 56.3 | 13.1 | 2.3 | NA |
| 2014 | New York | Jefferson | 48.0 | 3.3 | 0.0 | NA |
| 2014 | New York | Lewis | 100.0 | 0.0 | 0.0 | NA |
| 2014 | New York | Livingston | 18.2 | 9.1 | 0.0 | NA |
| 2014 | New York | Monroe | 69.5 | 5.7 | 0.0 | NA |
| 2014 | New York | Montgomery | 63.5 | 3.6 | 0.0 | NA |
| 2014 | New York | Onondaga | 51.7 | 0.0 | 0.0 | NA |
| 2014 | New York | Ontario | 50.0 | 0.0 | 0.0 | NA |
| 2014 | New York | Orange | 72.0 | 10.0 | 0.0 | NA |
| 2014 | New York | Oswego | 43.1 | 0.0 | 0.0 | NA |
| 2014 | New York | Otsego | 44.2 | 0.0 | 0.0 | NA |
| 2014 | New York | Rensselaer | 74.4 | 12.2 | 6.1 | NA |
| 2014 | New York | Rockland | 56.0 | 8.0 | 8.0 | NA |
| 2014 | New York | Saratoga | 72.0 | 6.0 | 3.0 | NA |
| 2014 | New York | Schenectady | 61.0 | 0.0 | 0.0 | NA |
| 2014 | New York | Schoharie | 55.7 | 1.0 | 0.0 | NA |
| 2014 | New York | Schuyler | 63.5 | 3.8 | 0.0 | NA |
| 2014 | New York | Seneca | 50.0 | 5.6 | 0.0 | NA |
| 2014 | New York | Steuben | 1.4 | 0.0 | 0.0 | NA |
| 2014 | New York | Suffolk | 55.0 | 18.0 | 22.5 | NA |
| 2014 | New York | Sullivan | 50.5 | 4.2 | 4.2 | NA |
| 2014 | New York | Tompkins | 61.1 | 5.6 | 0.0 | NA |
| 2014 | New York | Ulster | 55.9 | 4.4 | 4.4 | NA |
| 2014 | New York | Warren | 57.0 | 0.0 | 0.0 | NA |
| 2014 | New York | Washington | 76.0 | 5.4 | 1.6 | NA |
| 2014 | New York | Wayne | 50.0 | 0.0 | 0.0 | NA |
| 2014 | New York | Westchester | 32.0 | 6.0 | 12.0 | NA |
| 2014 | New York | Wyoming | 54.0 | 4.0 | 0.0 | NA |
| 2014 | New York | Yates | 33.3 | 0.0 | 0.0 | NA |
| 2015 | New York | Albany | 66.1 | 11.6 | 7.4 | 0.0 |
| 2015 | New York | Allegany | 26.8 | 0.0 | 0.0 | 0.0 |
| 2015 | New York | Cattaraugus | 39.9 | 2.9 | 0.0 | 0.0 |
| 2015 | New York | Chautauqua | 3.6 | 0.0 | 0.0 | 0.0 |
| 2015 | New York | Chemung | 42.4 | 0.0 | 0.0 | 0.0 |
| 2015 | New York | Clinton | 44.0 | 1.2 | 0.0 | 0.0 |
| 2015 | New York | Columbia | 56.9 | 13.8 | 9.2 | 2.3 |
| 2015 | New York | Delaware | 45.2 | 0.0 | 0.0 | 1.4 |
| 2015 | New York | Dutchess | 73.4 | 14.9 | 18.1 | 2.1 |
| 2015 | New York | Erie | 35.1 | 5.2 | 0.0 | 1.3 |
| 2015 | New York | Essex | 29.0 | 1.4 | 0.0 | 0.0 |
| 2015 | New York | Franklin | 38.5 | 0.0 | 0.0 | 0.0 |
| 2015 | New York | Fulton | 65.2 | 2.2 | 3.4 | 0.0 |
| 2015 | New York | Genesee | 7.1 | 0.0 | 0.0 | 0.0 |
| 2015 | New York | Greene | 62.8 | 14.0 | 11.6 | 2.3 |
| 2015 | New York | Hamilton | 0.0 | 0.0 | 0.0 | 0.0 |
| 2015 | New York | Herkimer | 12.5 | 0.0 | 0.0 | 0.0 |
| 2015 | New York | Jefferson | 53.2 | 3.2 | 0.0 | 3.2 |
| 2015 | New York | Livingston | 20.7 | 0.0 | 0.0 | 0.0 |
| 2015 | New York | Madison | 0.0 | 0.0 | 0.0 | 0.0 |
| 2015 | New York | Monroe | 70.4 | 2.8 | 0.9 | 1.9 |
| 2015 | New York | Montgomery | 52.6 | 2.6 | 0.0 | 0.6 |
| 2015 | New York | Niagara | 0.0 | 0.0 | 0.0 | 0.0 |
| 2015 | New York | Oneida | 100.0 | 0.0 | 0.0 | 0.0 |
| 2015 | New York | Onondaga | 42.7 | 0.3 | 0.0 | 0.6 |
| 2015 | New York | Ontario | 51.9 | 1.9 | 0.0 | 0.0 |
| 2015 | New York | Orange | 50.0 | 10.0 | 0.0 | 0.0 |
| 2015 | New York | Orleans | 16.7 | 0.0 | 0.0 | 0.0 |
| 2015 | New York | Oswego | 50.0 | 44.1 | 0.0 | 0.0 |
| 2015 | New York | Otsego | 56.9 | 1.5 | 3.1 | 0.0 |
| 2015 | New York | Rensselaer | 64.2 | 9.5 | 7.4 | 2.1 |
| 2015 | New York | Rockland | 64.0 | 18.0 | 10.0 | 4.0 |
| 2015 | New York | Saratoga | 66.0 | 16.0 | 1.3 | 0.7 |
| 2015 | New York | Schenectady | 54.7 | 3.2 | 0.0 | 1.1 |
| 2015 | New York | Schoharie | 58.5 | 0.0 | 3.2 | 1.1 |
| 2015 | New York | Schuyler | 56.9 | 1.0 | 0.0 | 1.0 |
| 2015 | New York | Seneca | 55.8 | 9.6 | 0.0 | 3.8 |
| 2015 | New York | St. Lawrence | 52.4 | 1.4 | 0.0 | 1.7 |
| 2015 | New York | Steuben | 14.3 | 0.0 | 0.0 | 0.0 |
| 2015 | New York | Suffolk | 70.0 | 20.0 | 20.0 | 5.0 |
| 2015 | New York | Sullivan | 53.7 | 14.0 | 11.8 | 3.7 |
| 2015 | New York | Tioga | 0.0 | 0.0 | 0.0 | 0.0 |
| 2015 | New York | Ulster | 60.0 | 9.4 | 4.7 | 0.0 |
| 2015 | New York | Warren | 49.4 | 2.5 | 3.8 | 1.3 |
| 2015 | New York | Washington | 62.7 | 8.5 | 5.2 | 0.7 |
| 2015 | New York | Wayne | 7.7 | 0.0 | 0.0 | 0.0 |
| 2015 | New York | Westchester | 52.0 | 14.0 | 10.0 | 10.0 |
| 2015 | New York | Wyoming | 39.3 | 5.4 | 0.0 | 0.0 |
| 2015 | New York | Yates | 38.2 | 1.5 | 0.0 | 8.8 |
| 2015 | Vermont | Bennington | 55.9 | 18.4 | 2.0 | NA |
| 2015 | Vermont | Chittenden | 55.2 | 0.0 | 0.0 | NA |
| 2015 | Vermont | Franklin | 66.7 | 0.0 | 0.0 | NA |
| 2015 | Vermont | Grand Isle | 66.7 | 0.0 | 0.0 | NA |
| 2015 | Vermont | Windham | 46.7 | 6.7 | 0.0 | NA |
| 2015 | Vermont | Windsor | 64.6 | 8.7 | 0.0 | NA |
| 2016 | New York | Albany | 65.8 | 20.2 | 12.3 | 2.6 |
| 2016 | New York | Allegany | 55.4 | 1.8 | 0.0 | 0.0 |
| 2016 | New York | Broome | 78.0 | 0.0 | 0.0 | 0.0 |
| 2016 | New York | Cattaraugus | 49.3 | 1.8 | 0.5 | 0.5 |
| 2016 | New York | Cayuga | 46.4 | 1.8 | 0.0 | 1.8 |
| 2016 | New York | Chautauqua | 24.0 | 2.0 | 0.0 | 2.0 |
| 2016 | New York | Chemung | 54.0 | 6.0 | 0.0 | 2.0 |
| 2016 | New York | Chenango | 54.9 | 3.9 | 0.0 | 0.0 |
| 2016 | New York | Clinton | 49.1 | 0.9 | 1.9 | 0.9 |
| 2016 | New York | Columbia | 61.3 | 15.3 | 12.4 | 3.6 |
| 2016 | New York | Cortland | 70.6 | 0.0 | 0.0 | 0.0 |
| 2016 | New York | Delaware | 62.5 | 3.1 | 0.0 | 1.6 |
| 2016 | New York | Dutchess | 63.6 | 14.6 | 17.7 | 0.5 |
| 2016 | New York | Erie | 30.0 | 0.0 | 0.0 | 0.0 |
| 2016 | New York | Essex | 58.0 | 4.0 | 12.0 | 0.0 |
| 2016 | New York | Franklin | 46.0 | 0.0 | 0.0 | 0.0 |
| 2016 | New York | Fulton | 58.8 | 0.0 | 0.0 | 2.9 |
| 2016 | New York | Genesee | 0.0 | 0.0 | 0.0 | 0.0 |
| 2016 | New York | Greene | 46.0 | 10.0 | 24.0 | 2.0 |
| 2016 | New York | Hamilton | 0.0 | 0.0 | 0.0 | 0.0 |
| 2016 | New York | Lewis | 60.7 | 3.6 | 0.0 | 0.0 |
| 2016 | New York | Livingston | 46.4 | 0.9 | 1.8 | 0.0 |
| 2016 | New York | Madison | 41.4 | 0.0 | 0.0 | 0.0 |
| 2016 | New York | Monroe | 56.8 | 3.2 | 0.2 | 1.4 |
| 2016 | New York | Montgomery | 58.0 | 6.3 | 0.9 | 0.0 |
| 2016 | New York | Niagara | 0.0 | 0.0 | 0.0 | 0.0 |
| 2016 | New York | Oneida | 52.0 | 0.0 | 0.0 | 0.0 |
| 2016 | New York | Onondaga | 48.8 | 0.7 | 0.0 | 0.4 |
| 2016 | New York | Ontario | 66.7 | 1.5 | 0.0 | 0.0 |
| 2016 | New York | Orange | 64.0 | 22.0 | 8.0 | 4.0 |
| 2016 | New York | Orleans | 50.0 | 0.0 | 0.0 | 0.0 |
| 2016 | New York | Oswego | 50.0 | 0.0 | 0.0 | 1.9 |
| 2016 | New York | Otsego | 66.7 | 1.4 | 1.4 | 0.0 |
| 2016 | New York | Rensselaer | 51.9 | 15.6 | 3.9 | 2.6 |
| 2016 | New York | Rockland | 76.0 | 28.0 | 22.0 | 4.0 |
| 2016 | New York | Saratoga | 57.4 | 15.5 | 6.1 | 1.4 |
| 2016 | New York | Schenectady | 52.6 | 1.8 | 8.8 | 0.0 |
| 2016 | New York | Schoharie | 77.3 | 2.1 | 3.1 | 0.0 |
| 2016 | New York | Schuyler | 67.3 | 0.0 | 0.0 | 0.0 |
| 2016 | New York | St. Lawrence | 59.0 | 3.0 | 0.0 | 0.0 |
| 2016 | New York | Steuben | 35.9 | 1.1 | 1.1 | 0.0 |
| 2016 | New York | Suffolk | 58.9 | 21.0 | 10.3 | 2.6 |
| 2016 | New York | Sullivan | 61.3 | 15.7 | 10.5 | 0.5 |
| 2016 | New York | Tompkins | 58.7 | 0.0 | 0.0 | 0.0 |
| 2016 | New York | Ulster | 64.1 | 23.3 | 14.6 | 0.0 |
| 2016 | New York | Warren | 57.1 | 8.9 | 0.0 | 5.4 |
| 2016 | New York | Washington | 52.0 | 13.3 | 0.7 | 2.0 |
| 2016 | New York | Westchester | 56.0 | 2.0 | 32.0 | 4.0 |
| 2016 | New York | Wyoming | 36.5 | 0.0 | 0.0 | 0.0 |
| 2016 | Vermont | Addison | 70.0 | 10.0 | 0.0 | 0.0 |
| 2016 | Vermont | Bennington | 0.0 | 0.0 | 0.0 | 0.0 |
| 2016 | Vermont | Chittenden | 52.4 | 0.0 | 0.0 | 0.0 |
| 2016 | Vermont | Essex | 0.0 | 0.0 | 0.0 | 0.0 |
| 2016 | Vermont | Franklin | 0.0 | 0.0 | 0.0 | 0.0 |
| 2016 | Vermont | Grand Isle | 0.0 | 0.0 | 0.0 | 0.0 |
| 2016 | Vermont | Orange | 0.0 | 0.0 | 0.0 | 0.0 |
| 2016 | Vermont | Rutland | 50.0 | 19.4 | 5.6 | 0.0 |
| 2016 | Vermont | Washington | 0.0 | 0.0 | 0.0 | 0.0 |
| 2016 | Vermont | Windham | 38.5 | 0.0 | 7.7 | 0.0 |
| 2016 | Vermont | Windsor | 75.0 | 12.5 | 0.0 | 0.0 |
| 2017 | New York | Albany | 64.0 | 15.1 | 11.5 | 1.4 |
| 2017 | New York | Allegany | 45.5 | 0.0 | 0.0 | 0.0 |
| 2017 | New York | Cattaraugus | 42.6 | 2.5 | 0.0 | 0.0 |
| 2017 | New York | Chautauqua | 42.6 | 1.0 | 0.0 | 0.0 |
| 2017 | New York | Chemung | 46.0 | 10.0 | 0.0 | 2.0 |
| 2017 | New York | Clinton | 37.3 | 3.9 | 1.0 | 0.0 |
| 2017 | New York | Columbia | 45.3 | 10.7 | 2.7 | 4.7 |
| 2017 | New York | Delaware | 58.5 | 1.2 | 0.0 | 0.0 |
| 2017 | New York | Dutchess | 32.0 | 8.0 | 2.0 | 4.0 |
| 2017 | New York | Erie | 52.0 | 6.0 | 0.0 | 0.0 |
| 2017 | New York | Essex | 50.3 | 1.7 | 4.5 | 0.6 |
| 2017 | New York | Franklin | 42.6 | 5.6 | 1.9 | 0.0 |
| 2017 | New York | Fulton | 41.9 | 0.0 | 0.0 | 1.6 |
| 2017 | New York | Genesee | 25.0 | 0.0 | 0.0 | 0.0 |
| 2017 | New York | Greene | 57.4 | 14.9 | 19.1 | 6.4 |
| 2017 | New York | Hamilton | 100.0 | 0.0 | 0.0 | 0.0 |
| 2017 | New York | Herkimer | 55.1 | 10.2 | 0.0 | 0.0 |
| 2017 | New York | Livingston | 49.0 | 4.0 | 0.0 | 1.0 |
| 2017 | New York | Madison | 52.9 | 0.0 | 0.0 | 2.0 |
| 2017 | New York | Monroe | 50.0 | 3.0 | 1.0 | 0.0 |
| 2017 | New York | Montgomery | 46.3 | 3.0 | 0.0 | 0.0 |
| 2017 | New York | Niagara | 50.0 | 1.7 | 8.3 | 0.0 |
| 2017 | New York | Onondaga | 46.2 | 5.8 | 0.0 | 3.8 |
| 2017 | New York | Ontario | 62.0 | 0.0 | 0.0 | 0.0 |
| 2017 | New York | Orange | 82.0 | 16.0 | 24.0 | 2.0 |
| 2017 | New York | Orleans | 58.0 | 0.0 | 0.0 | 0.0 |
| 2017 | New York | Oswego | 41.2 | 0.0 | 0.0 | 3.9 |
| 2017 | New York | Otsego | 41.4 | 1.7 | 0.0 | 3.4 |
| 2017 | New York | Rensselaer | 42.3 | 16.2 | 9.9 | 3.6 |
| 2017 | New York | Rockland | 58.0 | 18.0 | 24.0 | 0.0 |
| 2017 | New York | Saratoga | 58.0 | 14.5 | 15.5 | 1.0 |
| 2017 | New York | Schenectady | 49.4 | 8.9 | 6.3 | 0.0 |
| 2017 | New York | Schoharie | 58.9 | 11.0 | 9.6 | 1.4 |
| 2017 | New York | Schuyler | 56.4 | 0.0 | 0.0 | 2.0 |
| 2017 | New York | Seneca | 52.9 | 7.8 | 0.0 | 3.9 |
| 2017 | New York | St. Lawrence | 50.9 | 13.2 | 1.9 | 0.0 |
| 2017 | New York | Steuben | 57.9 | 1.9 | 0.0 | 0.0 |
| 2017 | New York | Suffolk | 60.6 | 14.9 | 18.1 | 1.9 |
| 2017 | New York | Sullivan | 56.3 | 9.4 | 7.0 | 0.0 |
| 2017 | New York | Tioga | 0.0 | 0.0 | 0.0 | 0.0 |
| 2017 | New York | Tompkins | 49.5 | 8.4 | 0.0 | 2.1 |
| 2017 | New York | Ulster | 42.2 | 3.0 | 11.1 | 2.2 |
| 2017 | New York | Warren | 61.5 | 13.5 | 1.9 | 0.0 |
| 2017 | New York | Washington | 43.3 | 3.8 | 9.6 | 0.0 |
| 2017 | New York | Westchester | 34.0 | 6.0 | 6.0 | 2.0 |
| 2017 | New York | Wyoming | 22.0 | 4.0 | 0.0 | 0.0 |
| 2017 | New York | Yates | 50.0 | 4.0 | 0.0 | 0.0 |
| 2017 | Vermont | Addison | 57.1 | 0.0 | 0.0 | 0.0 |
| 2017 | Vermont | Bennington | 20.0 | 13.3 | 0.0 | 0.0 |
| 2017 | Vermont | Caledonia | 47.4 | 5.3 | 0.0 | 0.0 |
| 2017 | Vermont | Chittenden | 100.0 | 0.0 | 0.0 | 0.0 |
| 2017 | Vermont | Essex | 0.0 | 0.0 | 0.0 | 0.0 |
| 2017 | Vermont | Franklin | 60.0 | 0.0 | 0.0 | 0.0 |
| 2017 | Vermont | Lamoille | 33.3 | 0.0 | 0.0 | 0.0 |
| 2017 | Vermont | Orange | 33.3 | 0.0 | 0.0 | 0.0 |
| 2017 | Vermont | Rutland | 43.0 | 6.0 | 4.0 | 2.0 |
| 2017 | Vermont | Washington | 0.0 | 0.0 | 0.0 | 0.0 |
| 2017 | Vermont | Windham | 52.9 | 17.6 | 5.9 | 0.0 |
| 2017 | Vermont | Windsor | 47.4 | 15.8 | 5.3 | 0.0 |
| 2018 | New York | Albany | 64.2 | 14.8 | 8.6 | 1.2 |
| 2018 | New York | Allegany | 60.0 | 0.0 | 0.0 | 2.0 |
| 2018 | New York | Cattaraugus | 50.4 | 3.6 | 0.0 | 0.0 |
| 2018 | New York | Chautauqua | 57.0 | 1.0 | 0.0 | 0.0 |
| 2018 | New York | Chemung | 62.0 | 28.0 | 0.0 | 0.0 |
| 2018 | New York | Chenango | 46.0 | 4.0 | 0.0 | 0.0 |
| 2018 | New York | Clinton | 57.1 | 3.1 | 0.0 | 2.0 |
| 2018 | New York | Columbia | 59.8 | 17.2 | 9.5 | 3.6 |
| 2018 | New York | Delaware | 53.8 | 7.7 | 0.0 | 0.0 |
| 2018 | New York | Dutchess | 54.0 | 4.0 | 24.0 | 2.0 |
| 2018 | New York | Erie | 50.0 | 1.6 | 1.6 | 0.0 |
| 2018 | New York | Essex | 60.6 | 9.1 | 11.1 | 1.0 |
| 2018 | New York | Franklin | 56.0 | 10.0 | 0.0 | 0.0 |
| 2018 | New York | Fulton | 56.0 | 4.0 | 0.0 | 2.0 |
| 2018 | New York | Greene | 57.1 | 20.2 | 6.7 | 0.8 |
| 2018 | New York | Jefferson | 72.0 | 0.0 | 0.0 | 0.0 |
| 2018 | New York | Livingston | 71.7 | 1.0 | 0.0 | 1.0 |
| 2018 | New York | Monroe | 60.0 | 4.0 | 0.0 | 2.0 |
| 2018 | New York | Montgomery | 59.8 | 8.4 | 0.0 | 0.9 |
| 2018 | New York | Niagara | 58.0 | 4.0 | 0.0 | 2.0 |
| 2018 | New York | Onondaga | 60.0 | 4.0 | 0.0 | 0.0 |
| 2018 | New York | Ontario | 78.0 | 0.0 | 0.0 | 0.0 |
| 2018 | New York | Orange | 52.0 | 16.0 | 8.0 | 2.0 |
| 2018 | New York | Oswego | 70.0 | 0.0 | 0.0 | 0.0 |
| 2018 | New York | Otsego | 71.6 | 1.2 | 0.0 | 1.2 |
| 2018 | New York | Rensselaer | 53.6 | 14.3 | 10.7 | 1.8 |
| 2018 | New York | Rockland | 64.0 | 22.0 | 10.0 | 4.0 |
| 2018 | New York | Saratoga | 57.8 | 20.6 | 14.7 | 3.9 |
| 2018 | New York | Schenectady | 67.0 | 6.0 | 8.0 | 1.0 |
| 2018 | New York | Schoharie | 62.0 | 15.2 | 13.9 | 0.0 |
| 2018 | New York | Schuyler | 72.9 | 1.4 | 0.0 | 2.9 |
| 2018 | New York | Seneca | 62.0 | 6.0 | 2.0 | 4.0 |
| 2018 | New York | St. Lawrence | 44.0 | 7.0 | 0.0 | 0.0 |
| 2018 | New York | Steuben | 50.0 | 1.7 | 0.0 | 0.9 |
| 2018 | New York | Suffolk | 46.7 | 13.0 | 11.0 | 4.5 |
| 2018 | New York | Sullivan | 62.0 | 26.0 | 20.0 | 0.0 |
| 2018 | New York | Tioga | 58.0 | 18.0 | 8.0 | 4.0 |
| 2018 | New York | Tompkins | 76.5 | 3.9 | 0.0 | 0.0 |
| 2018 | New York | Ulster | 56.0 | 6.0 | 8.0 | 2.0 |
| 2018 | New York | Warren | 60.8 | 9.5 | 2.7 | 2.7 |
| 2018 | New York | Washington | 65.8 | 11.0 | 11.0 | 5.5 |
| 2018 | New York | Westchester | 44.4 | 8.9 | 10.0 | 5.6 |
| 2018 | New York | Yates | 64.0 | 0.0 | 0.0 | 4.0 |
| 2018 | Vermont | Addison | 50.0 | 0.0 | 0.0 | 0.0 |
| 2018 | Vermont | Rutland | 60.0 | 14.0 | 0.0 | 2.0 |
| 2018 | Vermont | Windham | 100.0 | 0.0 | 0.0 | 0.0 |
| 2018 | Vermont | Windsor | 100.0 | 0.0 | 0.0 | 0.0 |
| 2019 | Connecticut | Fairfield | 61.0 | 14.4 | 19.5 | 3.4 |
| 2019 | Connecticut | Hartford | 40.0 | 4.4 | 6.7 | 0.0 |
| 2019 | Connecticut | Litchfield | 41.0 | 10.3 | 17.1 | 0.0 |
| 2019 | Connecticut | Middlesex | 52.2 | 0.0 | 21.7 | 8.7 |
| 2019 | Connecticut | New Haven | 35.2 | 10.2 | 11.1 | 3.7 |
| 2019 | Connecticut | New London | 54.7 | 10.4 | 12.3 | 0.9 |
| 2019 | Connecticut | Tolland | 47.1 | 4.4 | 7.4 | 1.5 |
| 2019 | Connecticut | Windham | 33.3 | 3.5 | 5.3 | 0.0 |
| 2019 | New York | Albany | 61.7 | 13.9 | 13.9 | 0.9 |
| 2019 | New York | Allegany | 40.0 | 0.0 | 2.0 | 4.0 |
| 2019 | New York | Broome | 47.4 | 10.5 | 0.0 | 0.0 |
| 2019 | New York | Cattaraugus | 53.6 | 6.0 | 0.0 | 0.0 |
| 2019 | New York | Cayuga | 54.0 | 0.0 | 0.0 | 0.0 |
| 2019 | New York | Chautauqua | 57.0 | 0.0 | 0.0 | 0.0 |
| 2019 | New York | Clinton | 53.3 | 5.6 | 9.3 | 2.8 |
| 2019 | New York | Columbia | 54.5 | 13.2 | 10.1 | 0.5 |
| 2019 | New York | Delaware | 47.2 | 5.5 | 5.5 | 0.0 |
| 2019 | New York | Dutchess | 48.0 | 10.0 | 6.0 | 6.0 |
| 2019 | New York | Erie | 45.1 | 0.0 | 7.8 | 2.0 |
| 2019 | New York | Essex | 53.9 | 11.1 | 4.3 | 0.9 |
| 2019 | New York | Franklin | 42.3 | 5.6 | 0.0 | 0.0 |
| 2019 | New York | Fulton | 60.9 | 4.3 | 0.9 | 0.9 |
| 2019 | New York | Genesee | 75.9 | 0.0 | 0.0 | 0.0 |
| 2019 | New York | Greene | 61.0 | 12.6 | 3.8 | 1.1 |
| 2019 | New York | Hamilton | 50.0 | 0.0 | 0.0 | 0.0 |
| 2019 | New York | Jefferson | 78.0 | 2.0 | 0.0 | 0.0 |
| 2019 | New York | Livingston | 70.0 | 0.0 | 0.0 | 0.0 |
| 2019 | New York | Monroe | 43.0 | 14.0 | 0.0 | 0.0 |
| 2019 | New York | Montgomery | 54.3 | 19.9 | 4.0 | 2.0 |
| 2019 | New York | Niagara | 48.0 | 0.0 | 0.0 | 0.0 |
| 2019 | New York | Oneida | 56.0 | 0.0 | 0.0 | 0.0 |
| 2019 | New York | Onondaga | 50.0 | 18.0 | 0.0 | 0.0 |
| 2019 | New York | Ontario | 60.0 | 4.0 | 0.0 | 0.0 |
| 2019 | New York | Orange | 44.0 | 26.0 | 6.0 | 0.0 |
| 2019 | New York | Orleans | 56.3 | 0.0 | 0.0 | 0.0 |
| 2019 | New York | Oswego | 70.0 | 0.0 | 0.0 | 0.0 |
| 2019 | New York | Otsego | 65.0 | 2.6 | 4.3 | 0.0 |
| 2019 | New York | Rensselaer | 50.0 | 14.1 | 4.3 | 3.0 |
| 2019 | New York | Rockland | 76.0 | 12.0 | 26.0 | 0.0 |
| 2019 | New York | Saratoga | 53.3 | 10.4 | 11.6 | 0.7 |
| 2019 | New York | Schenectady | 60.7 | 14.2 | 7.8 | 0.5 |
| 2019 | New York | Schoharie | 48.2 | 14.3 | 0.9 | 0.0 |
| 2019 | New York | St. Lawrence | 42.0 | 2.0 | 0.0 | 2.0 |
| 2019 | New York | Suffolk | 42.5 | 11.7 | 4.2 | 2.6 |
| 2019 | New York | Sullivan | 52.0 | 16.0 | 0.0 | 2.0 |
| 2019 | New York | Tompkins | 52.0 | 16.0 | 0.0 | 2.0 |
| 2019 | New York | Ulster | 44.0 | 6.0 | 8.0 | 0.0 |
| 2019 | New York | Warren | 55.4 | 16.1 | 0.0 | 0.0 |
| 2019 | New York | Washington | 38.3 | 8.1 | 11.4 | 0.7 |
| 2019 | New York | Westchester | 56.0 | 6.0 | 4.0 | 0.0 |
| 2019 | New York | Wyoming | 36.7 | 10.0 | 0.0 | 0.0 |
| 2019 | Vermont | Addison | 59.3 | 11.9 | 15.3 | 0.0 |
| 2019 | Vermont | Chittenden | 65.2 | 2.2 | 0.0 | 0.0 |
| 2019 | Vermont | Orleans | 0.0 | 0.0 | 0.0 | 0.0 |
| 2019 | Vermont | Rutland | 50.0 | 6.3 | 12.5 | 6.3 |
| 2019 | Vermont | Washington | 55.6 | 0.0 | 0.0 | 0.0 |
| 2020 | Connecticut | Fairfield | 60.7 | 10.7 | 7.1 | 3.6 |
| 2020 | Connecticut | Hartford | 48.2 | 8.9 | 10.7 | 5.4 |
| 2020 | Connecticut | Litchfield | 52.6 | 7.9 | 7.9 | 2.6 |
| 2020 | Connecticut | Middlesex | 55.0 | 5.0 | 10.0 | 0.0 |
| 2020 | Connecticut | New Haven | 55.0 | 15.0 | 10.0 | 5.0 |
| 2020 | Connecticut | New London | 40.6 | 5.8 | 7.2 | 0.0 |
| 2020 | Connecticut | Tolland | 37.7 | 11.3 | 5.7 | 0.0 |
| 2020 | Connecticut | Windham | 50.5 | 3.8 | 9.5 | 2.9 |
| 2020 | New York | Albany | 71.8 | 13.6 | 8.7 | 0.0 |
| 2020 | New York | Allegany | 66.0 | 22.0 | 0.0 | 4.0 |
| 2020 | New York | Cattaraugus | 56.5 | 7.7 | 0.0 | 0.0 |
| 2020 | New York | Cayuga | 60.0 | 0.0 | 0.0 | 0.0 |
| 2020 | New York | Chautauqua | 65.0 | 0.0 | 0.0 | 0.0 |
| 2020 | New York | Chemung | 56.0 | 16.0 | 0.0 | 2.0 |
| 2020 | New York | Chenango | 80.0 | 0.0 | 0.0 | 0.0 |
| 2020 | New York | Clinton | 58.8 | 8.8 | 6.9 | 0.0 |
| 2020 | New York | Columbia | 61.5 | 10.0 | 10.5 | 1.5 |
| 2020 | New York | Cortland | 52.9 | 0.0 | 3.9 | 2.0 |
| 2020 | New York | Delaware | 68.1 | 10.1 | 0.0 | 1.4 |
| 2020 | New York | Dutchess | 80.0 | 30.0 | 28.0 | 2.0 |
| 2020 | New York | Erie | 54.9 | 4.4 | 0.0 | 0.0 |
| 2020 | New York | Essex | 63.9 | 20.4 | 14.8 | 0.9 |
| 2020 | New York | Franklin | 60.0 | 8.0 | 0.0 | 0.0 |
| 2020 | New York | Fulton | 55.0 | 12.0 | 5.0 | 2.0 |
| 2020 | New York | Genesee | 32.0 | 0.0 | 0.0 | 0.0 |
| 2020 | New York | Greene | 59.8 | 13.4 | 16.1 | 0.9 |
| 2020 | New York | Hamilton | 0.0 | 0.0 | 0.0 | 0.0 |
| 2020 | New York | Jefferson | 76.7 | 3.3 | 0.0 | 3.3 |
| 2020 | New York | Livingston | 50.0 | 4.0 | 0.0 | 0.0 |
| 2020 | New York | Monroe | 53.0 | 16.0 | 0.0 | 1.0 |
| 2020 | New York | Montgomery | 62.1 | 20.5 | 4.5 | 0.8 |
| 2020 | New York | Niagara | 45.5 | 0.0 | 0.0 | 0.0 |
| 2020 | New York | Oneida | 48.0 | 0.0 | 0.0 | 0.0 |
| 2020 | New York | Onondaga | 48.0 | 24.0 | 0.0 | 0.0 |
| 2020 | New York | Ontario | 66.0 | 2.0 | 0.0 | 2.0 |
| 2020 | New York | Orange | 60.0 | 8.0 | 10.0 | 2.0 |
| 2020 | New York | Orleans | 25.0 | 0.0 | 0.0 | 0.0 |
| 2020 | New York | Oswego | 50.0 | 0.0 | 0.0 | 0.0 |
| 2020 | New York | Otsego | 63.6 | 6.1 | 7.6 | 1.5 |
| 2020 | New York | Rensselaer | 57.8 | 15.6 | 6.3 | 1.6 |
| 2020 | New York | Rockland | 34.0 | 22.0 | 18.0 | 4.0 |
| 2020 | New York | Saratoga | 61.4 | 14.2 | 7.4 | 1.1 |
| 2020 | New York | Schenectady | 55.8 | 12.8 | 7.0 | 1.2 |
| 2020 | New York | Schoharie | 63.6 | 20.2 | 9.3 | 0.0 |
| 2020 | New York | Schuyler | 66.0 | 0.0 | 0.0 | 0.0 |
| 2020 | New York | Seneca | 46.0 | 6.0 | 0.0 | 0.0 |
| 2020 | New York | St. Lawrence | 48.0 | 12.0 | 0.0 | 2.0 |
| 2020 | New York | Steuben | 62.0 | 2.0 | 0.0 | 1.0 |
| 2020 | New York | Suffolk | 51.4 | 16.0 | 9.9 | 2.6 |
| 2020 | New York | Sullivan | 50.0 | 22.0 | 10.0 | 0.0 |
| 2020 | New York | Tompkins | 59.1 | 36.4 | 0.0 | 2.3 |
| 2020 | New York | Ulster | 62.0 | 8.0 | 10.0 | 2.0 |
| 2020 | New York | Warren | 51.3 | 10.0 | 5.0 | 1.3 |
| 2020 | New York | Washington | 67.6 | 17.6 | 7.4 | 1.4 |
| 2020 | New York | Wayne | 60.0 | 4.0 | 0.0 | 0.0 |
| 2020 | New York | Westchester | 62.0 | 14.0 | 12.0 | 4.0 |
| 2020 | New York | Wyoming | 46.0 | 4.0 | 0.0 | 0.0 |
| 2020 | New York | Yates | 20.0 | 0.0 | 0.0 | 0.0 |
| 2021 | Connecticut | Fairfield | 50.0 | 9.5 | 16.7 | 4.8 |
| 2021 | Connecticut | Hartford | 69.8 | 20.8 | 15.1 | 0.0 |
| 2021 | Connecticut | Litchfield | 45.3 | 7.5 | 20.8 | 0.0 |
| 2021 | Connecticut | Middlesex | 43.8 | 6.3 | 28.1 | 6.3 |
| 2021 | Connecticut | New Haven | 45.5 | 6.1 | 18.2 | 0.0 |
| 2021 | Connecticut | New London | 55.1 | 15.8 | 25.3 | 2.5 |
| 2021 | Connecticut | Tolland | 60.4 | 9.4 | 3.8 | 0.0 |
| 2021 | Connecticut | Windham | 45.3 | 15.1 | 5.8 | 1.2 |
| 2021 | New York | Albany | 53.6 | 12.5 | 9.5 | 2.4 |
| 2021 | New York | Allegany | 60.0 | 26.0 | 0.0 | 0.0 |
| 2021 | New York | Broome | 66.7 | 8.3 | 0.0 | 0.0 |
| 2021 | New York | Cattaraugus | 53.2 | 10.8 | 0.4 | 0.8 |
| 2021 | New York | Cayuga | 56.0 | 6.0 | 0.0 | 0.0 |
| 2021 | New York | Chautauqua | 56.1 | 0.0 | 0.0 | 0.0 |
| 2021 | New York | Chenango | 53.9 | 1.3 | 0.0 | 0.0 |
| 2021 | New York | Clinton | 69.2 | 14.4 | 15.4 | 0.0 |
| 2021 | New York | Columbia | 53.8 | 13.5 | 7.2 | 2.9 |
| 2021 | New York | Delaware | 51.4 | 12.3 | 2.7 | 0.7 |
| 2021 | New York | Dutchess | 47.1 | 11.8 | 11.8 | 5.9 |
| 2021 | New York | Erie | 40.0 | 0.0 | 0.0 | 0.0 |
| 2021 | New York | Franklin | 38.0 | 0.0 | 2.0 | 0.0 |
| 2021 | New York | Fulton | 55.8 | 11.6 | 2.3 | 3.5 |
| 2021 | New York | Genesee | 43.8 | 6.3 | 0.0 | 0.0 |
| 2021 | New York | Greene | 56.0 | 13.5 | 9.2 | 1.0 |
| 2021 | New York | Hamilton | 25.0 | 0.0 | 0.0 | 0.0 |
| 2021 | New York | Herkimer | 38.0 | 26.0 | 4.0 | 2.0 |
| 2021 | New York | Jefferson | 78.0 | 2.0 | 0.0 | 2.0 |
| 2021 | New York | Madison | 50.0 | 2.0 | 0.0 | 0.0 |
| 2021 | New York | Monroe | 55.9 | 15.8 | 0.0 | 1.7 |
| 2021 | New York | Montgomery | 48.0 | 19.3 | 6.7 | 2.0 |
| 2021 | New York | Oneida | 54.0 | 19.6 | 13.7 | 3.9 |
| 2021 | New York | Onondaga | 41.6 | 5.9 | 1.0 | 2.0 |
| 2021 | New York | Ontario | 64.3 | 11.9 | 0.0 | 0.0 |
| 2021 | New York | Orange | 42.0 | 22.0 | 10.0 | 4.0 |
| 2021 | New York | Orleans | 42.1 | 0.0 | 0.0 | 0.0 |
| 2021 | New York | Oswego | 49.5 | 3.0 | 5.1 | 3.0 |
| 2021 | New York | Otsego | 56.7 | 15.6 | 4.4 | 0.0 |
| 2021 | New York | Rensselaer | 61.0 | 13.9 | 10.7 | 1.1 |
| 2021 | New York | Rockland | 28.0 | 2.0 | 2.0 | 2.0 |
| 2021 | New York | Saratoga | 65.5 | 21.3 | 16.1 | 0.0 |
| 2021 | New York | Schenectady | 51.2 | 21.5 | 12.4 | 0.8 |
| 2021 | New York | Schoharie | 52.4 | 15.1 | 11.9 | 1.6 |
| 2021 | New York | Schuyler | 56.0 | 12.0 | 14.0 | 0.0 |
| 2021 | New York | Seneca | 56.0 | 6.0 | 0.0 | 0.0 |
| 2021 | New York | St. Lawrence | 46.0 | 16.0 | 0.0 | 0.0 |
| 2021 | New York | Steuben | 66.0 | 0.0 | 0.0 | 0.0 |
| 2021 | New York | Suffolk | 58.4 | 12.6 | 11.6 | 4.0 |
| 2021 | New York | Sullivan | 54.0 | 16.0 | 4.0 | 0.0 |
| 2021 | New York | Tompkins | 46.0 | 14.0 | 0.0 | 0.0 |
| 2021 | New York | Ulster | 64.7 | 11.8 | 3.9 | 2.0 |
| 2021 | New York | Warren | 41.4 | 6.9 | 9.2 | 2.3 |
| 2021 | New York | Washington | 59.0 | 14.1 | 8.3 | 2.0 |
| 2021 | New York | Westchester | 26.0 | 12.0 | 6.0 | 2.0 |

Table S5. County centroid latitudes in decimal degrees from North American Datum of 1983 for all counties within Connecticut, Maine, New Hampshire, New York, and Vermont where *Ixodes scapularis* were collected.

| State | County | Latitude |
| --- | --- | --- |
| Connecticut | Fairfield | 41.27 |
| Connecticut | Hartford | 41.81 |
| Connecticut | Litchfield | 41.79 |
| Connecticut | Middlesex | 41.47 |
| Connecticut | New Haven | 41.41 |
| Connecticut | New London | 41.48 |
| Connecticut | Tolland | 41.85 |
| Connecticut | Windham | 41.83 |
| Maine | Androscoggin | 44.17 |
| Maine | Aroostook | 46.66 |
| Maine | Cumberland | 43.85 |
| Maine | Franklin | 44.97 |
| Maine | Hancock | 44.70 |
| Maine | Kennebec | 44.41 |
| Maine | Knox | 44.17 |
| Maine | Lincoln | 44.09 |
| Maine | Oxford | 44.50 |
| Maine | Penobscot | 45.40 |
| Maine | Piscataquis | 45.84 |
| Maine | Sagadahoc | 43.99 |
| Maine | Somerset | 45.51 |
| Maine | Waldo | 44.51 |
| Maine | Washington | 45.05 |
| Maine | York | 43.49 |
| New Hampshire | Belknap | 43.51 |
| New Hampshire | Carroll | 43.87 |
| New Hampshire | Cheshire | 42.92 |
| New Hampshire | Coos | 44.69 |
| New Hampshire | Grafton | 43.94 |
| New Hampshire | Hillsborough | 42.91 |
| New Hampshire | Merrimack | 43.30 |
| New Hampshire | Rockingham | 42.98 |
| New Hampshire | Strafford | 43.30 |
| New Hampshire | Sullivan | 43.36 |
| New York | Albany | 42.60 |
| New York | Allegany | 42.26 |
| New York | Bronx | 40.86 |
| New York | Broome | 42.16 |
| New York | Cattaraugus | 42.25 |
| New York | Cayuga | 42.92 |
| New York | Chautauqua | 42.23 |
| New York | Chemung | 42.14 |
| New York | Chenango | 42.50 |
| New York | Clinton | 44.74 |
| New York | Columbia | 42.25 |
| New York | Cortland | 42.60 |
| New York | Delaware | 42.20 |
| New York | Dutchess | 41.77 |
| New York | Erie | 42.76 |
| New York | Essex | 44.11 |
| New York | Franklin | 44.59 |
| New York | Fulton | 43.11 |
| New York | Genesee | 43.00 |
| New York | Greene | 42.28 |
| New York | Hamilton | 43.66 |
| New York | Herkimer | 43.42 |
| New York | Jefferson | 44.04 |
| New York | Kings | 40.65 |
| New York | Lewis | 43.78 |
| New York | Livingston | 42.73 |
| New York | Madison | 42.92 |
| New York | Monroe | 43.15 |
| New York | Montgomery | 42.90 |
| New York | Nassau | 40.75 |
| New York | New York | 40.80 |
| New York | Niagara | 43.20 |
| New York | Oneida | 43.24 |
| New York | Onondaga | 43.01 |
| New York | Ontario | 42.86 |
| New York | Orange | 41.40 |
| New York | Orleans | 43.25 |
| New York | Oswego | 43.43 |
| New York | Otsego | 42.63 |
| New York | Putnam | 41.43 |
| New York | Queens | 40.71 |
| New York | Rensselaer | 42.71 |
| New York | Richmond | 40.58 |
| New York | Rockland | 41.15 |
| New York | Saratoga | 43.10 |
| New York | Schenectady | 42.82 |
| New York | Schoharie | 42.59 |
| New York | Schuyler | 42.40 |
| New York | Seneca | 42.78 |
| New York | St. Lawrence | 44.49 |
| New York | Steuben | 42.27 |
| New York | Suffolk | 40.87 |
| New York | Sullivan | 41.72 |
| New York | Tioga | 42.17 |
| New York | Tompkins | 42.45 |
| New York | Ulster | 41.89 |
| New York | Warren | 43.56 |
| New York | Washington | 43.32 |
| New York | Wayne | 43.16 |
| New York | Westchester | 41.17 |
| New York | Wyoming | 42.71 |
| New York | Yates | 42.64 |
| Vermont | Addison | 44.03 |
| Vermont | Bennington | 43.03 |
| Vermont | Caledonia | 44.46 |
| Vermont | Chittenden | 44.46 |
| Vermont | Essex | 44.73 |
| Vermont | Franklin | 44.85 |
| Vermont | Grand Isle | 44.79 |
| Vermont | Lamoille | 44.60 |
| Vermont | Orange | 44.00 |
| Vermont | Orleans | 44.83 |
| Vermont | Rutland | 43.58 |
| Vermont | Washington | 44.27 |
| Vermont | Windham | 42.99 |
| Vermont | Windsor | 43.58 |

Table S6. Total tick-borne disease case counts for Connecticut, Maine, New Hampshire, New York, and Vermont summarized from data obtained from the Johns Hopkins Lyme and Tickborne Diseases Dashboard.

| Year | Lyme disease | Anaplasmosis | Babesiosis |
| --- | --- | --- | --- |
| 2000 | 8185 |  |  |
| 2001 | 7813 |  |  |
| 2002 | 10545 |  |  |
| 2003 | 7156 |  |  |
| 2004 | 6902 |  |  |
| 2005 | 7884 |  |  |
| 2006 | 7274 |  |  |
| 2007 | 8082 |  |  |
| 2008 | 12957 | 332 |  |
| 2009 | 11474 | NA |  |
| 2010 | 7491 | 297 |  |
| 2011 | 9025 | 567 | 516 |
| 2012 | 7336 | 590 | 407 |
| 2013 | 10886 | 825 | 866 |
| 2014 | 8721 | 917 | 763 |
| 2015 | 9249 | 1338 | 1026 |
| 2016 | 8692 | 1505 | 862 |
| 2017 | 11395 | NA | 1223 |
| 2018 | 8835 | 1951 | 1048 |
| 2019 | 10388 | 3516 | 1224 |

Table S7. Tick-borne disease case counts by state summarized from data obtained from the Johns Hopkins Lyme and Tickborne Diseases Dashboard.

| Disease | Year | Connecticut | Maine | New Hampshire | New York | Vermont |
| --- | --- | --- | --- | --- | --- | --- |
| Lyme disease | 2000 | 3662 | 70 | 84 | 4329 | 40 |
|  | 2001 | 3475 | 108 | 129 | 4083 | 18 |
|  | 2002 | 4496 | 216 | 261 | 5535 | 37 |
|  | 2003 | 1351 | 173 | 190 | 5399 | 43 |
|  | 2004 | 1301 | 225 | 226 | 5100 | 50 |
|  | 2005 | 1753 | 247 | 265 | 5565 | 54 |
|  | 2006 | 1756 | 336 | 617 | 4460 | 105 |
|  | 2007 | 2354 | 529 | 896 | 4165 | 138 |
|  | 2008 | 2261 | 904 | 1594 | 7794 | 404 |
|  | 2009 | 3030 | 970 | 1415 | 5651 | 408 |
|  | 2010 | 1620 | 751 | 1339 | 3425 | 356 |
|  | 2011 | 1607 | 1006 | 1299 | 4490 | 623 |
|  | 2012 | 1255 | 1111 | 1450 | 2998 | 522 |
|  | 2013 | 2328 | 1373 | 1679 | 4615 | 891 |
|  | 2014 | 2311 | 1401 | 674 | 3736 | 599 |
|  | 2015 | 2508 | 1201 | 516 | 4314 | 710 |
|  | 2016 | 1728 | 1487 | 852 | 3882 | 743 |
|  | 2017 | 1983 | 1850 | 1318 | 5155 | 1089 |
|  | 2018 | 1843 | 1402 | 1377 | 3638 | 575 |
|  | 2019 | 1226 | 2166 | 1689 | 4243 | 1064 |
|  |  |  |  |  |  |  |
| Anaplasmosis | 2008 | 45 | 17 | 14 | 256 | 0 |
|  | 2009 | 22 | 15 | 18 | 250 | NA |
|  | 2010 | 43 | 17 | 20 | 215 | 2 |
|  | 2011 | 152 | 26 | 31 | 350 | 8 |
|  | 2012 | 142 | 52 | 52 | 335 | 9 |
|  | 2013 | 125 | 94 | 88 | 477 | 41 |
|  | 2014 | 75 | 191 | 131 | 453 | 67 |
|  | 2015 | 120 | 186 | 110 | 783 | 139 |
|  | 2016 | 103 | 372 | 54 | 775 | 201 |
|  | 2017 | 81 | 663 | 317 | NA | 399 |
|  | 2018 | 102 | 476 | 214 | 915 | 244 |
|  | 2019 | 298 | 685 | 301 | 1697 | 535 |
|  |  |  |  |  |  |  |
| Babesiosis | 2011 | 74 | 9 | 13 | 418 | 2 |
|  | 2012 | 123 | 10 | 19 | 253 | 2 |
|  | 2013 | 268 | 36 | 22 | 534 | 6 |
|  | 2014 | 205 | 42 | 42 | 471 | 3 |
|  | 2015 | 328 | 55 | 53 | 581 | 9 |
|  | 2016 | 322 | 82 | 13 | 430 | 15 |
|  | 2017 | 309 | 118 | 78 | 696 | 22 |
|  | 2018 | 248 | 101 | 37 | 641 | 21 |
|  | 2019 | 326 | 138 | 63 | 663 | 34 |

Figure S1. Nymph *Ixodes scapularis* abundance predictions from linear models used for nymph abundance in the northeastern United States at the average county centroid latitude for each state. Blue lines are the start year of data collection in each state, and red lines are the end year of data collection in each state. States without red lines are where data collection that was a part of these models was still ongoing, and Maine has data throughout the time period.


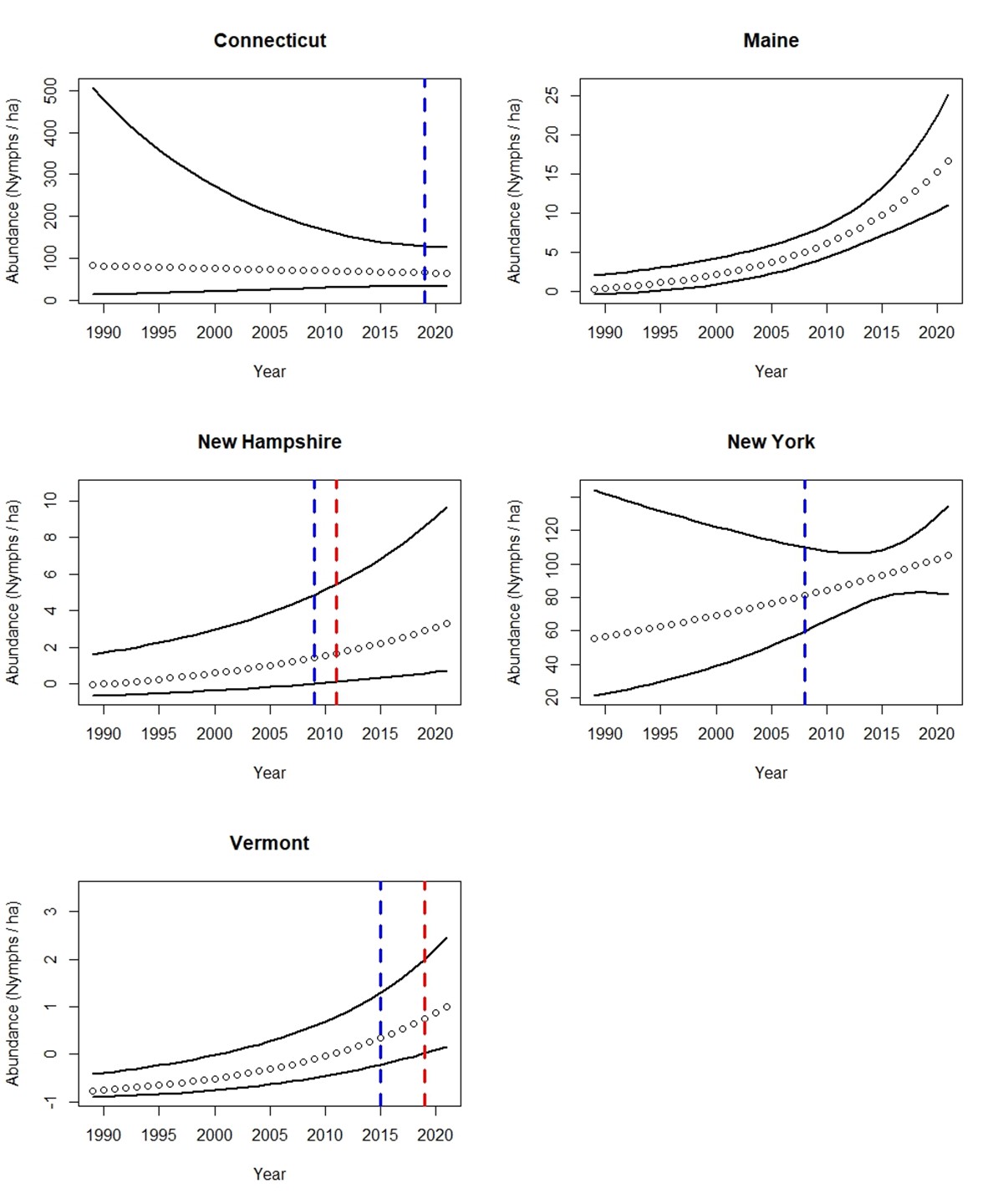


Figure S2. Adult *Ixodes scapularis* abundance predictions from linear models used for adult abundance in the northeastern United States at the average county centroid latitude for each state. Blue lines are the start year of data collection in each state, and red lines are the end year of data collection in each state. States without red lines are where data collection that was a part of these models was still ongoing, and Maine has data throughout the time period.


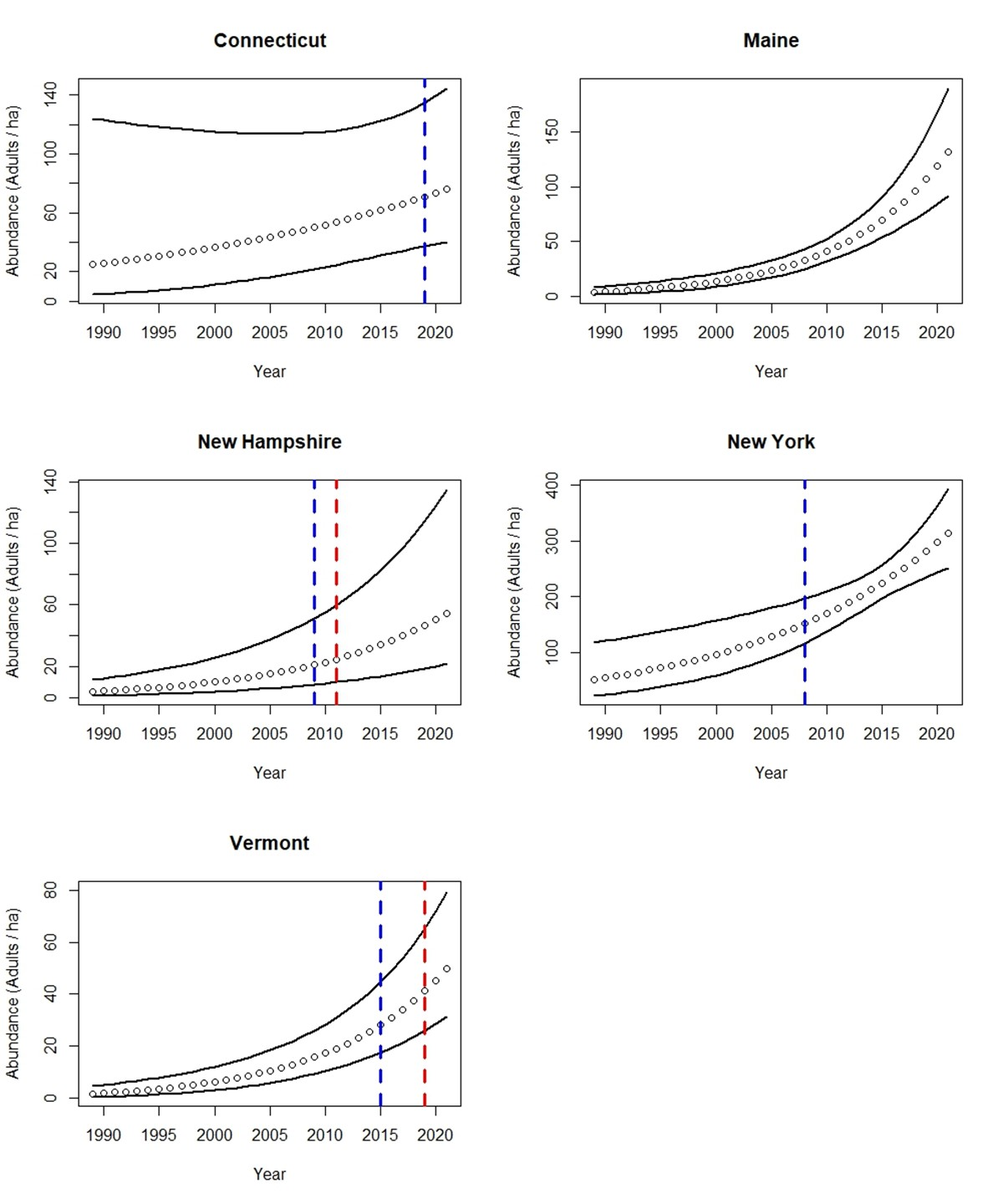

Supplement: Supplementary file 1 — Additional file 1: Table S1. Ixodes scapularis nymph abundance dataset created in the manuscript. Table S2. Ixodes scapularis adult abundance dataset created in the manuscript. Table S3. Ixodes scapularis nymph pathogen prevalence dataset created in the manuscript. Table S4. Ixodes scapularis adult pathogen prevalence dataset created in the manuscript. Table S5. County centroid latitudes in decimal degrees for all counties where Ixodes scapularis were collected. Table S6. Total tick-borne disease case counts across states evaluated in the manuscript summarized from data obtained from the Johns Hopkins Lyme and Tickborne Diseases Dashboard. Table S7. Tick-borne disease case counts by state summarized from data obtained from the Johns Hopkins Lyme and Tickborne Diseases Dashboard. Figure S1. Ixodes scapularis nymph abundance predictions by state from linear models. Figure S2. Ixodes scapularis adult abundance predictions by state from linear models. [file 13071_2024_6518_MOESM1_ESM.docx]
